# The role of microglial Tim-3 in neuroinflammation and functional recovery after spinal cord injury

**DOI:** 10.3389/fneur.2026.1828458

**Published:** 2026-06-19

**Authors:** Renjie Ji, Yikun Fu, Fangjun Jiao, Mingkang Zhang, Cuiping Tian, Xiaonan Han, Liyan Yan, Hongwei Kou, Junmin Wang, Hongjian Liu, Chao Jiang, Jian Wang, Tian Cheng

**Affiliations:** 1 Department of Orthopaedics, The First Affiliated Hospital of Zhengzhou University, Zhengzhou, Henan, China; 2 Department of Traditional Chinese Medicine, School of Traditional Chinese Medicine, Henan University of Chinese Medicine, Zhengzhou, Henan, China; 3 Department of Human Anatomy, School of Basic Medical Sciences, Zhengzhou University, Zhengzhou, Henan, China; 4 Department of Neurology, People's Hospital of Zhengzhou University, Zhengzhou, Henan, China; 5 Aging Decoding and Regeneration Institute, School of Basic Medical Sciences, Nanozyme Laboratory in Zhongyuan, State Key Laboratory of Antiviral Drugs, Pingyuan Laboratory, Zhengzhou University, Zhengzhou, Henan, China

**Keywords:** aav, microglia, neuroinflammation, spinal cord injury, T-cell immunoglobulin and mucin domain-containing molecule 3

## Abstract

**Aims:**

T-cell immunoglobulin and mucin domain-containing molecule 3 (Tim-3), an immune checkpoint molecule, is highly expressed in microglia and its expression dynamically increases during central nervous system (CNS) development. Although its immunomodulatory functions are well-established, its role in inflammation following spinal cord injury (SCI) remains unclear. This study aimed to elucidate the regulatory role of microglial Tim-3 in the sterile inflammatory response after SCI and to explore its potential as a therapeutic target.

**Materials and methods:**

A SCI model was established using C57BL/6 mice. Microglial Tim-3 function was investigated through adeno-associated virus-mediated Tim-3 overexpression and intervention with the Nrf2 agonist Oltipraz. Luxol fast blue (LFB) and Nissl staining were used to assess lesional area and tissue structure. Basso Mouse Scale (BMS) scoring and the sucrose preference test (SPT) were employed to evaluate motor function recovery and depressive-like behavior. Immunofluorescence was performed to analyze glial activation and neurodegeneration. Expression levels of inflammatory factors were measured by enzyme-linked immunosorbent assay (ELISA) and western blot (WB).

**Key findings:**

Microglia-specific Tim-3 overexpression promoted microglial proliferation and activation, inducing upregulation of iNOS and robust production of pro-inflammatory cytokines. This exacerbated neural tissue damage and motor dysfunction, whereas depressive-like behaviors were not significantly affected. These effects were partially reversed by the Nrf2 agonist.

**Significance:**

AAV-mediated microglial Tim-3 overexpression exacerbates neuroinflammation and functional impairment after SCI, potentially through an association with the Nrf2/HMGB1 signaling axis. Targeting microglial Tim-3 may represent a promising therapeutic strategy for SCI.

## Graphical abstract

**Figure:**
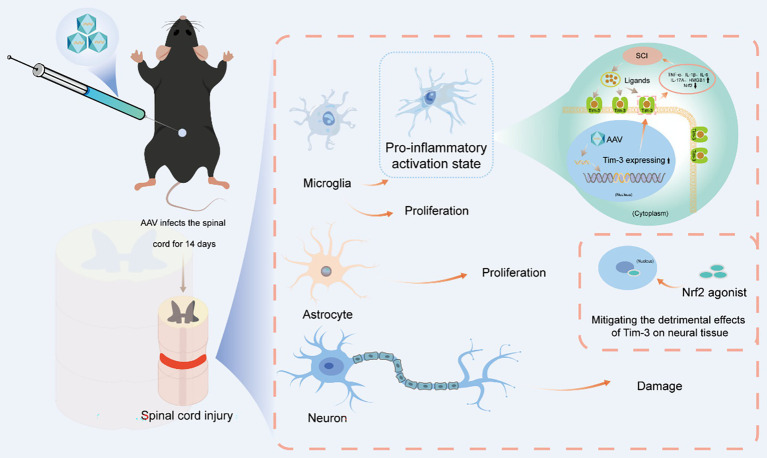

## Highlights

- Microglial Tim-3 promotes microglial proliferation, activation, and pro-inflammatory status following spinal cord injury.
- Microglial Tim-3 exacerbates neuroinflammation and impedes neural tissue repair following spinal cord injury.
- The effects mediated by microglial Tim-3 may be associated with the Nrf2/HMGB1 signaling axis.

## 1 Introduction

Spinal cord injury (SCI) is a devastating traumatic disorder of the central nervous system (CNS) that often leads to irreversible neurological dysfunction. Epidemiological data indicate a substantial burden, with approximately 759,302 total traumatic SCl cases in China and 66,374 new cases reported annually, primarily due to traffic accidents, falls, and violence (1). Clinically, SCI presents with a range of symptoms, including motor and sensory deficits, bladder incontinence, and autonomic dysregulation below the level of injury (2). These impairments severely compromise patients’ quality of life and impose a substantial economic burden on families and the healthcare system (3). SCI progresses through two critical phases: primary and secondary injury. Following primary damage, secondary injury comprises a complex cascade of molecular and cellular events, including spinal cord edema, neuroinflammation, immune cell infiltration, mitochondrial dysfunction, programmed cell death, and oxidative stress. These processes collectively exacerbate tissue damage and impede neural regeneration (4–6). Despite extensive research, there is currently no effective clinical treatment to fully restore neurological function after SCI. Nevertheless, early modulation of inflammatory responses has been shown to mitigate tissue damage, promote microenvironmental remodeling, and improve functional recovery. Therefore, elucidating the molecular mechanisms of SCI and developing targeted therapeutic strategies remain pressing challenges in neuroscience research (7).

In response to traumatic stimuli such as SCI, microglia undergo polarization into distinct M1 and M2 phenotypes (8). After SCI, the microglial population progressively increases. Initially, microglia rapidly polarize toward a pro-inflammatory phenotype, which subsequently shifts toward an anti-inflammatory phenotype as the pathology evolves (9). During this process, resident microglia undergo morphological changes—including cell body hypertrophy and process retraction—adopting an amoeboid shape characteristic of the activated M1 state. These activated microglia secrete pro-inflammatory cytokines, such as tumor necrosis factor-alpha (TNF-α), interleukin-1beta (IL-1β), and IL-6. This sustained inflammatory response contributes to extensive neuronal death, axonal degeneration, and demyelination, thereby exacerbating neural tissue damage and impeding functional recovery (10). In contrast, M2 microglia help suppress neuroinflammation and promote neural repair post-SCI (11). Although the M1 phenotype predominates after injury, studies have demonstrated that microglial depletion hinders functional recovery, underscoring their complex dual role in both propagating inflammation and enabling tissue repair (12–14). Therefore, therapeutic strategies designed to modulate microglial polarization by favoring the M2 phenotype present a promising avenue for SCI treatment (15). Recent studies, however, have emphasized that the response state of microglia *in vivo* is highly heterogeneous and dynamic, and cannot be simply encapsulated by the classical M1/M2 binary paradigm; instead, they exhibit multiple mixed activation spectra, with their functions collectively determined by complex signals from the local microenvironment (16). This study aims to investigate how microglial T-cell immunoglobulin and mucin domain-containing molecule 3 (Tim-3) regulates their complex activation states after SCI, particularly its effect on the expression of pro-inflammatory (e.g., iNOS^+^) and repair-associated (e.g., Arg-1^+^) markers, and how this regulation influences neuroinflammation and functional recovery.

Tim-3 is a critical immune checkpoint molecule. Initial studies demonstrated that Tim-3 was exclusively expressed on CD4^+^ Th1 cells but absent in Th2 cells (17). Subsequent studies revealed its broader expression on monocytes/macrophages, mast cells, dendritic cells, and microglia (18, 19). Tim-3 binds to various ligands, mediating effects such as promoting Th1 cell apoptosis, suppressing innate immune responses, inducing CD8^+^ T cell exhaustion, and facilitating apoptotic cell clearance (20). Initially, Tim-3 was characterized as a negative regulator (21). Tim-3 has recently been found to be expressed in the CNS, predominantly on microglia, with minimal to undetectable levels in neurons and astrocytes.

Nuclear factor erythrocyte 2–associated factor 2 (Nrf2) is a neuroprotective transcription factor involved in numerous physiological processes, such as oxidative stress, DNA repair, mitochondrial membrane stability maintenance, and inflammatory responses, where it exerts protective effects (22). In SCI, multiple factors contribute to reactive oxygen species (ROS) generation, exacerbating neural tissue damage (23). Under oxidative stress, Nrf2 translocates from the cytoplasm to the nucleus, upregulating genes such as superoxide dismutase (SOD), catalase (CAT), and heme oxygenase-1 (HO-1). SOD and CAT are then transported into the cytoplasm to exert their antioxidant functions (24). Additionally, following SCI, activation of Nrf2 can mitigate the inflammatory response and protect neural cells by modulating the activation of microglia and other relevant cells (25). The relationship between the pro-inflammatory potential of microglial Tim-3 and the protective Nrf2 pathway in SCl, however, is unexplored.

In contrast to its protective role in autoimmunity, Tim-3 exerts a pro-inflammatory function in CNS disorders such as ischemic stroke and subarachnoid hemorrhage (26, 27). This functional dichotomy is likely influenced by cell type-specificity, the local microenvironment, and incompletely understood molecular mechanisms. Although accumulating evidence implicates Tim-3 in neuroinflammation, its precise role in the inflammatory cascade following SCI remains elusive. Therefore, this study aims to investigate how microglial Tim-3 regulates neuroinflammatory responses following SCI and to explore its potential interaction with the Nrf2/HMGB1 signaling axis. Using an established murine SCI model, we achieved microglia-specific Tim-3 overexpression via adeno-associated virus (AAV) to delineate its functional consequences. Our findings provide insights that could inform the development of novel therapeutic strategies for SCI.

## 2 Materials and methods

### 2.1 Animals

Male C57BL/6 mice (10–12 weeks old; 20–25 g) were obtained from the Laboratory Animal Center of Zhengzhou University. Mice were housed individually under specific pathogen-free (SPF) conditions in a controlled environment (temperature: 21–25 °C; 12/12 h light/dark cycle) with ad libitum access to food and water. All experimental procedures were approved by the Institutional Animal Care and Use Committee of Zhengzhou University and conducted in accordance with the ARRIVE guidelines to ensure ethical and standardized practices (Approval Number: 2021-KY-0260-003).

### 2.2 SCI mouse model

Surgical procedures were performed 14 days after AAV injection to ensure optimal viral transduction efficiency. Mice were anesthetized via intraperitoneal injection of 1% pentobarbital sodium. A dorsal midline incision was made to expose the T9-T11 vertebrae. After removing the paravertebral muscles and fascial tissues, a T10 laminectomy was performed to expose the spinal cord. The dura mater remained intact. Spinal cord compression was induced by applying a 1.8 cm microvascular clip (with a closing force equivalent to 20 g) at the T10 level for 5 s. Successful injury was confirmed by observing transient hind limb and tail spasms upon clip application, followed by the immediate formation of a distinct hematoma, flaccid paralysis of the hind limbs, and sensory loss upon clip removal. The muscle and skin layers were then sutured sequentially with 4–0 silk. Sham-operated mice underwent the same surgical procedures, including laminectomy, but did not receive spinal cord compression. Postoperatively, animals were kept on a thermostatically controlled heating pad until fully recovered from anesthesia. Manual bladder emptying was performed twice daily until the return of spontaneous micturition.

### 2.3 Experimental groups and treatment protocols

Mice were randomly assigned to experimental groups using computer-generated randomization. The study consisted of two parts. In Part I, animals were divided into four groups: (1) Sham, (2) SCI + Vehicle (Veh), (3) SCI + AAV-Negative Control (NC), and (4) SCI + AAV-Tim-3. Part II also included four groups: (1) Sham, (2) SCI + Veh, (3) SCI + AAV-Tim-3, and (4) SCI + AAV-Tim-3 + Oltipraz (10 mg/kg). Previous studies have confirmed that this serotype of AAV does not affect the transcriptome, cellular status, or responsiveness to external factors of microglia *in vivo* (28). This virus is derived from AAV9, and in models of central nervous system injury, tissue- and cell-level outcomes in AAV9-treated groups showed no significant differences compared with vehicle-treated controls (29, 30). Furthermore, in the present study, no statistically significant differences in inflammatory factor levels were observed between the negative control AAV group and the untreated group. Therefore, the SCI + AAV group was not included in LFB staining, Nissl staining, or immunofluorescence analyses; only the SCI + Veh and SCI + AAV-Tim-3 groups were used. All assessments were conducted by investigators blinded to the experimental groups.

To achieve microglia-specific Tim-3 overexpression, we utilized a novel AAV serotype MG1.2 driven by a CD68 promoter, encoding eGFP and Flag tags for transduction verification. This system enables efficient microglial transduction without inducing cellular activation (28).

Two weeks prior to SCI induction, mice received intrathecal injections of either AAV-Tim-3 or AAV-NC (GeneChem, Shanghai, China). The injection procedure was as follows: mice anesthetized with 1% sodium pentobarbital were securely positioned. After shaving and disinfecting the skin over the iliac crest, a 27-gauge needle connected to a 50-μL Hamilton syringe was inserted midline into the L5-L6 intervertebral space. Successful dural puncture was confirmed by a brief tail-flick reflex, whereupon 8 μL PBS containing 4 × 10^9^ viral genome AAV particles were slowly administered (31). The needle was maintained *in situ* for 1 min post-injection to prevent reflux.

The Nrf2 agonist Oltipraz was dissolved in vehicle solution (5% DMSO, 40% PEG300, 5% Tween 80, and 50% ddH₂O) and administered intraperitoneally three times over two days preceding surgery, with the final dose given 1 h before SCI induction (32).

### 2.4 Tissue processing

Mice were deeply anesthetized and transcardially perfused with ice-cold phosphate-buffered saline (PBS) followed by 4% paraformaldehyde (PFA) in PBS. Spinal cord segments containing the lesion epicenter were carefully dissected and post-fixed in the same fixative for 48 h at 4 °C. Tissues were then cryoprotected through graded sucrose dehydration: first in 20% sucrose for 48 h, then in 30% sucrose for 72 h, both at 4 °C. After complete dehydration, tissues were embedded in Optimal Cutting Temperature (OCT) compound (Sakura) and frozen. Serial sections of 30 μm thickness were cut using a cryostat maintained at −20 °C and stored at −80 °C for subsequent analysis.

### 2.5 Luxol fast blue staining

To evaluate demyelination, spinal cord sections collected at 7 and 28 days post-SCI were subjected to Luxol Fast Blue (LFB, Sigma, S3382) staining. The staining procedure was performed as follows: frozen sections were gradually equilibrated to room temperature through sequential incubation at −20 °C, 4 °C, and 25 °C, then dried overnight at 37 °C. Sections were immersed in preheated LFB staining solution (60 °C) for 2 h, followed by thorough rinsing in distilled water. Differentiation was performed in lithium carbonate solution and ethanol series. After dehydration through graded alcohols, sections were cleared in xylene and mounted with neutral resin. The demyelinated lesion area was quantified using SigmaScan Pro software.

### 2.6 Nissl staining

Cryopreserved spinal cord sections stored at −80 °C were gradually equilibrated to room temperature by sequential transfer to −20 °C, 4 °C, and 25 °C environments. Sections were then stained with pre-warmed (37 °C) Nissl staining solution (Beyotime, C0117) for 5 min to specifically visualize Nissl bodies in neuronal cells. After staining, sections were rinsed sequentially with ddH₂O, 95% ethanol, and absolute ethanol for dehydration. Following xylene clearance for 5 min, sections were mounted with neutral resin. Images were captured using a Nikon microscope (Tokyo, Japan), and Nissl-positive cells were quantified and averaged using ImageJ Cell Counter.

### 2.7 Spinal cord water content

At 3 days post-SCI, mice were anesthetized with pentobarbital sodium and euthanized. A 1 cm spinal cord segment centered on the lesion epicenter (0.5 cm rostral and caudal) was rapidly dissected. The wet weight (WW) of each sample was immediately measured using a precision electronic balance. Samples were then dried in a 100 °C oven for 48 h to obtain the dry weight (DW). The percentage of water content was calculated using the following formula:


Water content(%)=[(WW−DW)/WW]×100%

### 2.8 Basso Mouse Scale scoring

Hindlimb motor recovery after SCI was evaluated using the Basso Mouse Scale (BMS). Two independent investigators blinded to the experimental groups assessed mice in an open field at baseline (day 0, pre-injury) and on post-injury days 1, 3, 7, 14, and 28. The BMS score, ranging from 0 (complete hindlimb paralysis) to 9 (normal locomotion), was determined based on parameters including joint movement, trunk stability, forelimb-hindlimb coordination, stepping, toe clearance, and tail position.

### 2.9 Sucrose preference test (SPT)

Depressive-like behavior was assessed using the SPT, where a reduced preference for sucrose solution is indicative of anhedonia. After a 24-h period of food and water deprivation, mice were habituated for 24 h with two pre-weighed bottles—one containing 1% sucrose solution and the other plain water—with their positions switched after 12 h to prevent side preference. Following habituation, mice were subjected to a 48-h test under the same two-bottle choice condition. The bottles were weighed again at the end of the test. Sucrose preference was calculated as follows:

$\text{Sucrose preference}\;(\%)=[\begin{array}{l} \text{Sucrose intake}\;(g)\\ /(\begin{array}{l} \text{Sucrose intake}\;(g)\\ +\text{Water intake}\;(g) \end{array}) \end{array}]\times 100\%$

### 2.10 Immunofluorescence

Immunofluorescence was performed on day 7 post-SCI. Three evenly spaced sections from the lesion center of each mouse were selected. The staining procedure was as follows: (1) three 5-min PBS washes; (2) 2-h blocking with 1% BSA at room temperature; (3) overnight incubation at 4 °C with primary antibodies diluted in antibody dilution buffer. Primary antibodies included: rabbit anti-GFAP (1:200; Proteintech, 16,825-1-AP), rabbit anti-Iba1 (1:1000; Dako, 019–19,741), and rabbit anti-NeuN (1:500; Proteintech, 26,975-1-AP). After primary antibody incubation, sections were washed three times with PBST and incubated with CoraLite594-conjugated goat anti-rabbit IgG (H + L) (1:500; Proteintech, SA00013-4) for 1 h at room temperature. Sections were then washed again, counterstained with DAPI (1:100; Solarbio, C0060) for 15 min, and mounted with anti-fade medium. Imaging was performed using a Nikon Ni-U fluorescence microscope. For each mouse, 12 fields (4 fields/section × 3 sections) were captured at 20× magnification. Positively stained cells were quantified and averaged using Adobe Photoshop.

### 2.11 Western blotting (WB)

Spinal cord tissues were collected 3 days post-SCI for western blotting. Mice were anesthetized and transcardially perfused with ice-cold PBS. A 4-mm segment centered on the lesion epicenter (2 mm rostral and caudal) was rapidly dissected and flash-frozen in liquid nitrogen. Total protein was extracted using RIPA buffer supplemented with PMSF protease inhibitor (Solarbio; 100:1 ratio). Protein concentration was determined using an enhanced BCA assay kit (CWBIO, CW0014S). After denaturation at 100 °C for 10 min, equal amounts of protein were separated by 15% SDS-PAGE and transferred to PVDF membranes. Membranes were blocked for 10 min (Servicebio, G2052) and incubated overnight at 4 °C with the following primary antibodies: mouse anti-β-actin (β-actin, 1:20000; Proteintech, 66,009-1-Ig), Tim-3 Monoclonal antibody (1:5000, Proteintech, 60,355-1-Ig), GFP tag Polyclonal antibody (1:2000, Proteintech,50,430-2-AP), TNF-alpha Polyclonal antibody (1:1000, Proteintech,17,590-1-AP), IL-1 beta Polyclonal antibody (1:3000, Proteintech, 29,530-1-AP), IL-6 Polyclonal antibody (1:1000, Proteintech,21,865-1-AP), HMGB1 Polyclonal antibody (1:10000, Proteintech, 10,829-1-AP), IL-17A Monoclonal antibody (1:2000, Proteintech,66,148-1-Ig), iNOS Polyclonal antibody (1:2000, Proteintech, 18,985-1-AP), Arginase-1 Monoclonal antibody (1:10000, Proteintech,66,129-1-Ig), NRF2 Recombinant antibody (1:2500, Proteintech, 80,593-1-RR). After three 10-min TBST washes, membranes were incubated with goat anti-mouse (1:10,000; Proteintech, SA00001-1) or goat anti-rabbit (1:10,000; Proteintech, SA00001-2) secondary antibodies at 37 °C for 1 h. Protein bands were visualized using an ECL chemiluminescence kit (Affinity, KF001) and semi-quantitatively analyzed with ImageJ software.

### 2.12 Enzyme-linked immunosorbent assay (ELISA)

Spinal cord tissues were collected at 3 days post-SCI for ELISA. After anesthesia and transcardial perfusion with ice-cold PBS, tissue samples were homogenized and centrifuged at 12,000 × g for 20 min at 4 °C. The supernatant was collected, and concentrations of TNF-*α* and IL-1β were measured using commercial ELISA kits (Servicebio: GEM0004 for TNF-α, GEM0002 for IL-1β) according to the manufacturer’s instructions. All samples were run in duplicate, and absorbance was read at 450 nm using a microplate reader.

### 2.13 Statistics

Data were analyzed using GraphPad Prism 9.5 (GraphPad Software, San Diego, CA) and SPSS. Normally distributed data are expressed as mean ± SD, and non-normal data as median with interquartile range (IQR). The Kolmogorov–Smirnov test was used to assess normality, and Bartlett’s test was applied to evaluate homogeneity of variances. For two-group comparisons of normal data, a two-tailed Student’s *t*-test was used; multiple-group comparisons were performed by one-way ANOVA followed by Bonferroni’s *post hoc* test. Non-normal data were analyzed using the Mann–Whitney *U* test (two groups) or Kruskal–Wallis test with Dunn’s post hoc test (multiple groups). Longitudinal BMS scores were analyzed using generalized estimating equations (GEE). A *p* < 0.05 was considered statistically significant.

## 3 Results

### 3.1 Temporal expression profile of Tim-3 post-SCI

**Figure 1:**
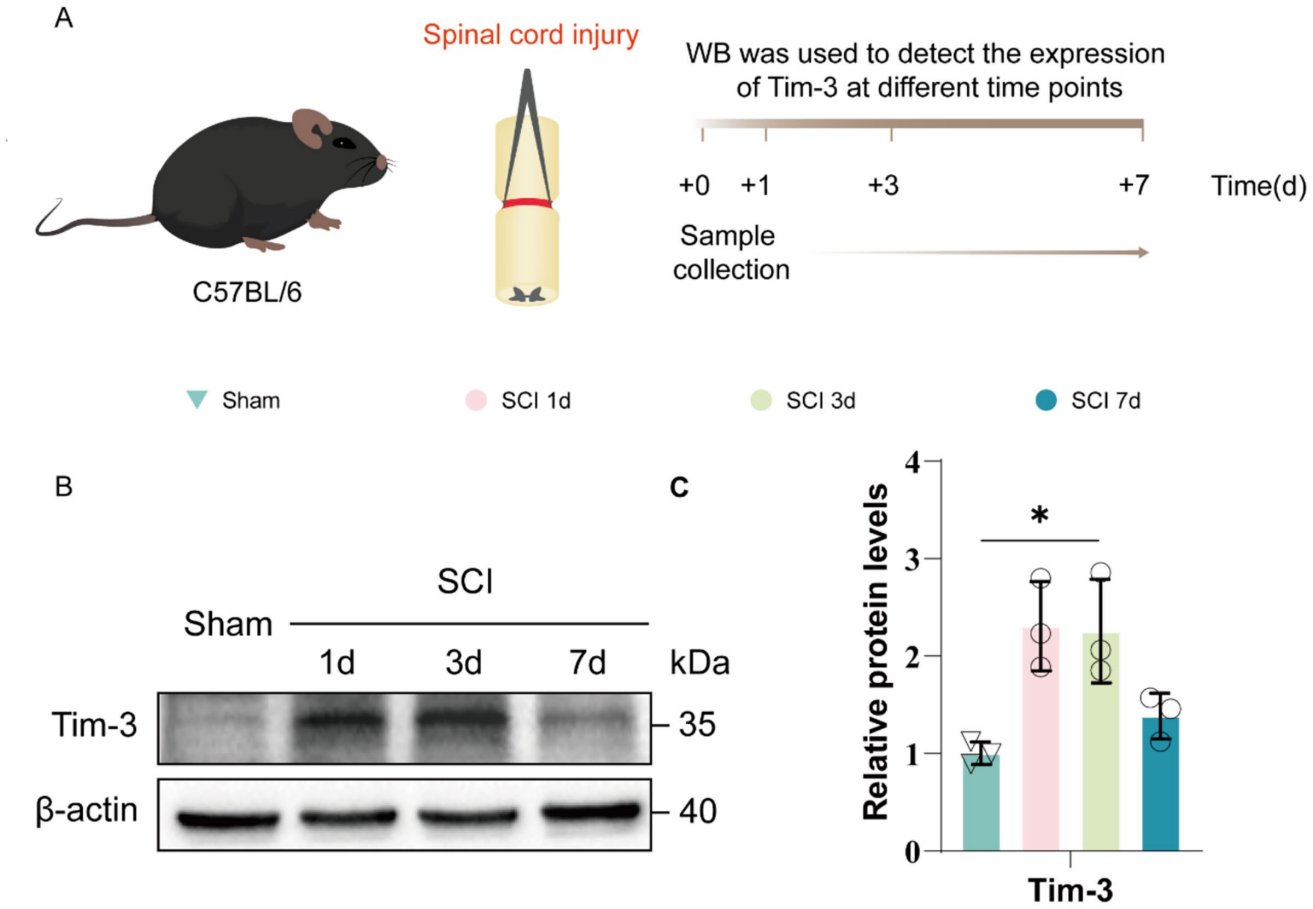
Temporal expression profile of Tim-3 post-SCI. **(A)** Workflow for handling mice. **(B)** Representative WB bands for Tim-3 from spinal cord protein samples. *β*-Actin serves as the loading control. **(C)** Scatter plots presenting the quantitative analysis of Tim-3 level (one-way ANOVA followed by Bonferroni *post hoc* test, *n* = 3 mice per group). All data are presented as mean ± SD. *p < 0.05, **p < 0.01.

WB analysis was performed to assess Tim-3 expression in sham-operated controls and at multiple time points after SCI. Tim-3 expression was significantly upregulated as early as day 1 post-SCI, peaked at 3 days (*F* = 8.885, *p* < 0.05; Figures 1A–C), and then declined by day 7, though it remained elevated compared to sham controls.

### 3.2 AAV-mediated, microglia-specific overexpression of Tim-3

**Figure 2:**
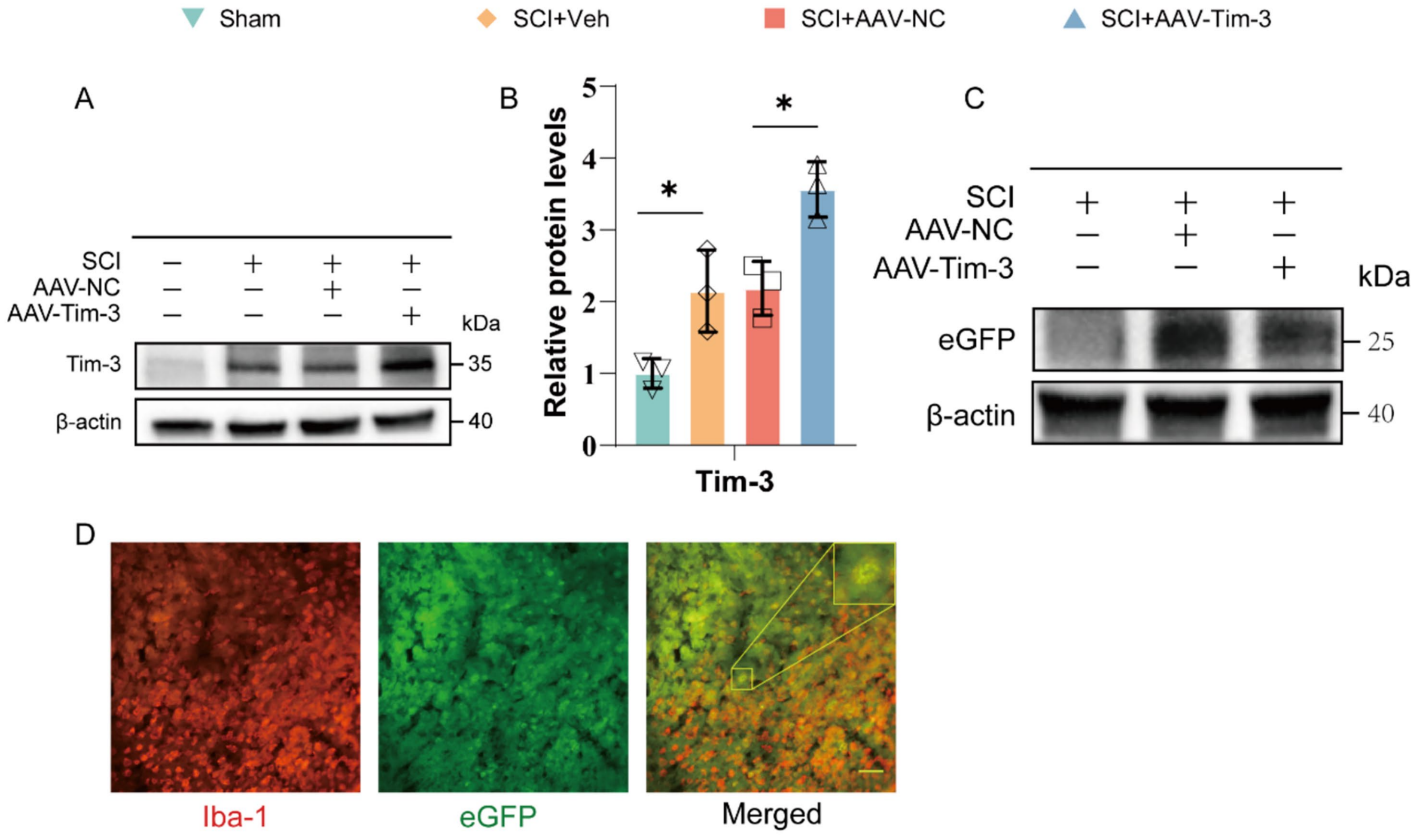
AAV-Tim-3 successfully infected microglia. **(A,C)** Representative WB bands of Tim-3 and eGFP in spinal cord protein samples on day 3 post-SCI. β-actin serves as the loading control. **(B)** Scatter plots presenting the quantitative analysis of Tim-3 level (one-way ANOVA followed by Bonferroni post hoc test, *n* = 3 mice per group). All data are presented as mean ± SD. *p < 0.05, **p < 0.01. **(D)** Representative immunofluorescence images demonstrating AAV-Tim-3-specific microglial transduction on day 7 post-SCI.

To investigate the functional role of microglial Tim-3, we employed an AAV serotype (MG1.2) under the control of the CD68 promoter to achieve cell-specific overexpression. WB analysis confirmed that AAV-Tim-3 injection significantly increased Tim-3 protein levels compared to both vehicle and AAV-NC groups (*F* = 20.268, *p* < 0.05; Figures 2A,B), whereas the negative control AAV-NC showed no such effect. WB analysis of eGFP further confirmed successful AAV transduction in both the AAV-NC and AAV-Tim-3 groups (Figure 2C). Immunofluorescence colocalization of eGFP (488 nm) with the microglial marker Iba-1 (594 nm) verified the microglia-specific tropism of the AAV vector (Figure 2D).

### 3.3 Microglial Tim-3 overexpression exacerbates early tissue damage and edema

**Figure 3:**
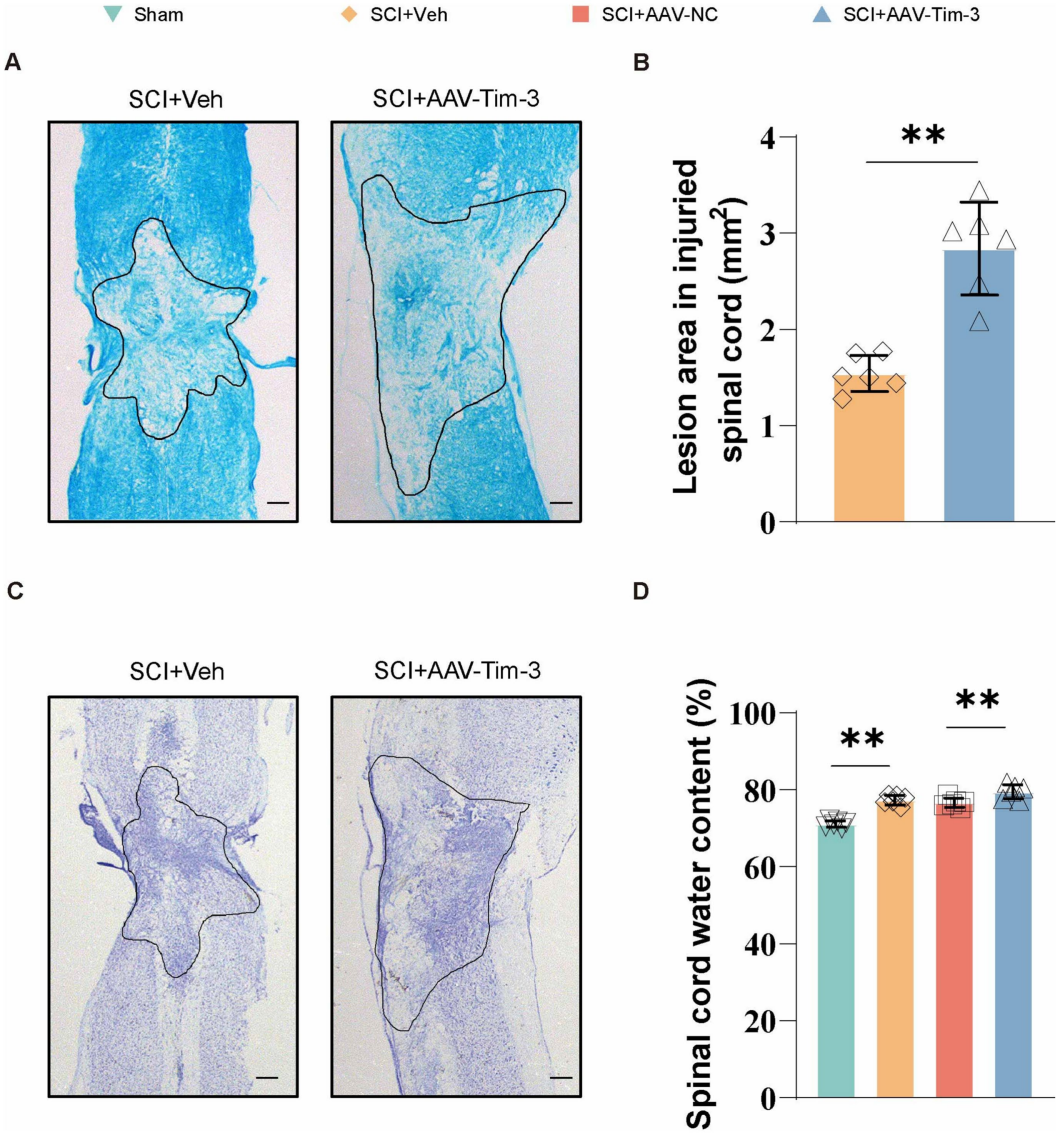
Overexpression of Tim-3 in microglia exacerbates spinal cord lesion area on day 7 as well as the severity of edema on day 3 post-SCI. **(A)** Representative images of spinal cord sections stained with LFB on day 7 post-SCI. The lesion area is outlined with a black curve; scale bar = 200 μm. **(B)** Statistical analysis shows that overexpression of Tim-3 in microglia exacerbated the spinal cord lesion area on day 7 post-SCI compared to the SCI group. An independent-samples *t*-test was used to analyze the SCI area, with *n* = 6 mice per group. **(C)** Representative spinal cord sections stained with Nissl on day 7 post-SCI. The lesion area is delineated by a black curve; scale bar = 200 μm. **(D)** On day 3 post-SCI, overexpression of Tim-3 in microglia exacerbates the water content in the injured spinal cord. One-way ANOVA followed by Bonferroni post hoc test is used to analyze spinal cord water content. *n* = 6 mice per group.

To evaluate the pathological consequences of Tim-3 overexpression, we first assessed tissue damage and edema in the acute phase. LFB staining at 7 days post-SCI revealed that the lesion area was significantly larger in the AAV-Tim-3 group compared to the vehicle-treated SCI group (t = 6.128, *p* < 0.001; Figures 3A–C). Consistent with aggravated tissue damage, spinal cord water content-an indicator of edema and blood-spinal cord barrier disruption-was also markedly higher in the AAV-Tim-3 group at 3 days post-injury (*F* = 43.429, *p* < 0.01; Figure 3D), indicating aggravated neurovascular damage and demonstrating that Tim-3 overexpression worsens spinal cord edema after SCI.

### 3.4 Microglial Tim-3 overexpression impairs motor recovery without affecting depressive-like behavior

**Figure 4:**
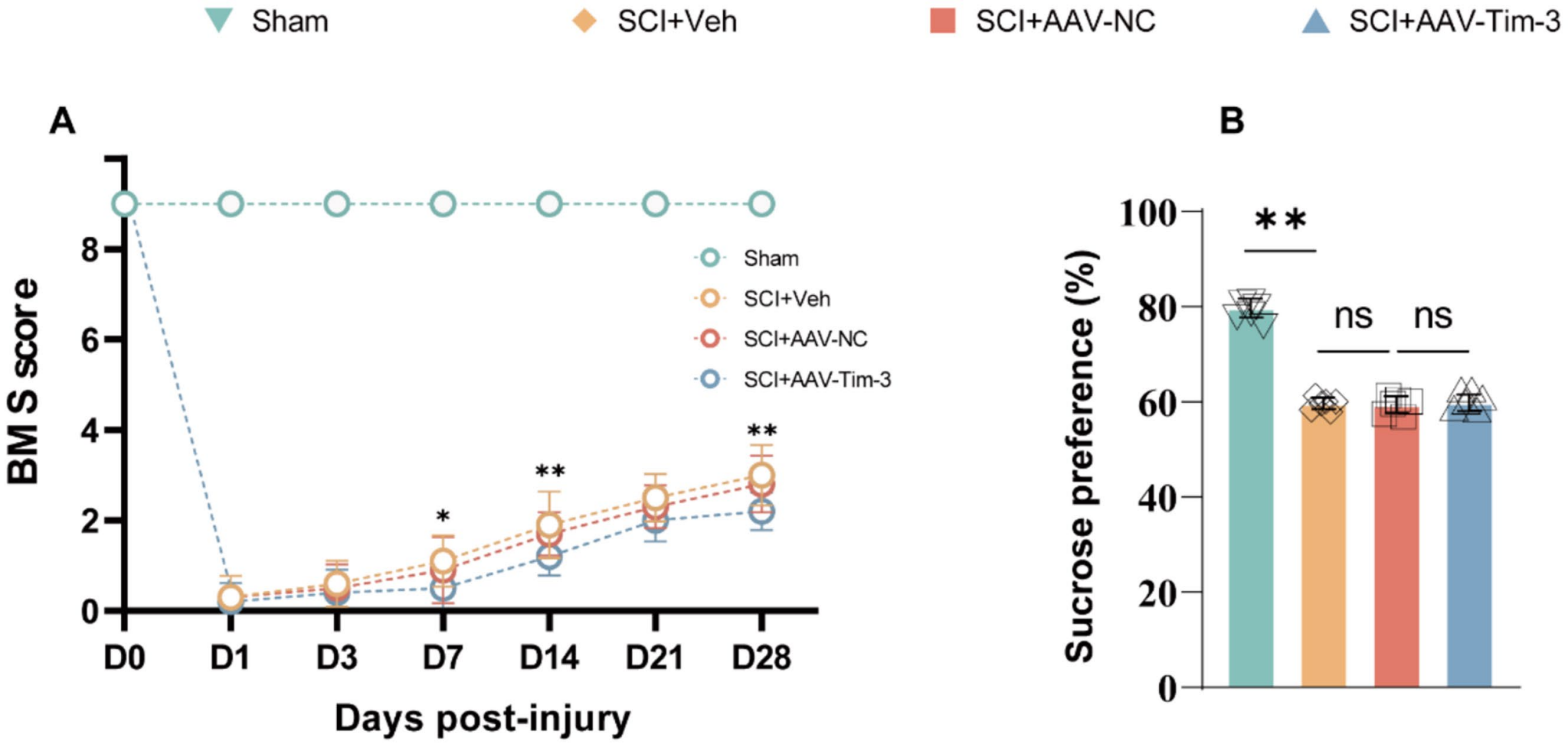
Overexpression of Tim-3 in microglia reduces BMS scores on day 7, 14, and 28 post-SCI, but does not alter depressive symptoms on day 28 post-SCI. **(A)** BMS scores were used to evaluate the recovery of motor function in all four groups. GEE are used for analysis. *n* = 10 mice per group. All data are presented as mean ± SD. **(B)** AAV-Tim-3 treatment did not aggravate the depressive symptoms in mice on day 28 post-SCI. One-way ANOVA followed by Bonferroni post hoc test is employed to analyze the percentage of sucrose intake in each group. *n* = 6 mice per group. *p < 0.05, **p < 0.01.

Motor function after SCI is an important indicator of neurological recovery. We assessed locomotor performance using the BMS on days 1, 3, 7, 14, 21, and 28 post-injury. All mice showed complete hindlimb paralysis (BMS score = 0) immediately after SCI. While motor function began to improve from day 3 onward in all groups, the AAV-Tim-3 group exhibited significantly lower BMS scores than the SCI + Veh group on days 7, 14, and 28 (Wald *χ*^2^ = 16.768, *p* < 0.05 for each time point; Figure 4A), indicating that microglial Tim-3 overexpression impedes long-term functional recovery.

Depressive-like behavior was evaluated using the SPT on day 28 post-SCI. No significant difference in sucrose preference was observed between the SCI + AAV-Tim-3 and SCI + AAV-NC groups (*F* = 207.300, *p* > 0.05; Figure 4B). These results suggest that although SCI itself can induce depressive-like behaviors, microglial Tim-3 overexpression does not significantly modulate such behavioral deficits.

### 3.5 Microglial Tim-3 overexpression worsens chronic tissue damage and neuronal loss

**Figure 5:**
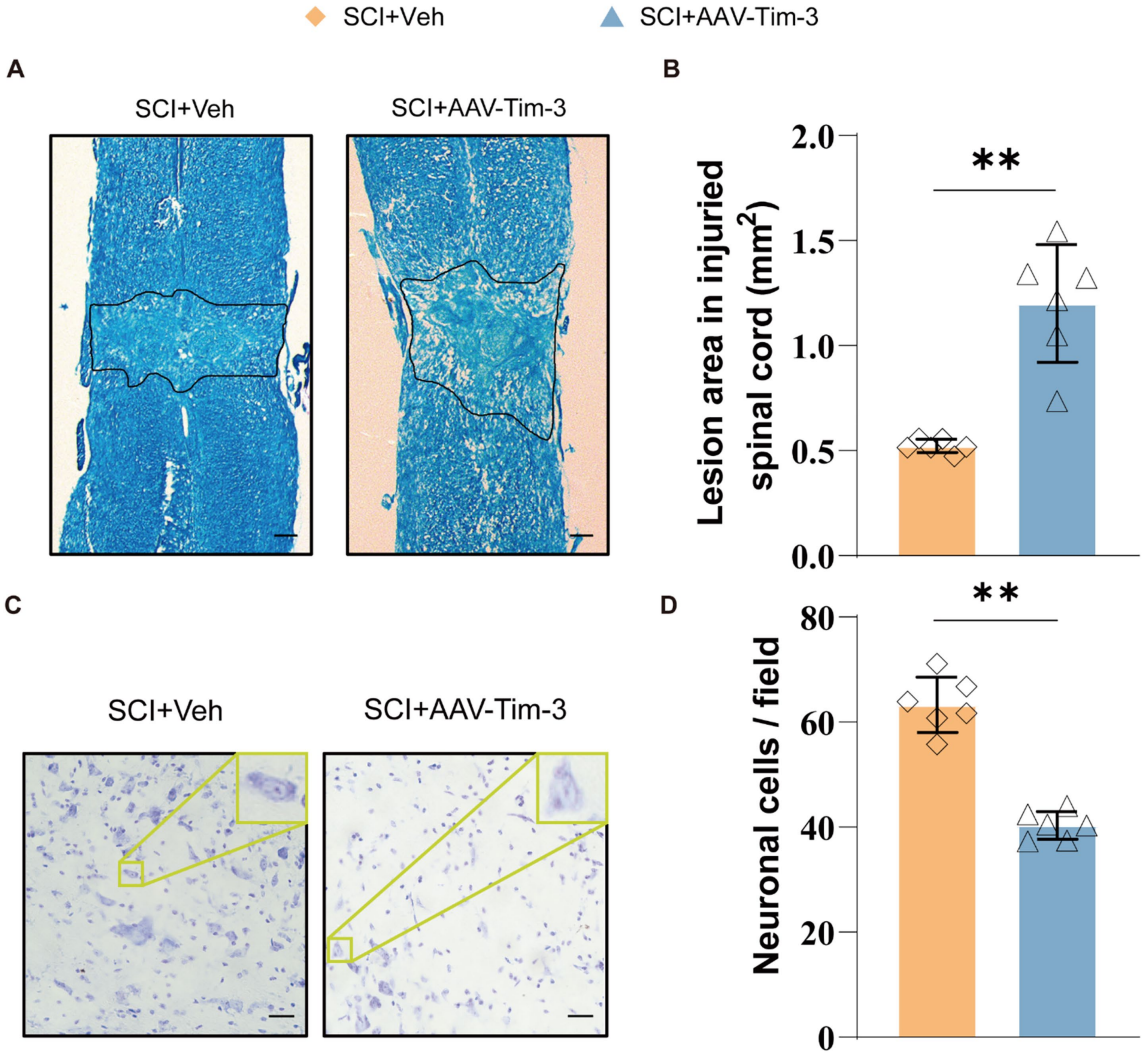
Overexpression of Tim-3 in microglia exacerbated spinal cord lesion area on day 28 post-SCI, and augmented neuronal death on day 28 post-SCI. **(A)** Representative images of spinal cord sections stained with LFB on day 28 post-SCI. The lesion area is outlined with a black curve; scale bar = 200 μm. **(B)** Statistical analysis shows that overexpression of Tim-3 in microglia exacerbated the spinal cord lesion area on day 28 post-SCI compared to the SCI group. An independent-samples *t*-test was used to analyze the SCI area, with *n* = 6 mice per group. **(C)** Representative images of Nissl staining around the injury site on day 28 post-SCI. The inset shows a high magnification of Nissl-positive cells. Scale bar = 50 μm. **(D)** Quantitative analysis of neurons. Overexpression of Tim-3 in microglia exacerbated the loss of Nissl bodies in the lesioned spinal cord. An independent-samples *t*-test was used to analyze the SCI area, with *n* = 6 mice per group. *p < 0.05, **p < 0.01.

The detrimental effects of Tim-3 overexpression persisted into the chronic phase. LFB staining at 28 days post-SCI showed that lesion areas remained significantly larger in the AAV-Tim-3 group (*t* = 5.902, *p* < 0.001; Figures 5A,B).

Correspondingly, Nissl staining demonstrated a significant reduction in the number of surviving neurons in the peri-lesion area of Tim-3-overexpressing mice (t = −9.535, *p* < 0.001; Figures 5C,D), indicating that microglial Tim-3 overexpression exacerbates chronic neuronal loss after SCI.

### 3.6 Microglial Tim-3 overexpression amplifies the pro-inflammatory cytokine response

During the early phase of SCI, microglia polarize predominantly toward the M1 phenotype, contributing significantly to neuroinflammation through the release of pro-inflammatory cytokines such as TNF-α, IL-1β, and IL-6. WB analysis on day 3 post-SCI revealed that the expression levels of TNF-α, IL-1β, IL-6, HMGB1, and IL-17A differed significantly among the four experimental groups.

**Figure 6:**
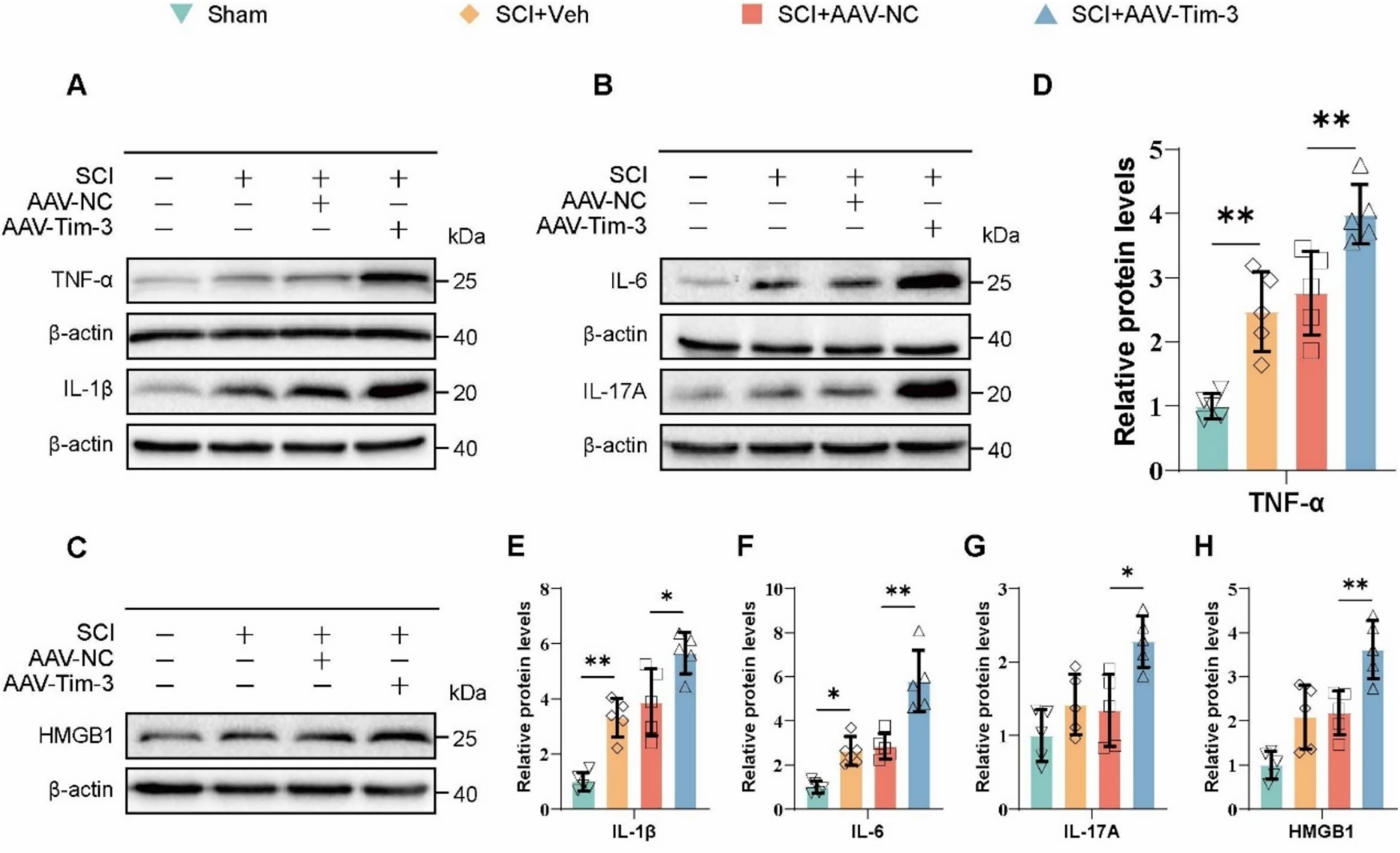
Overexpression of Tim-3 in microglia increased the expression of TNF-α, IL-1β, IL-6, IL-17A, and HMGB1 on day 3 post-SCI. **(A–C)** Representative WB bands for pro-inflammatory factors TNF-α, IL-1β, IL-6, IL-17A, and HMGB1 from spinal cord protein samples. β-Actin serves as the loading control. **(D–H)** Scatter plots presenting the quantitative analysis of TNF-α, IL-1β, IL-6, IL-17A, HMGB1, and Tim-3 expression on day 3 post-SCI (one-way ANOVA followed by Bonferroni post hoc test, *n* = 5 mice per group). All data are presented as mean ± SD. *p < 0.05, **p < 0.01.

We next analyzed the expression of key inflammatory mediators at the peak of Tim-3 expression (3 days post-SCI). WB analysis showed that SCI-induced increases in the pro-inflammatory cytokines TNF-a, IL-1β, and lL-6 were significantly potentiated by Tim-3 overexpression. Furthermore, levels of HMGB1 and lL-17A, additional mediators of inflammation and damage, were also markedly elevated in the AAV-Tim-3 group TNF-α (*F* = 28.346, *p* < 0.01; Figures 6A,D), IL-1β (*F* = 28.061, *p* < 0.05; Figures 6A,E), IL-6 (*F* = 26.751, *p* < 0.001; Figures 6B,F), IL-17A (*F* = 9.081, *p* < 0.05; Figures 6B,G), and HMGB1 (*F* = 17.586, *p* < 0.01; Figures 6C,H).

These results indicate that microglial Tim-3 overexpression exacerbates neuroinflammation by enhancing the release of key pro-inflammatory cytokines, including TNF-α, IL-1β, IL-6, HMGB1, and IL-17A.

### 3.7 Overexpression of Tim-3 in microglia promotes a pro-inflammatory microglial activation state, enhances gliosis, and aggravates neuronal damage

**Figure 7:**
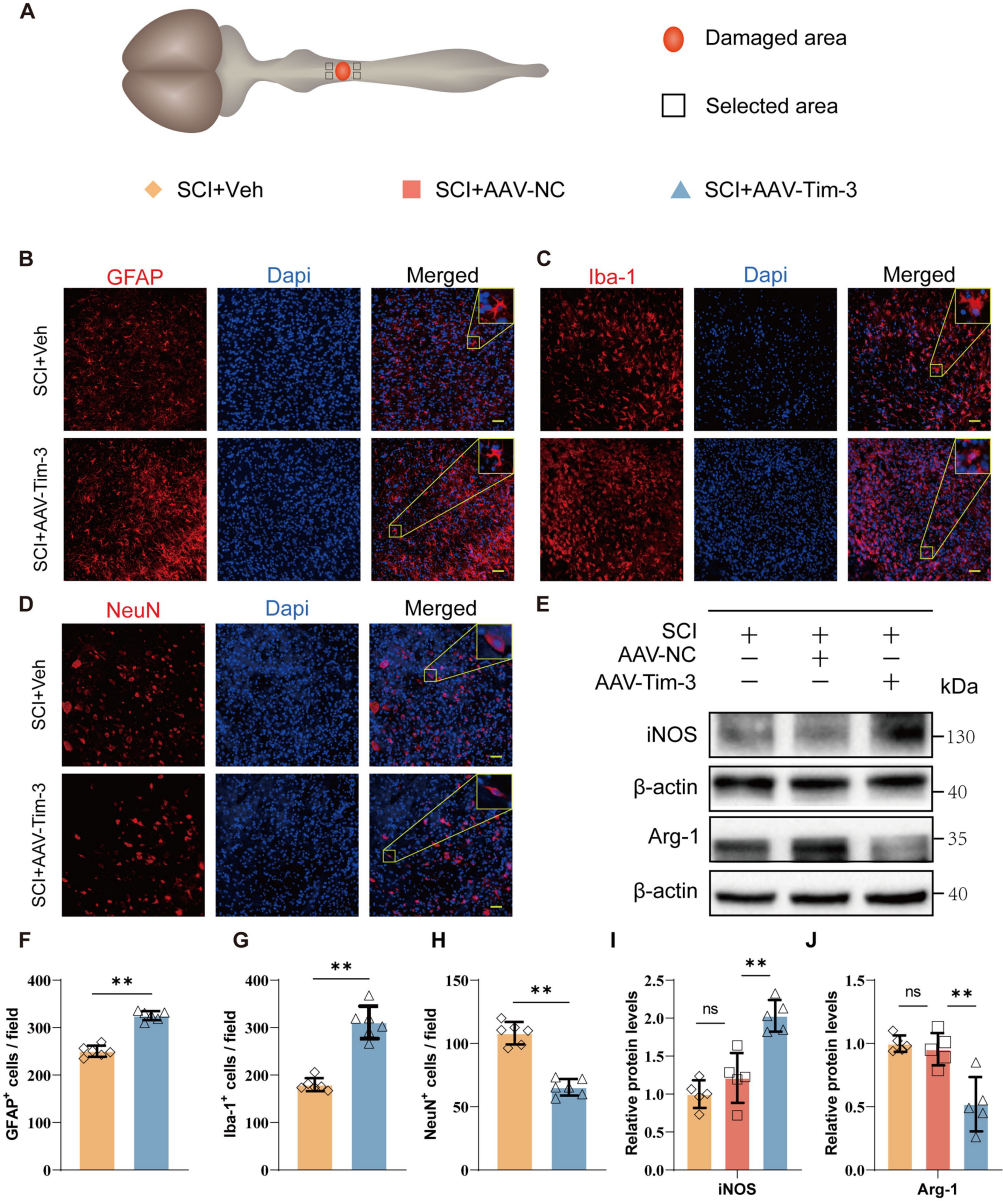
Overexpression of Tim-3 in microglia promotes a pro-inflammatory microglial activation state, enhances gliosis, and aggravates neuronal damage. **(A)** Diagram of selected fields in the three comparison slices for the quantitative analysis of ionized calcium-binding adapter molecule-1 (Iba-1), glial fibrillary acidic protein (GFAP), and neuron-specific nuclear antigen (NeuN) antibody staining. **(B–D)** Representative images of GFAP, Iba-1, and NeuN immunofluorescence staining around the injury site on day 7 post-SCI. The inset shows a high magnification of positive cells. Scale bar = 50 μm. **(F–H)** Scatter plots depicting the quantitative analysis of GFAP, Iba-1, and NeuN positive cells. An independent-samples *t*-test was used to analyze the SCI area, with *n* = 6 mice per group. All data are presented as mean ± SD. **(E)** Representative WB bands of microglial polarization markers iNOS and Arg-1 from spinal cord protein samples. β-Actin serves as the loading control. **(I,J)** Scatter plots presenting the quantitative analysis of iNOS and Arg-1 expression on day 7 post-SCI. The results demonstrated that AAV-Tim-3 significantly upregulated iNOS expression while downregulating Arg-1, indicating a shift towards a predominantly pro-inflammatory activation state (one-way ANOVA followed by Bonferroni post hoc test, *n* = 5 mice per group). All data are presented as mean ± SD. *p < 0.05, **p < 0.01.

To assess cellular responses in the peri-lesion area after SCI (Figure 7A), we first evaluated the immunofluorescence detection of GFAP and Iba-1 at 7 days post-SCI. Immunofluorescence analysis of the lesion epicenter at 7 days post-SCI revealed that Tim-3 overexpression enhanced the activation of both astrocytes (*t* = 12.040, *p* < 0.001; Figures 7B,F) and microglia (*t* = 8.743, *p* < 0.001; Figures 7C,G).

We further investigated the complex activation states of microglia following SCI by analyzing the expression of iNOS and Arg-1. WB analysis revealed that the SCI + AAV-Tim-3 group had significantly elevated iNOS (*F* = 24.030, *p* < 0.001; Figures 7E,I) and reduced Arg-1 levels (*F* = 15.837, *p* < 0.01; Figures 7E,J) compared to the SCI + AAV-NC group. The results demonstrated that AAV-Tim-3 significantly upregulated the pro-inflammatory marker iNOS while downregulating the repair-associated marker Arg-1, suggesting a shift towards a predominantly pro-inflammatory activation state.

Neuronal survival was evaluated by NeuN immunofluorescence. The SCI + AAV-Tim-3 group exhibited significantly fewer NeuN-positive neurons than the SCI + Veh group (*t* = −9.467, *p* < 0.001; Figures 7D,H), demonstrating that microglial Tim-3 overexpression impairs neuronal survival in the acute phase after SCI.

### 3.8 The detrimental effects of microglial Tim-3 are mediated via the Nrf2/HMGB1 axis and are reversed by Nrf2 activation

**Figure 8:**
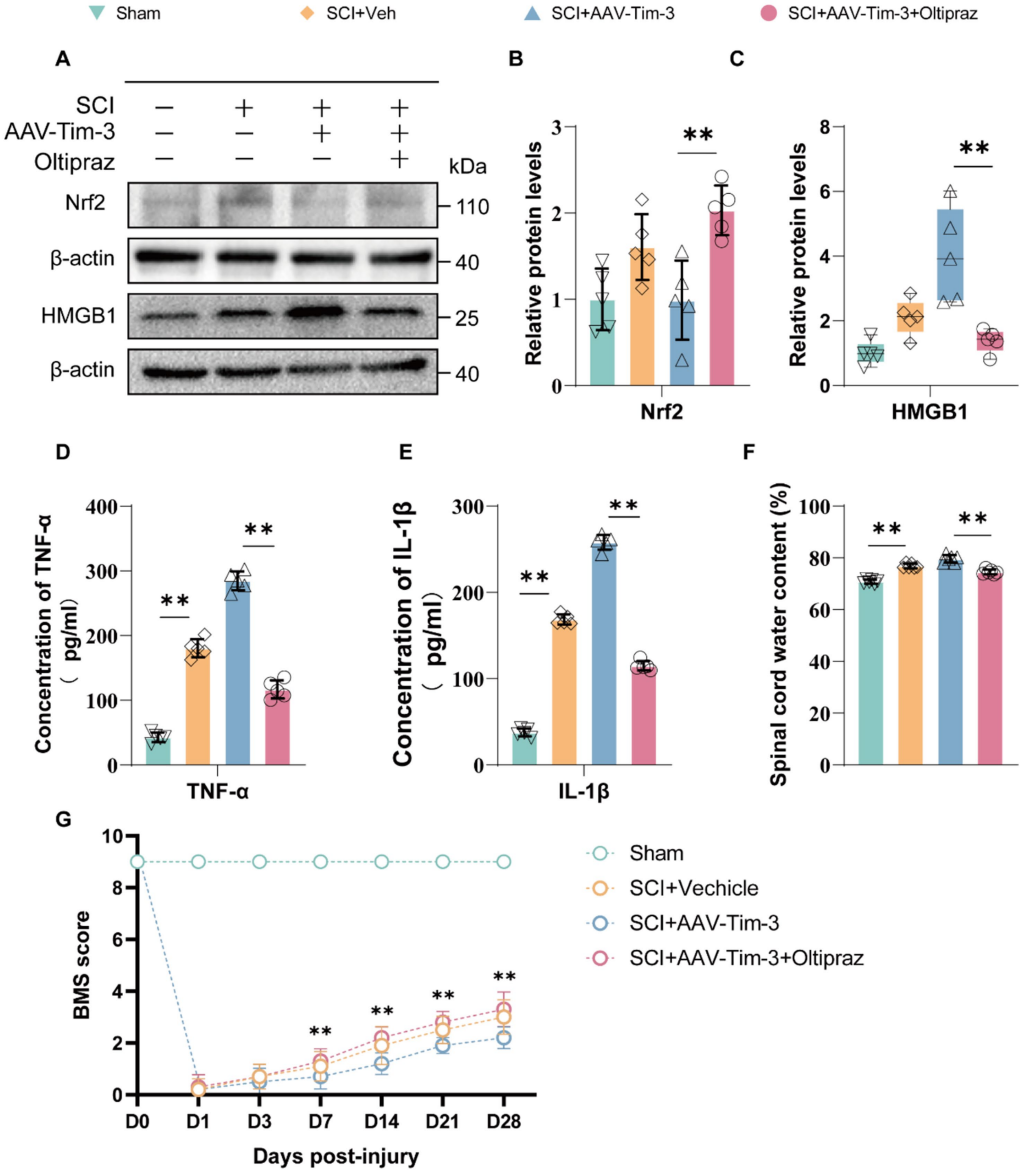
Microglial Tim-3 overexpression exerts its effects through the Nrf2/HMGB1 signaling pathway. **(A)** Representative WB bands for Nrf2 and HMGB1 from spinal cord protein samples. β-Actin serves as the loading control. **(B,C)** Scatter plots presenting the quantitative analysis of Nrf2 (one-way ANOVA followed by Bonferroni *post hoc* test, *n* = 5 mice per group) and HMGB1 (the Kruskal–Wallis test was performed, *n* = 5 mice per group) expression on day 3 post-SCI. **(D,E)** Oltipraz treatment reduced the expression of the pro-inflammatory cytokines TNF-α, IL-1β on day 3 post-SCI (one-way ANOVA followed by Bonferroni *post hoc* test, *n* = 5 mice per group). **(F)** On day 3 post-SCI, Oltipraz reduced the increase of water content in injured spinal cord caused by the over-expression of Tim-3 in microglia. One-way ANOVA followed by Bonferroni *post hoc* test is used to analyze spinal cord water content. *n* = 6 mice per group. **(G)** BMS scores are used to evaluate the recovery of motor function in all four groups. GEE are used for analysis. *n* = 10 mice per group. All data are presented as mean ± SD. *p < 0.05, **p < 0.01.

To investigate whether microglial Tim-3 overexpression promotes neuroinflammation by suppressing Nrf2 and enhancing HMGB1 expression, we analyzed protein levels of Nrf2 and HMGB1 by western blot and administered the Nrf2 agonist Oltipraz. Tim-3 overexpression significantly reduced Nrf2 expression while upregulating HMGB1 (Nrf2: *F* = 9.059, *p* < 0.01, Figures 8A,B; HMGB1: *p* < 0.01, Figures 8A,C). Pharmacological activation of Nrf2 with Oltipraz effectively reversed these alterations and reduced pro-inflammatory cytokine levels (TNF-α: *F* = 316.666, *p* < 0.001, Figure 8D; IL-1β: *F* = 1083.067, *p* < 0.001, Figure 8E). Moreover, Oltipraz treatment significantly attenuated spinal cord edema (*F* = 84.845, *p* < 0.001, Figure 8F) and improved motor recovery from day 7 to 28 post-SCI (Wald *χ*^2^ = 21.832, *p* < 0.001; day 7: *p* < 0.01; days 14–28: *p* < 0.001, Figure 8G). These results suggest that microglial Tim-3 overexpression aggravates SCI at least partially through the Nrf2/HMGB1 signaling pathway.

## 4 Discussion

This study reveals the underlying mechanism by which microglial Tim-3 induction exacerbates the inflammatory response in SCI, which involves the regulation of the Nrf2/HMGB1 signaling axis. Originally identified as an immune checkpoint molecule, Tim-3 is predominantly expressed on Th1 cells but absent in Th2 cells, with additional presence in monocytes/macrophages, mast cells, and dendritic cells. With the in-depth study of the CNS, it was found that Tim-3 could be expressed in microglia in large quantities, but there were different opinions on whether and to what extent Tim-3 was expressed in neurons and astrocytes. In fact, many inflammatory diseases suggest that Tim-3 plays different roles in different diseases, and the difference may be related to its tissue structure and pathogenesis. In immune-driven pathologies, Tim-3 typically suppresses immunocyte activity. Its primary ligand, galectin-9, induces Th1 cell apoptosis and modulates Treg subset ratios upon binding (33). In a study of patients from multiple sclerosis (MS), it was found that the level of Tim-3 in CD4 cells of MS patients was lower than that of healthy controls, and the production of INF-γ inflammatory factors increased. Blocking Tim-3 would lead to the increase of IL-12 inflammatory factors mediated by TLR in human peripheral blood mononuclear cells (34).

Emerging evidence highlights Tim-3 as a critical mediator of neuroinflammation in brain injury. In ischemic stroke patients, Tim-3^+^ CD14^+^ monocyte frequency shows a significant positive correlation with plasma TNF-α levels (35). Similarly, intracerebral hemorrhage studies identify Tim-3 as a proinflammatory effector, amplifying local TNF-α, IL-1β production and exacerbating cerebral edema. Mechanistically, hypoxia induces Tim-3 via hypoxia inducible factor 1 subunit alpha (HIF-1α)-dependent regulation, and Tim-3 blockade reduces infarct volume, neuronal death, edema, and neutrophil infiltration in murine ischemic models (36).

Further investigations revealed that Tim-3 overexpression induces TLR4-Galectin-9 dissociation, leading to TLR4 exposure. This exposed TLR4 conformation potentiates enhanced pro-inflammatory cytokine release (37). Notably, among CNS cell types, microglia exhibit the highest Tim-3 expression compared to neurons and astrocytes. However, the precise mechanistic role of microglial Tim-3 in SCI remains unexplored. Based on the observations above, we hypothesize that, similar to its role in other sterile inflammatory responses, Tim-3 exerts a pro-inflammatory effect in SCI, primarily mediated through microglia. Following SCI, microglia are recruited to the lesion site and upregulate the Tim-3 receptor. This leads to the promotion of microglial phenotypic shift and the release of microglia-associated inflammatory factors via Tim-3 receptor-related signaling axis, thereby exacerbating SCI.

In this study, microglia-specific Tim-3 overexpression was achieved via AAV-mediated gene delivery, revealing its proinflammatory role in SCI pathophysiology. Under SCI conditions, Tim-3 promoted a pro-inflammatory activation state of microglia characterized by elevated iNOS expression and robust production of pro-inflammatory cytokines, while also stimulating the secretion of neurotoxic mediators, thereby exacerbating secondary SCI. The increased expression of Th17-related inflammatory factors suggests that the pro-inflammatory effect of microglial Tim-3 may be associated with peripheral immunity. Mechanistic investigations further revealed that these effects are partially mediated by dysregulation of the Nrf2/HMGB1 signaling axis. Administration of the Nrf2 agonist Oltipraz reduced the release of microglia-associated inflammatory factors and improved neurological outcomes.

Following SCI, microglia transition from a resting state to a predominantly pro-inflammatory activation state (characterized by high iNOS expression), with only minimal manifestation of a repair-associated state (marked by Arg-1 and CD206) (38). Microglia in this pro-inflammatory activation state secrete classical inflammatory cytokines, including TNF-α, IL-1β, and IL-6, which are robustly induced after CNS trauma.

HMGB1 is a non-histone chromosome binding protein widely found in eukaryotic nuclei. When cells are damaged or necrotic, HMGB1 can be used as damage associated molecular pattern (DAMP) to activate immune response, which is closely related to inflammation (39). In the injured CNS, HMGB1 drives microglial polarization toward the M1 phenotype and exacerbates neuroinflammation via TLR/RAGE signaling pathways, ultimately promoting neuronal apoptosis (40). Notably, HMGB1 levels correlate positively with the extent of neuronal damage (41). Post-SCI, HMGB1 is released by neurons and microglia, and exogenous HMGB1 treatment upregulates microglial RAGE, TNF-α, IL-1β, and IL-17 expression, whereas HMGB1 inhibition produces the opposite effect (42).

Tim-3 induces Th1 cell death, thereby relieving Th1-mediated inhibition of Th17 cells and increasing IL-17A, while microglia recruit these cells to the injury site (43, 44). Our experiments show that microglial Tim-3 overexpression promotes IL-17A elevation at the injury site, leading us to hypothesize that following SCI, microglial Tim-3 overexpression recruits T cells to the lesion and promotes the release of IL-17A and other inflammatory factors, thereby exacerbating local inflammation.

Nrf2, a nuclear transcription factor, plays a pivotal role in regulating cellular inflammatory responses, oxidative stress, and apoptosis (45). When the CNS is injured, it is often accompanied by the decrease of Nrf2 expression and the increase of microglia activation. By activating Nrf2 pathway, M1 microglia can be inhibited and the transformation from microglia to M2 phenotype can be promoted (46). One study showed that elevated Tim-3 signaling may exacerbate Nrf2 degradation through increased ubiquitination (47). Nrf2 agonist treatment not only reversed these Tim-3-mediated proinflammatory effects but also attenuated spinal cord lesion area. These findings collectively suggest that microglial Tim-3 exacerbates SCI pathogenesis through a mechanistic cascade involving Nrf2 suppression and subsequent HMGB1 upregulation. Our findings demonstrate that microglial Tim-3 overexpression upregulates HMGB1 while downregulating Nrf2 expression. Pharmacological Nrf2 activation reversed these effects, concomitant with reductions in pro-inflammatory cytokines (TNF-α, IL-1β), attenuated tissue edema, and improved locomotor recovery.

In summary, this study has achieved significant methodological and mechanistic progress in the context of SCI. At the methodological level, unlike conventional studies employing ubiquitous AAV vectors for non-specific cellular intervention, this study innovatively introduced a novel AAV-MG1.2 vector driven by the CD68 promoter. This refinement confirmed the specific role of microglia, demonstrating that targeted modulation of Tim-3 expression exclusively in this cell population is sufficient to drive substantial secondary injury. Regarding mechanistic elucidation, this study not only revealed the dynamic polarization process through which Tim-3 drives microglial conversion toward a pro-inflammatory phenotype (characterized by increased iNOS expression and decreased Arg-1 expression) but also observed a concomitant elevation in the downstream inflammatory cytokine IL-17A. This finding provides potential clues for future investigations into whether microglial Tim-3 may mediate central-peripheral immune crosstalk. Collectively, this study deepens our understanding of the pro-inflammatory effects of microglial Tim-3 in traumatic CNS disorders and provides robust experimental evidence supporting the future development of highly precise cell-targeted therapeutic strategies.

This study also has several limitations. First, experimental investigation of the Nrf2 signaling axis has certain constraints, and further validation using Nrf2 knockdown or knockout approaches is required. In animal experiments, the specific mechanisms by which microglia exert their effects are influenced by various cellular or environmental factors, and this study has limitations in verifying the role of Nrf2 in the mechanism underlying Tim-3 function. Second, Tim-3 is also an important peripheral immunity-related molecule, but this study did not thoroughly explore the interaction between central nervous system inflammatory responses and peripheral immune responses following SCI. Furthermore, this study primarily examined a limited number of markers, which can only partially reflect the complex state of microglia. Third, although our experiments demonstrated that Oltipraz counteracts the detrimental effects of microglial Tim-3, the Nrf2 agonist activity induced by this administration regimen persisted only until the first day post-SCI. The complex changes in Nrf2 expression following drug discontinuation were not considered, which reduces the rigor of this experimental design. Fourth, the absence of a negative control virus in some experiments diminishes the persuasiveness of the corresponding results. Finally, the potential influences of sex and age on microglial responses were not fully considered, representing important directions for future investigation.

## 5 Conclusion

In conclusion, this study demonstrates that microglial Tim-3 exacerbates spinal cord injury by promoting a pro-inflammatory activation state of microglia and aggravating neuroinflammation, primarily through the dysregulation of the Nrf2/HMGB1 signaling axis. Our findings reveal a novel mechanism in SCI pathogenesis and highlight microglial Tim-3 as a promising therapeutic target.

## Data Availability

The raw data supporting the conclusions of this article will be made available by the authors, without undue reservation.

## Glossary

CNS: Central nervous system
Tim-3: T-cell immunoglobulin and mucin domain-containing molecule 3
SCI: Spinal cord injury
BMS: The Basso Mouse Scale
SPT: Sucrose preference test
ELISA: Enzyme-linked immunosorbent assay
WB: Western blot
TNF-α: Tumor necrosis factor-alpha
IL-1β: Interleukin-1beta
Nrf2: Nuclear factor erythrocyte 2–associated factor 2
ROS: Reactive oxygen species
SOD: Superoxide dismutase
CAT: Catalase
HO-1: Heme oxygenase-1
AAV: Adeno-associated virus
SPF: Specific pathogen-free
PBS: Phosphate-buffered saline
PFA: Paraformaldehyde
LFB: Luxol Fast Blue
WW: Wet weight
DW: Dry weight
IQR: Interquartile range
GEE: Generalized estimating equations
DAMP: Damage associated molecular pattern
APCs: Antigen-presenting cells
