# Supplementary material for: The role of microglial Tim-3 in neuroinflammation and functional recovery after spinal cord injury
Source: Front Neurol. 2026 Jun 19;17:1828458. doi: 10.3389/fneur.2026.1828458 (PMC13327981; doi:10.3389/fneur.2026.1828458)
Supplement: Supplementary file 1 [file Data_Sheet_1.PDF]

Western bloting-Arg-1

1：SCI+Veh

2：SCI+AAV-NC

3：SCI+AAV-Tim-3

Group 1.

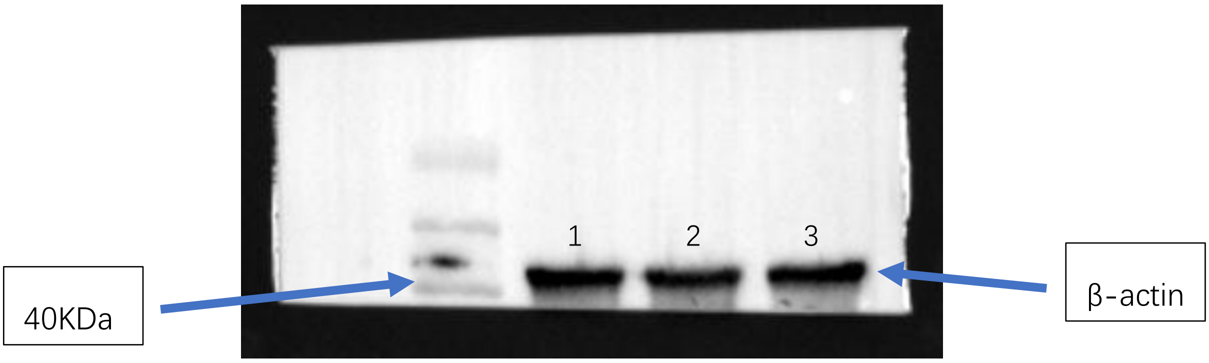

(β-actin KDa)

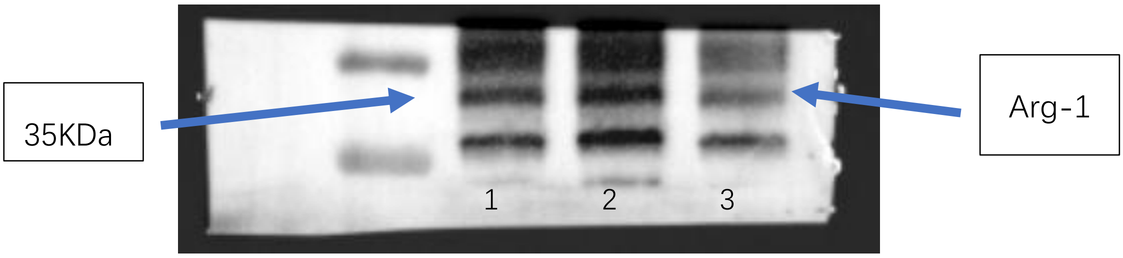

(Arg-1 KDa)

Arg-1

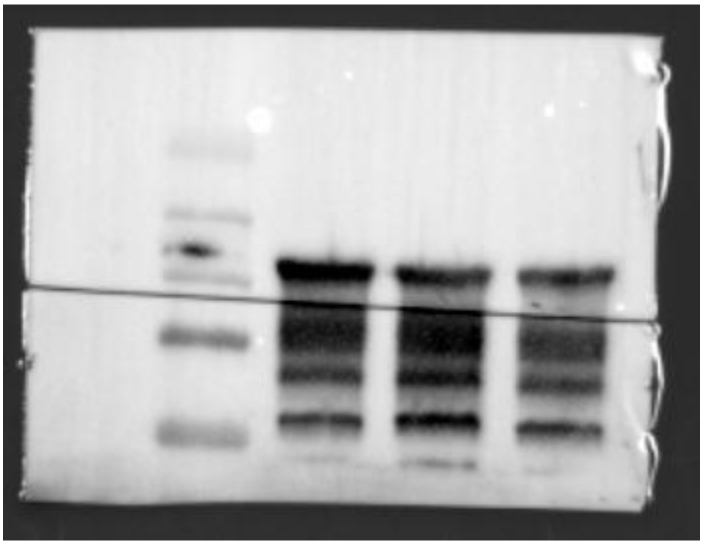

(Merged)

Group 2.

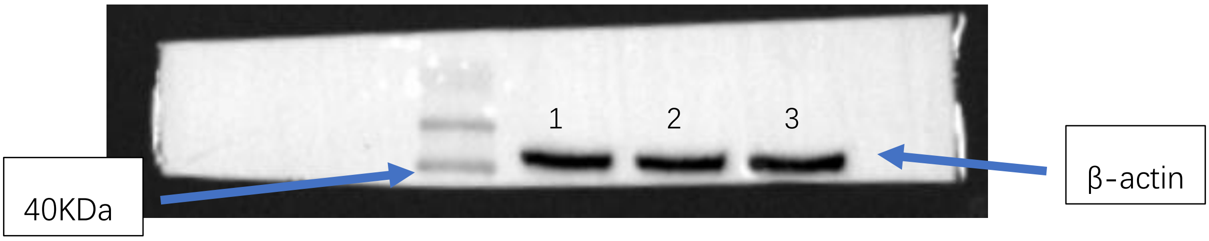

(β-actin KDa)

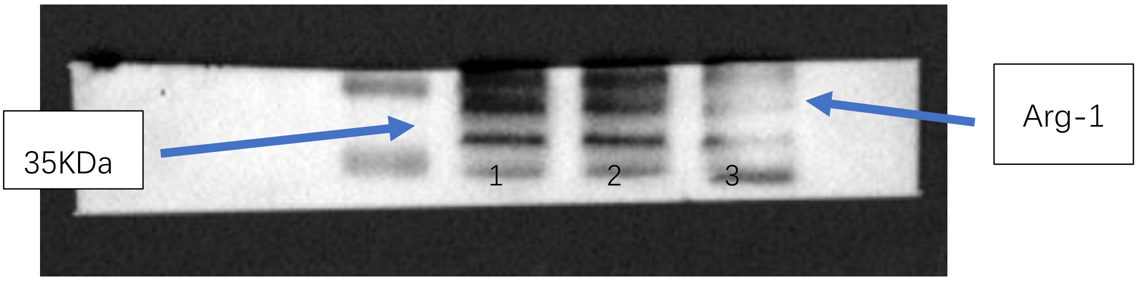

(Arg-1KDa)

Arg-1

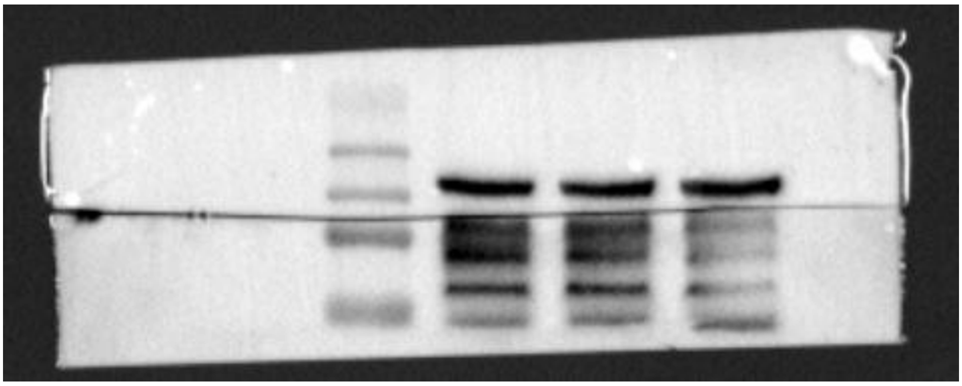

(Merged)

Group 3.

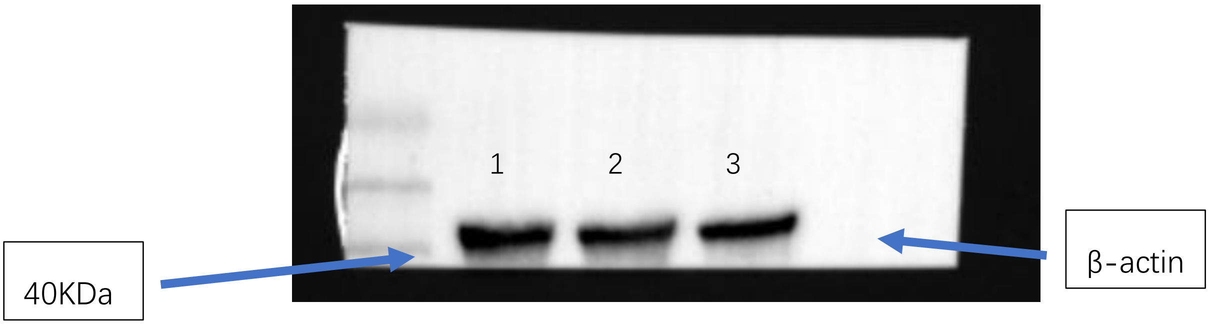

(β-actin KDa)

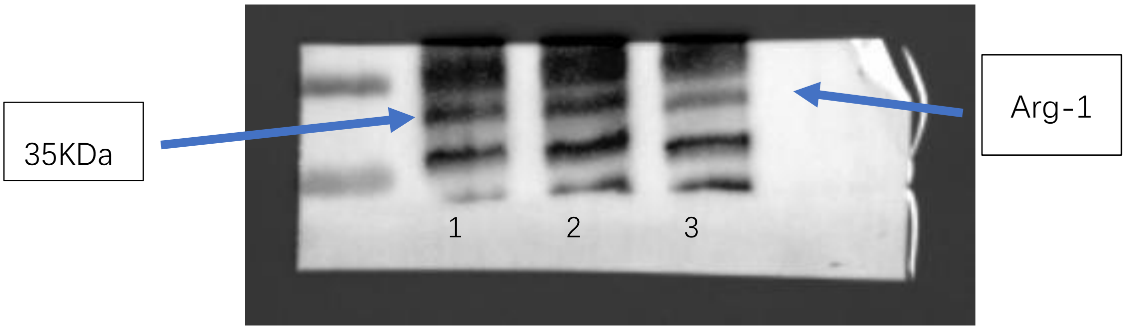

(Arg-1KDa)

Arg-1

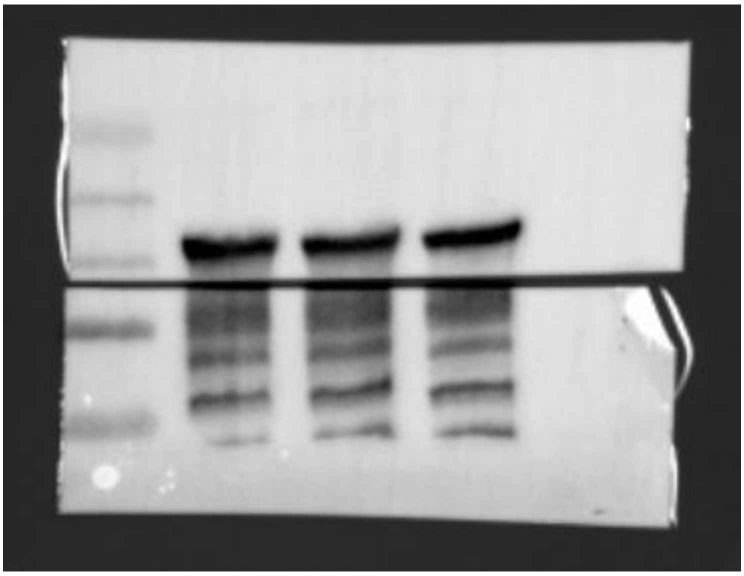

(Merged)

Group4.

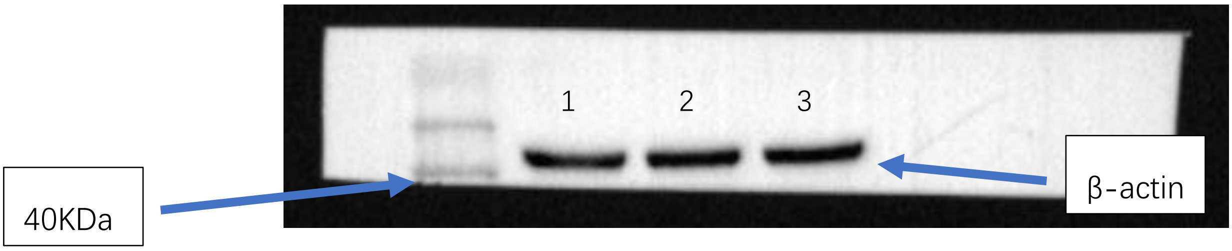

(β-actin KDa) **Figure 7E**

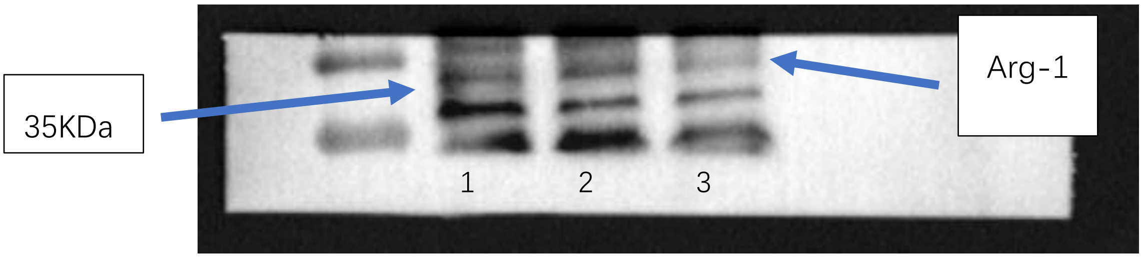

(Arg-1KDa) **Figure 7E**

Arg-1

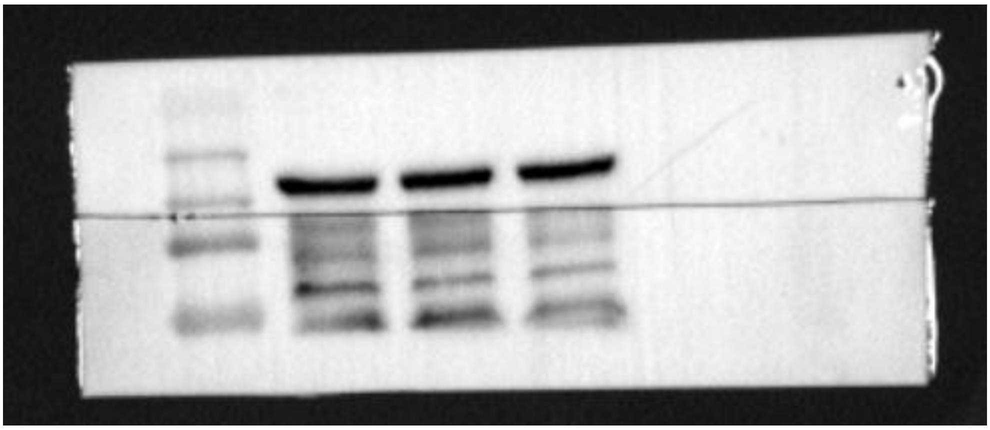

(Merged)

Group 5.

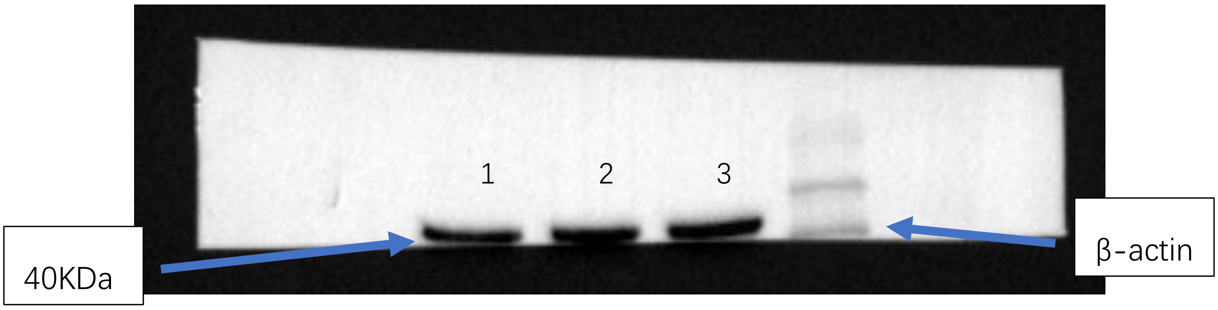

(β-actin KDa)

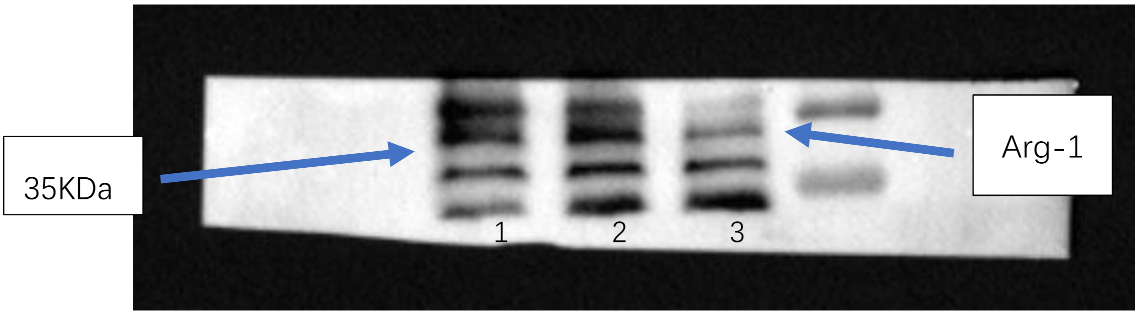

(Arg-1KDa)

Arg-1

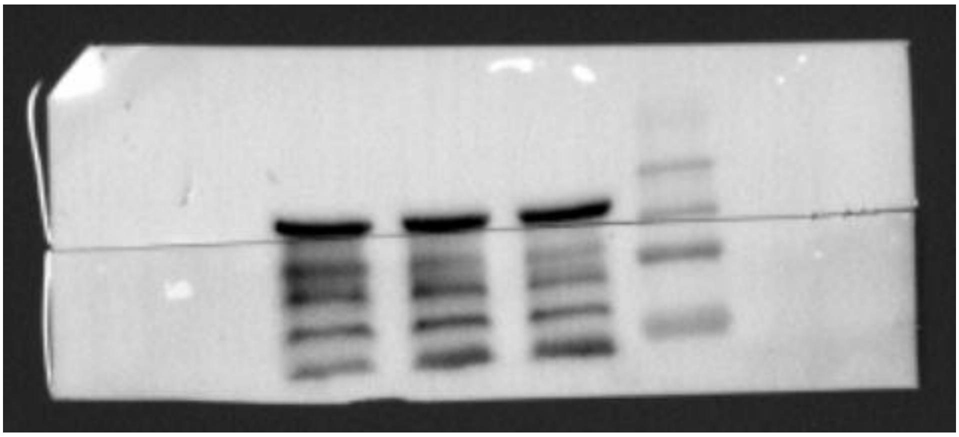

（Merged）

eGFP

Western bloting-eGFP

1：SCI+Veh

2：SCI+AAV-NC

3：SCI+AAV-Tim-3

Group 1.

1 2 3

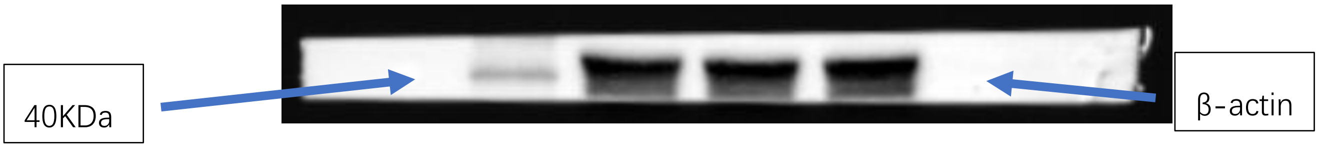

(β-actin kDa)

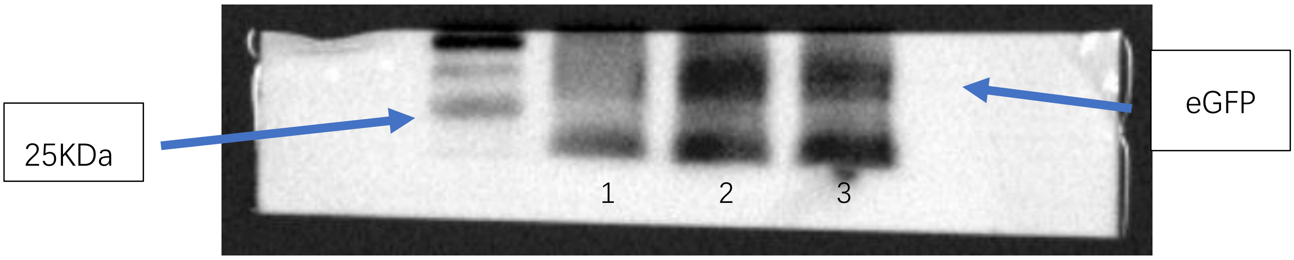

(eGFP kDa)

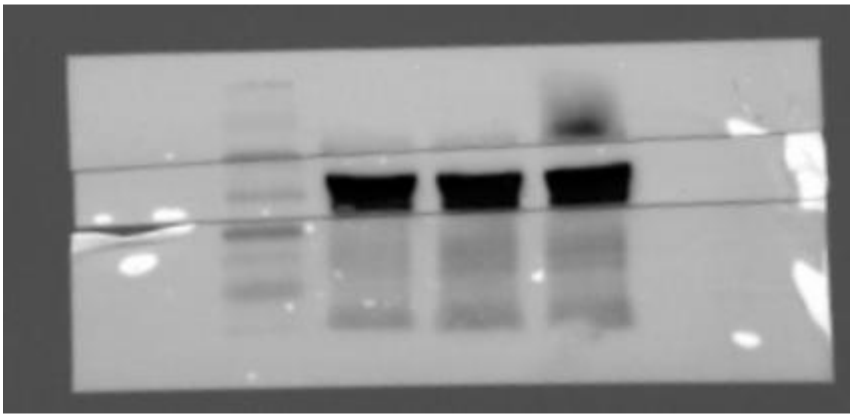

(Figure 2C)

Group 2

1 2 3

eGFP

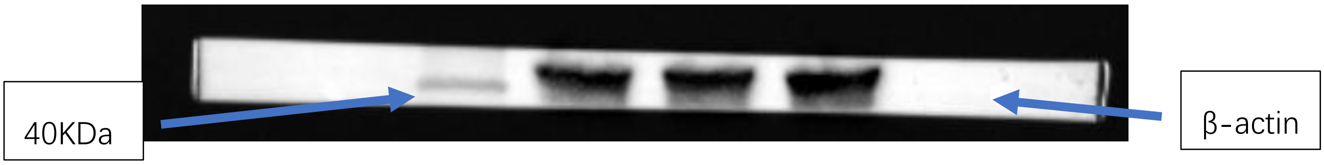

(β-actin kDa)

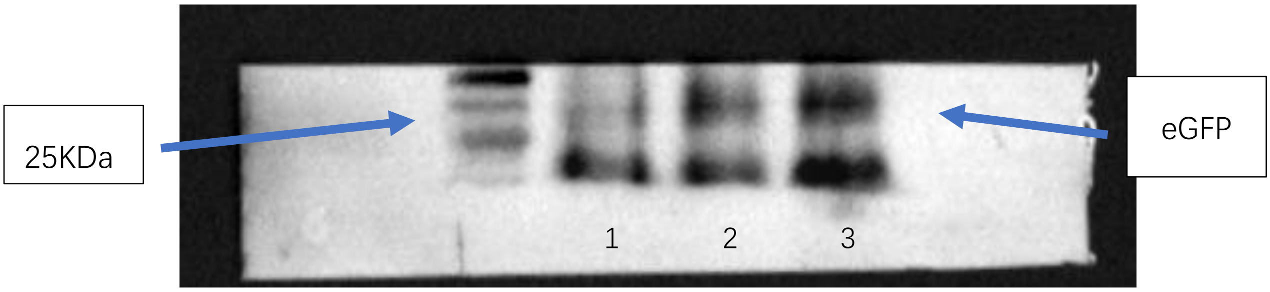

(eGFP kDa)

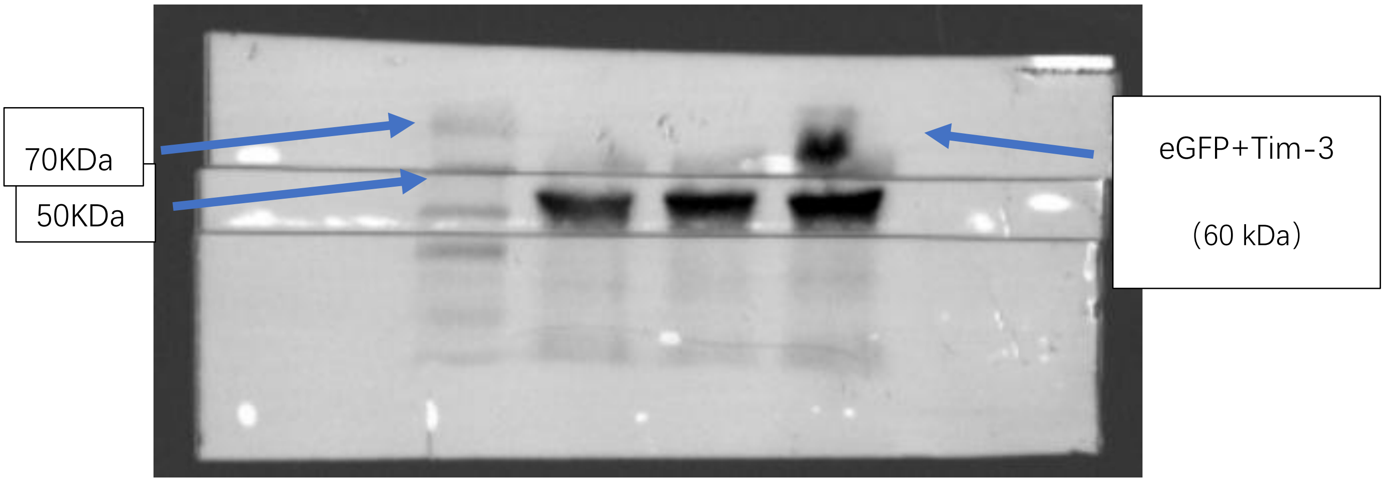

Validation experiment: Through inquiries to Genchem Company, it was confirmed that the band at the molecular weight of approximately 60 kDa corresponds to the viral editing protein, where eGFP+Tim-3 was not cleaved. Since this is a validation experiment and the group without virus injection (SCI+Veh) showed no eGFP protein expression, the specific relative expression level of eGFP was not quantified.

Western bloting-HMGB1

1：Sham

2：SCI+Veh

3：SCI+AAV-NC

4：SCI+AAV-Tim-3

Group 1.

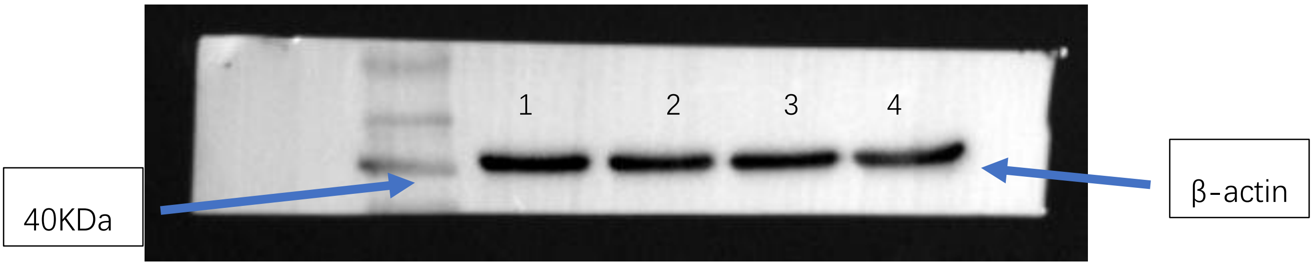

（β-actin KDa）

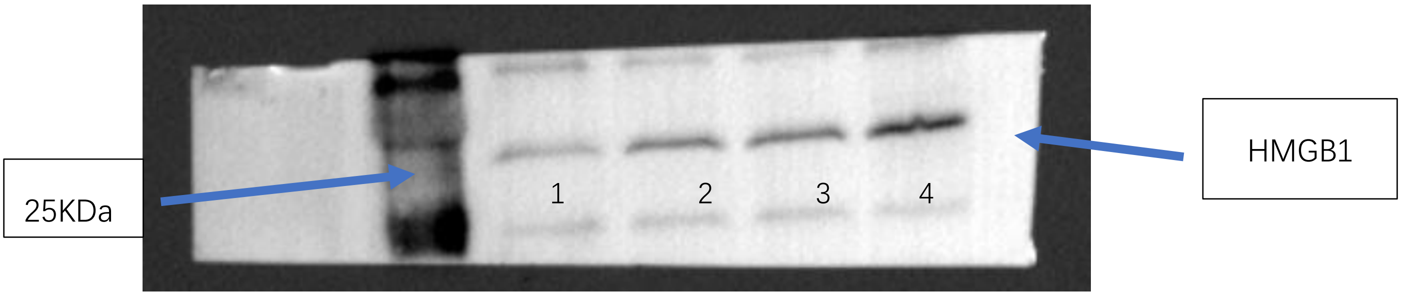

（HMGB1 KDa）

HMGB1

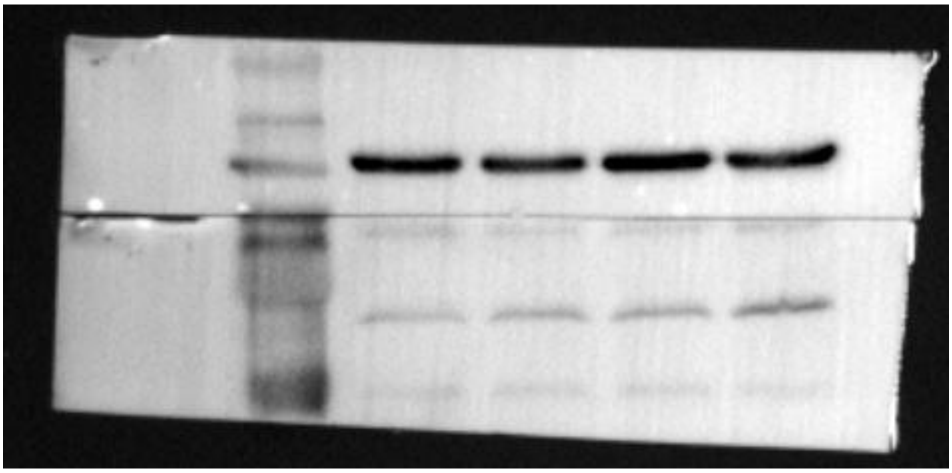

(Merged)

Group 2.

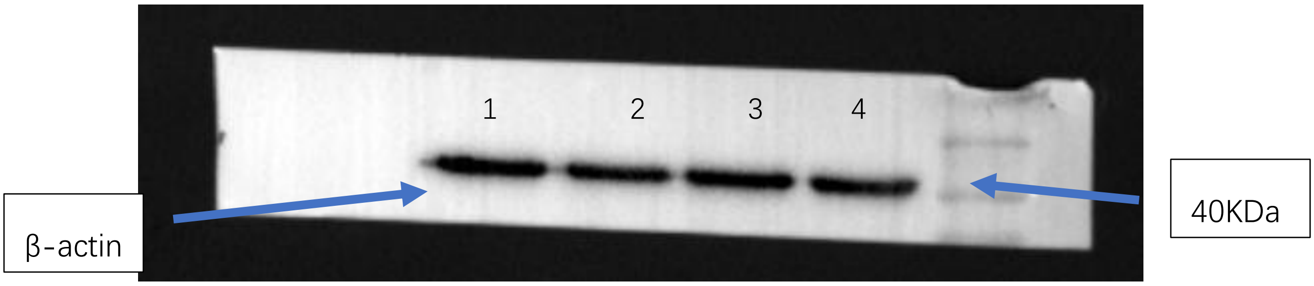

(β-actin KDa) **Figure 6C**

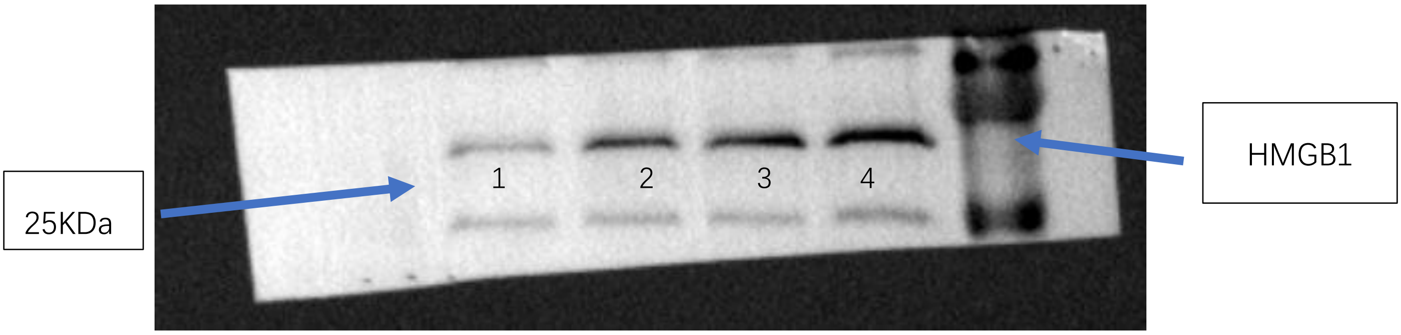

(HMGB1KDa) **Figure 6C**

HMGB1

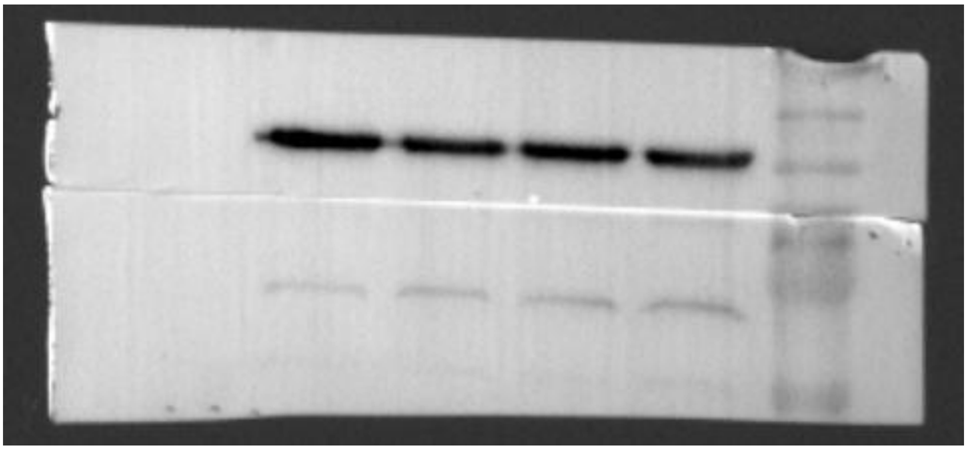

(Merged)

Group 3.

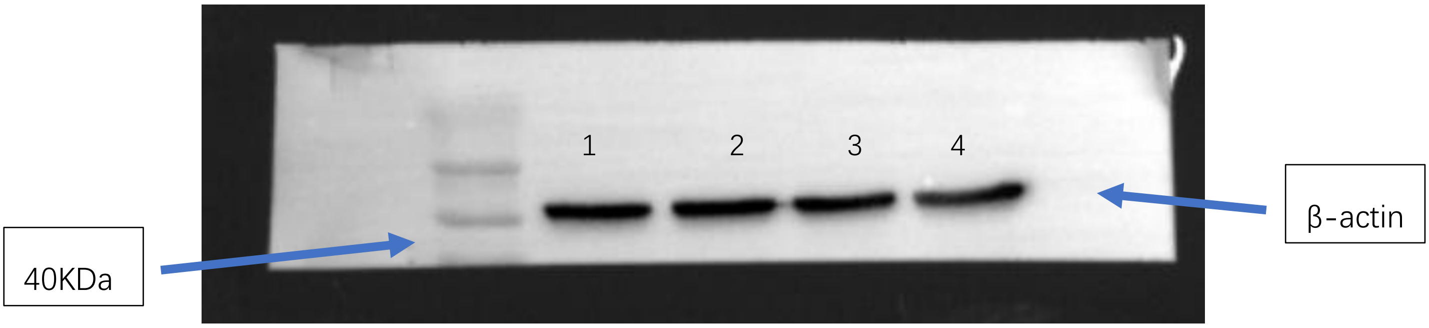

(β-actin KDa)

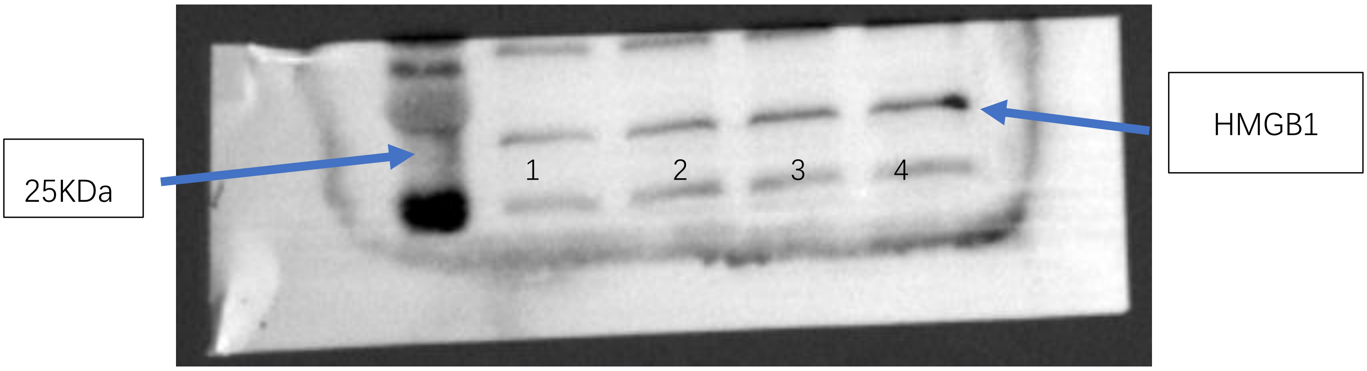

(HMGB1KDa)

HMGB1

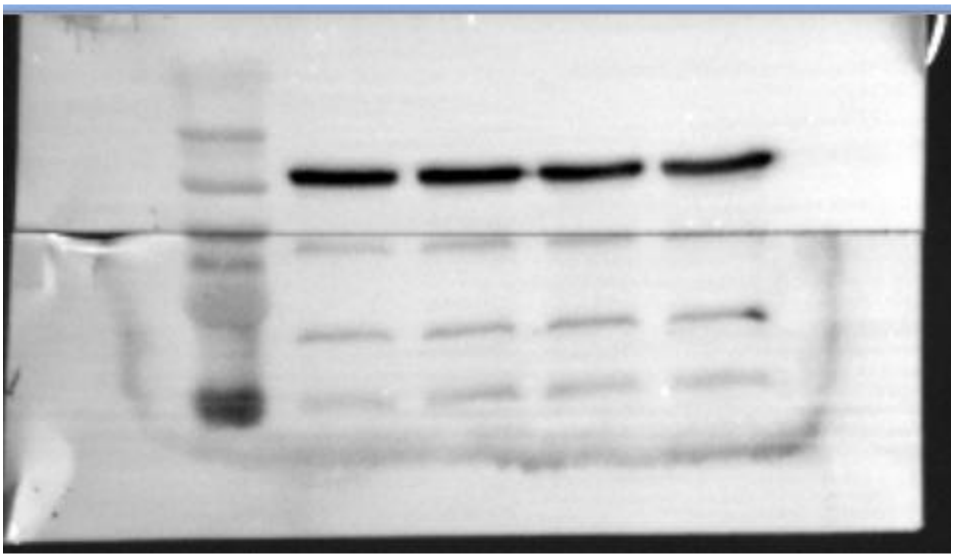

(Merged)

Group4.

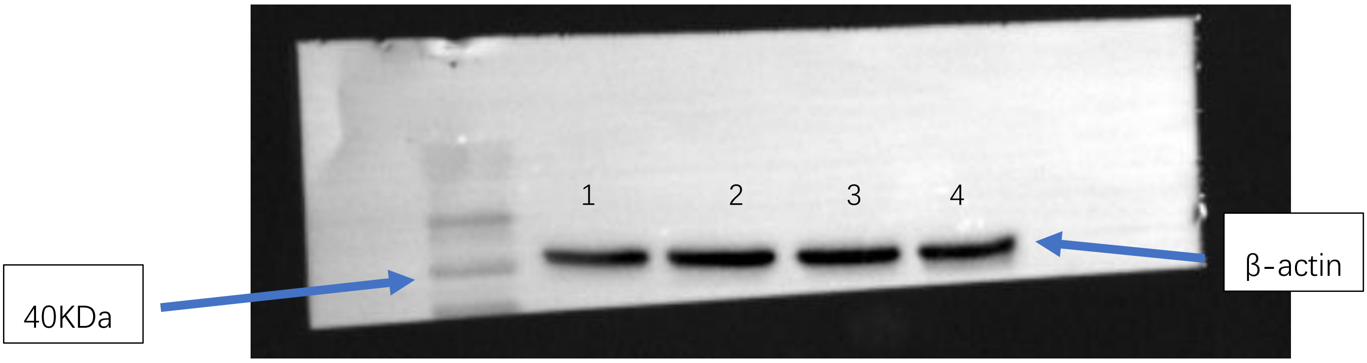

(β-actin KDa)

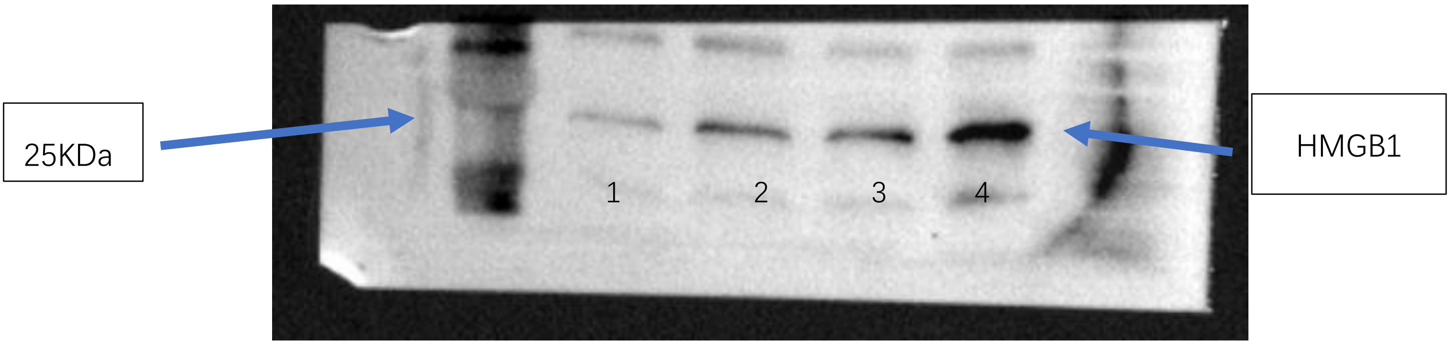

(HMGB1KDa)

HMGB1

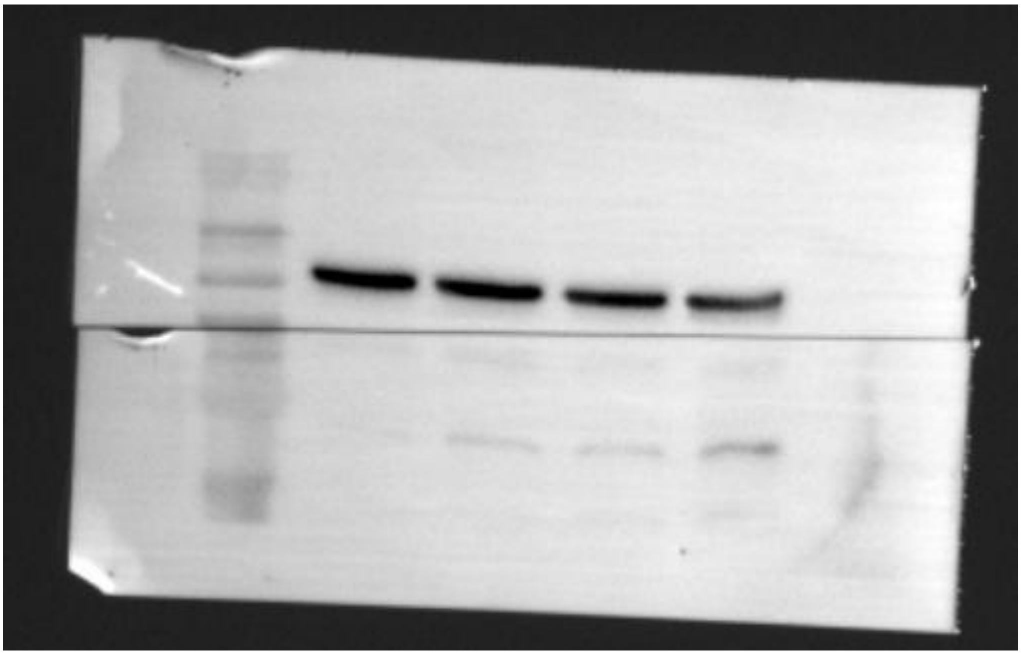

（Merged）

Group 5.

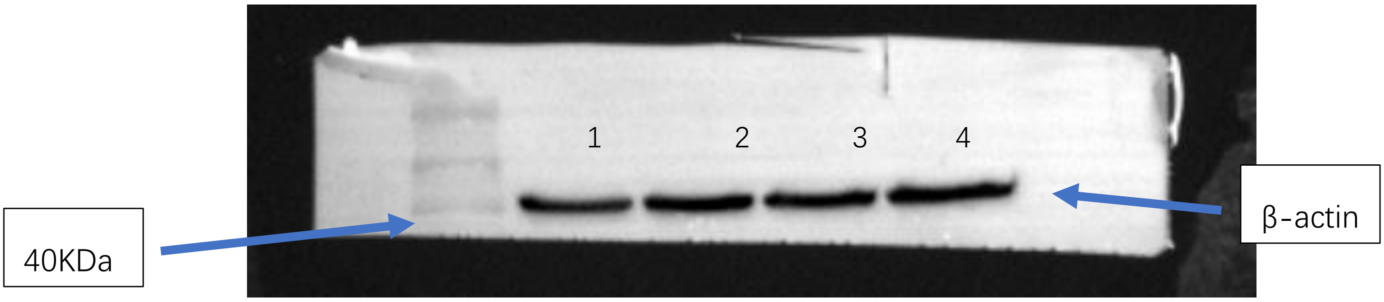

（β-actin KDa）

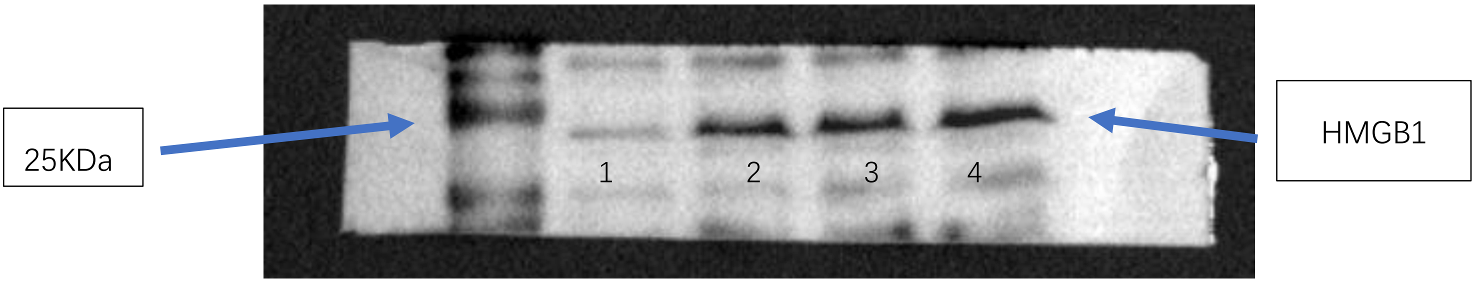

（HMGB1KDa）

HMGB1

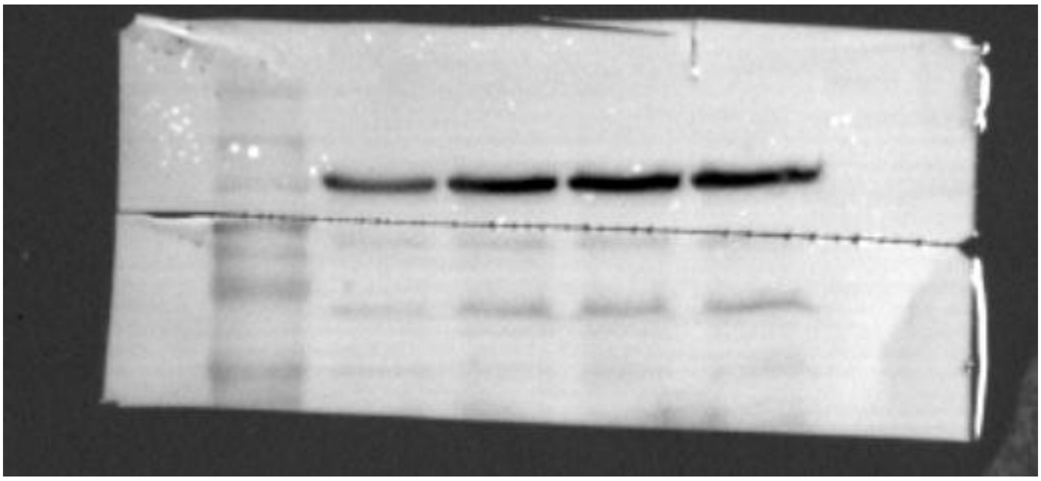

（Merged）

Western bloting-IL-1β

1：Sham

2：SCI+Veh

3：SCI+AAV-NC

4：SCI+AAV-Tim-3

Group 1.

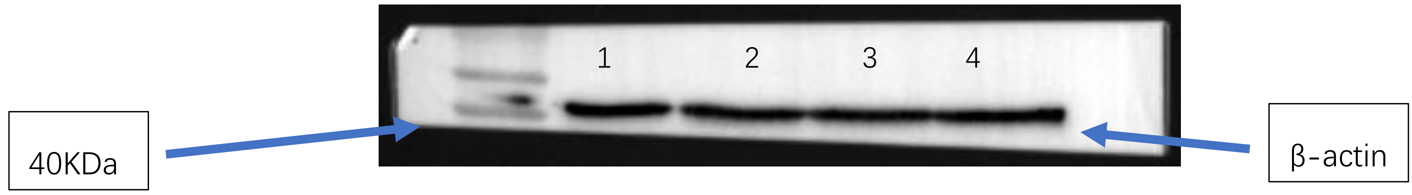

(β-actin KDa)

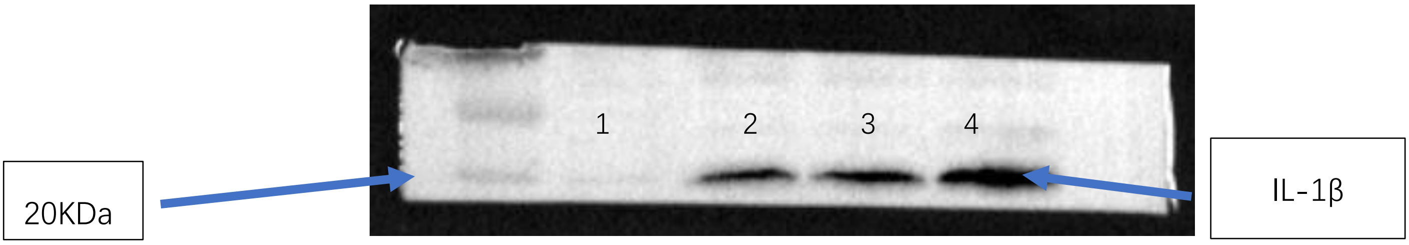

(IL-1β KDa)

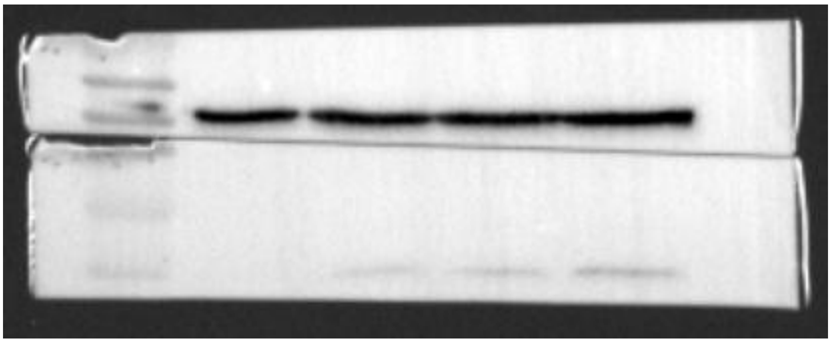

(Merged)

Group 2.

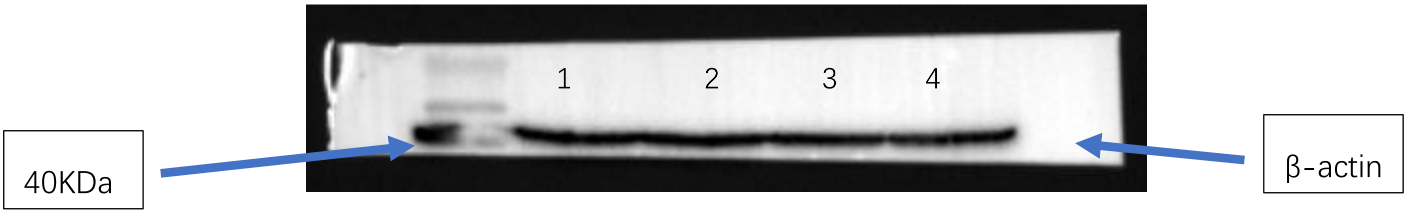

(β-actin KDa)

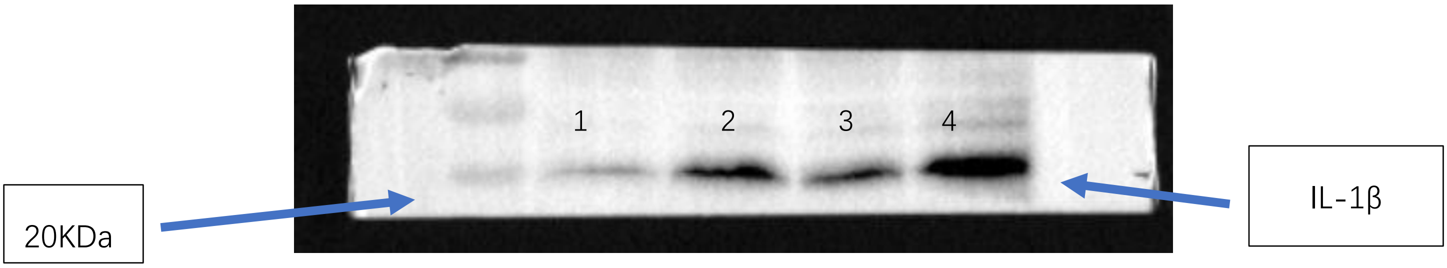

(IL-1β KDa)

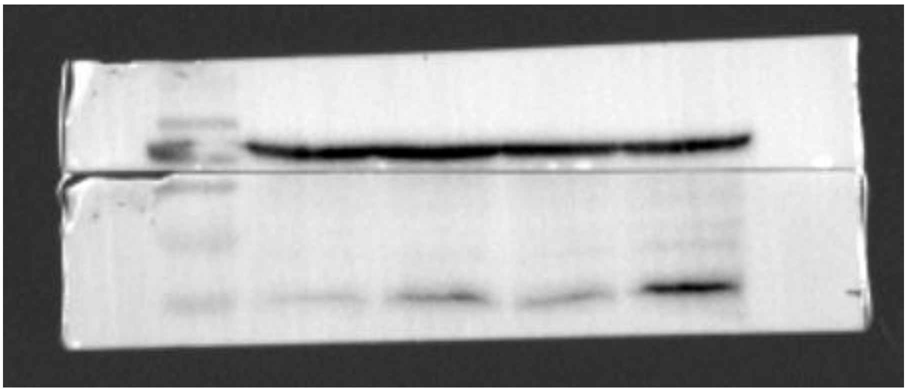

(Merged)

Group 3.

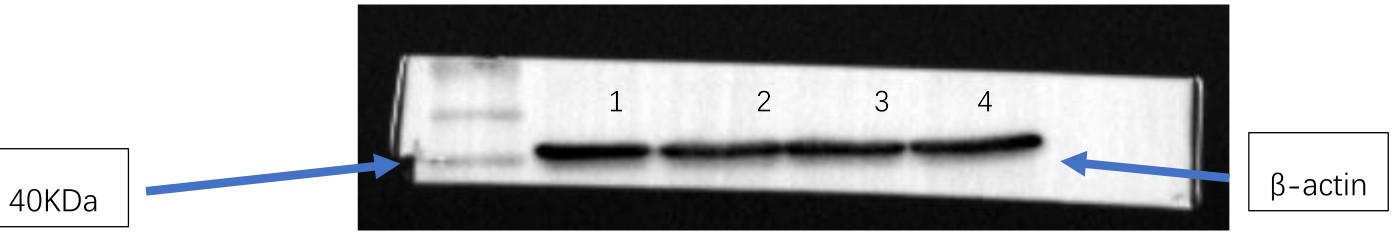

(β-actin KDa)

IL-1β

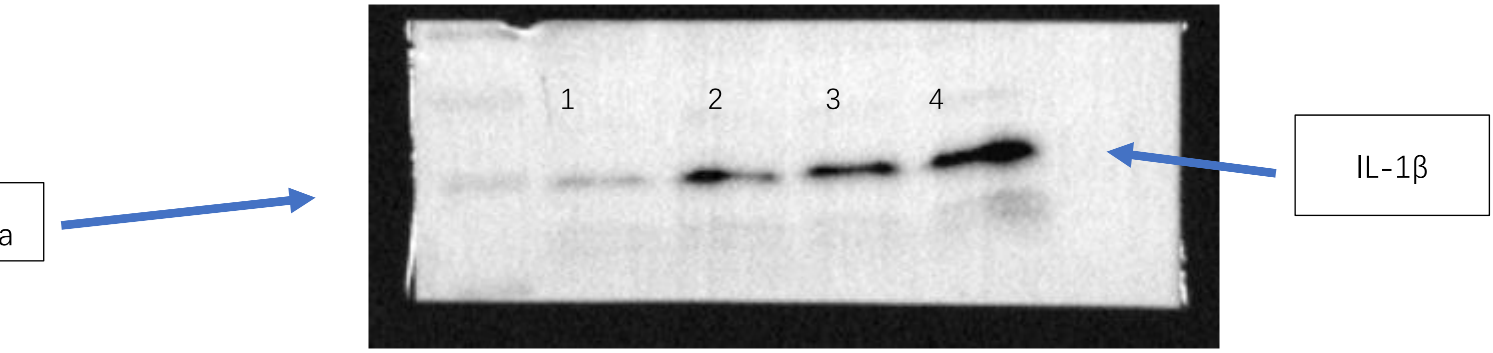

(IL-1β KDa)

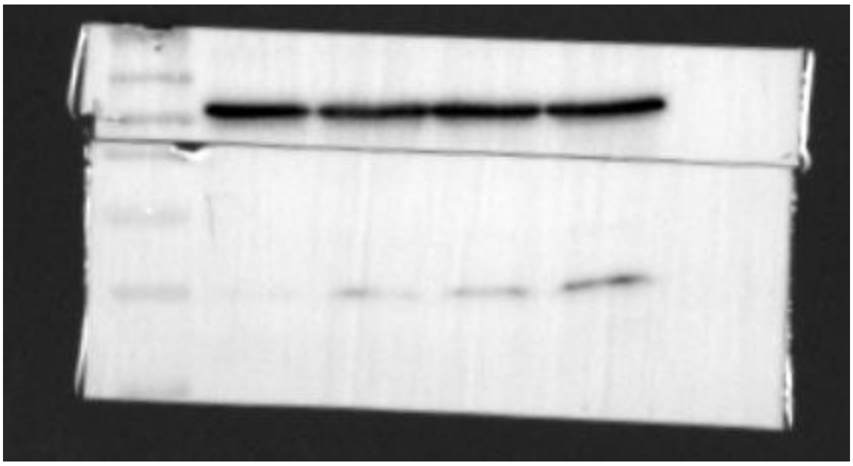

(Merged)

Group 4.

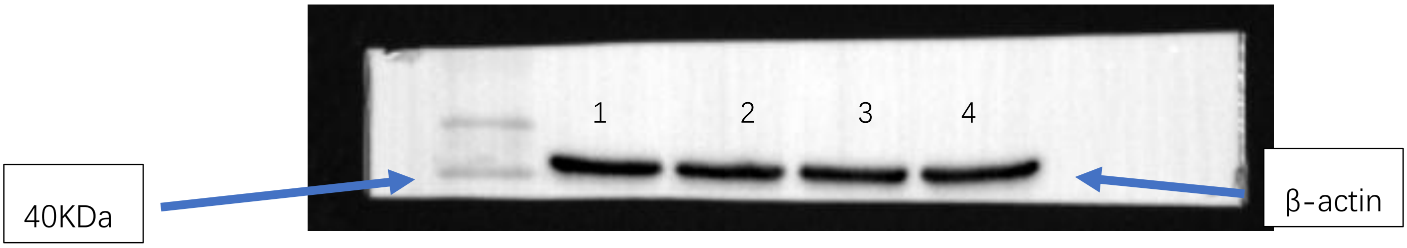

(β-actin KDa) **Figure 6C**

IL-1β

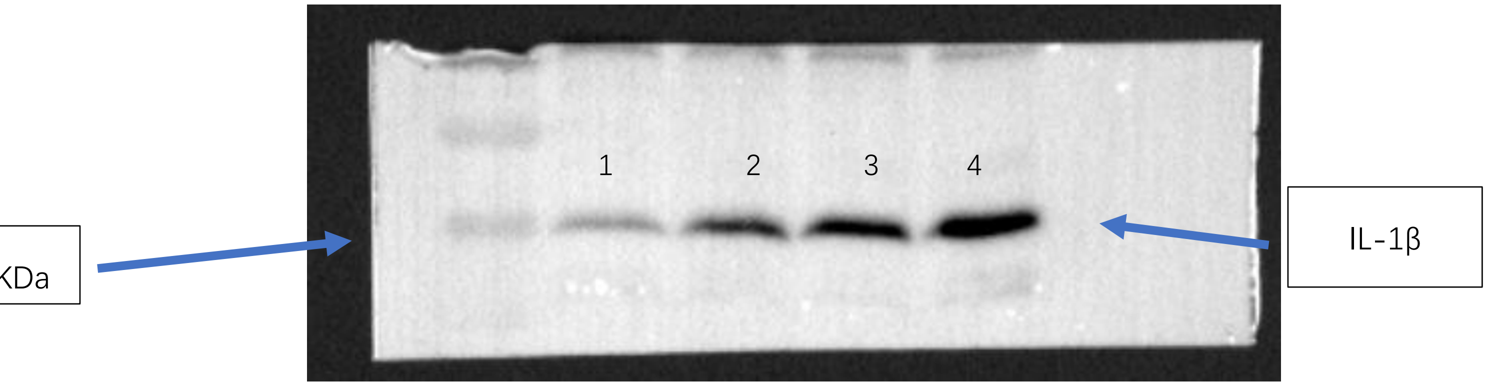

(IL-1β KDa) **Figure 6C**

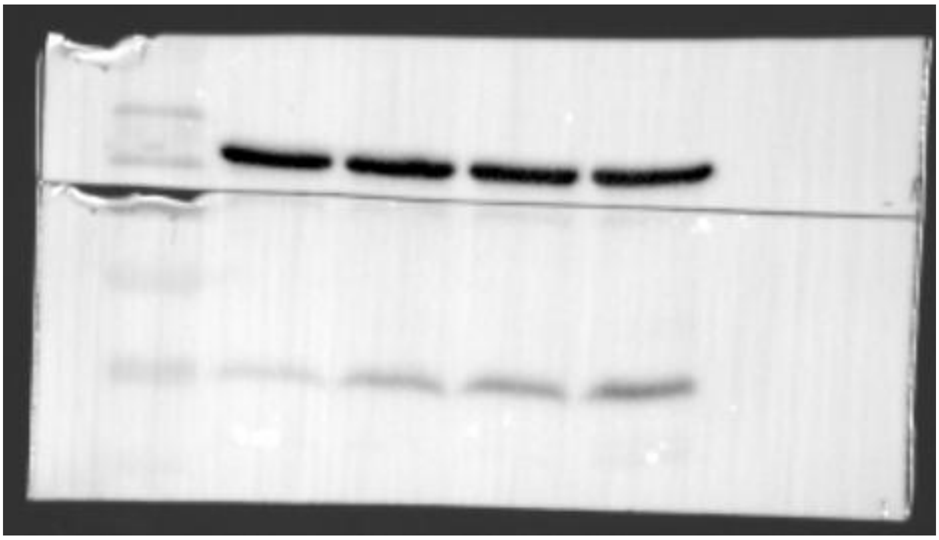

(Merged)

Group 5.

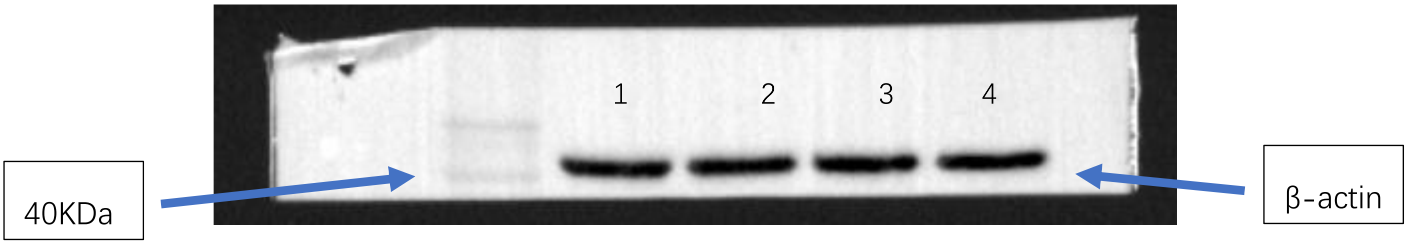

(β-actin KDa)

IL-1β

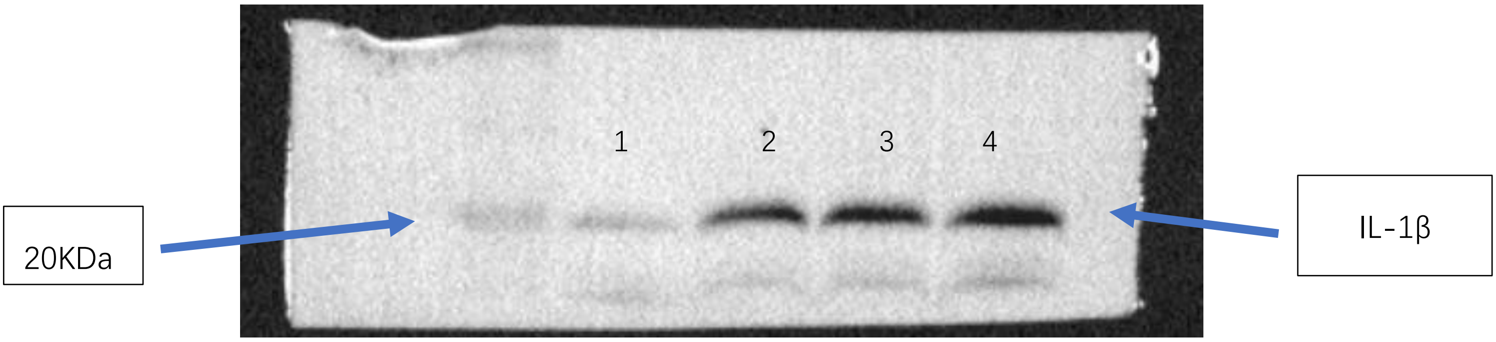

(IL-1β KDa)

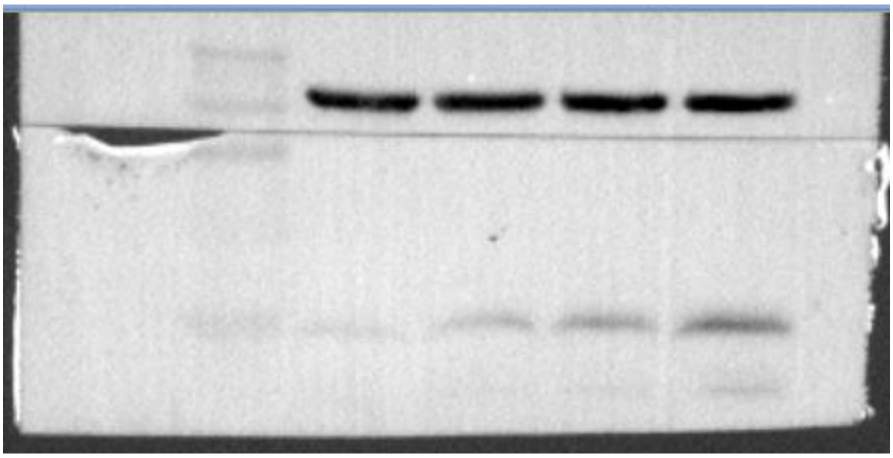

(Merged)

Western bloting-IL-6

1：Sham

2：SCI+Veh

3：SCI+AAV-NC

4：SCI+AAV-Tim-3

Group 1.

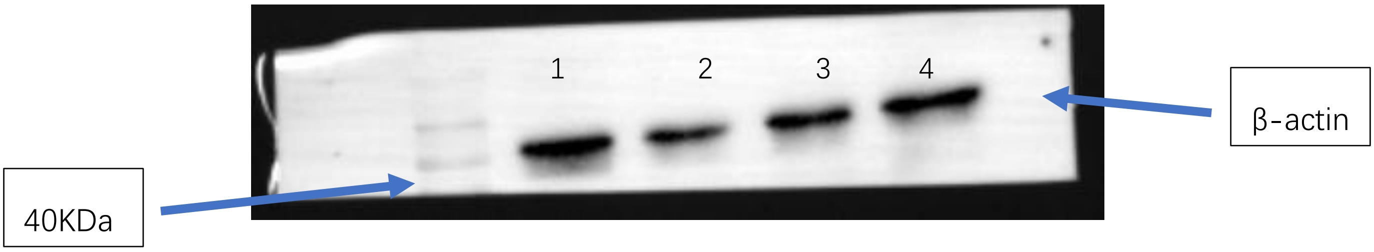

(β-actin KDa)

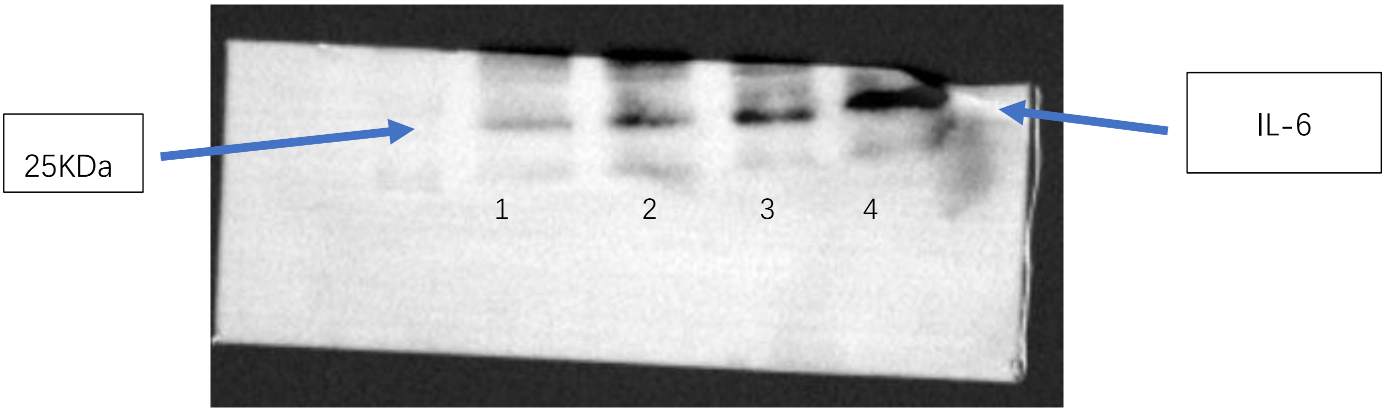

(IL-6 KDa)

IL-6

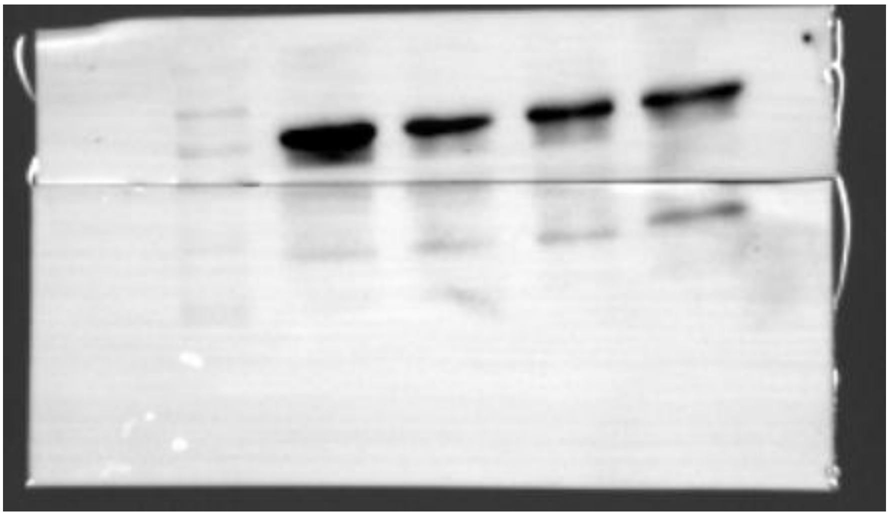

（Merged）

Group 2.

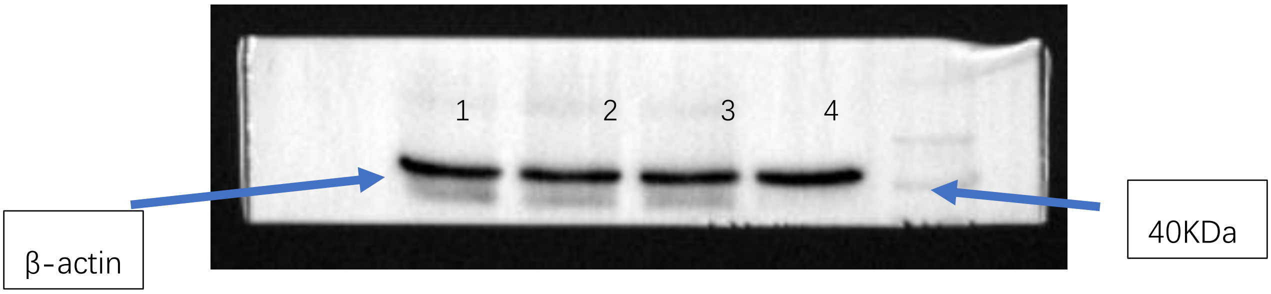

（β-actin KDa） **Figure 6B**

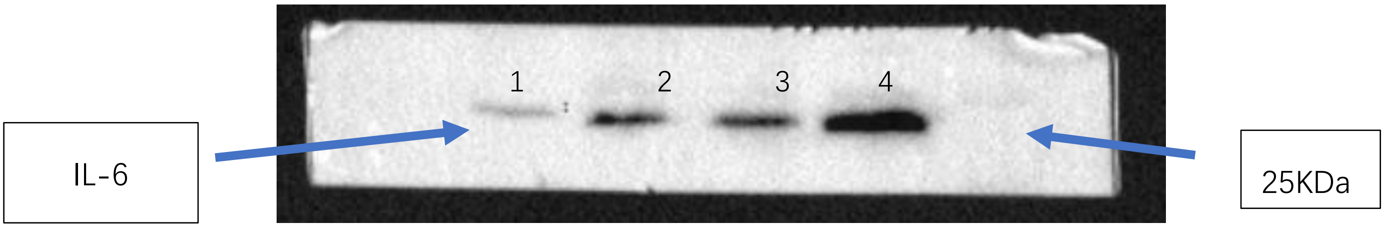

（IL-6 KDa） **Figure 6B**

IL-6

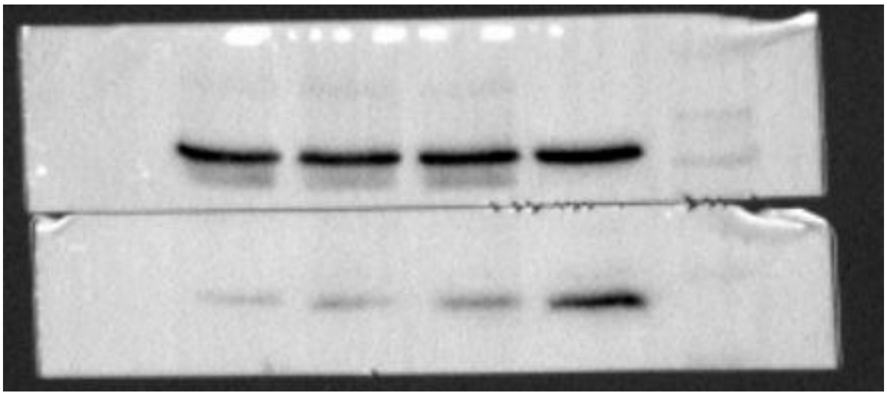

(Merged)

Group 3.

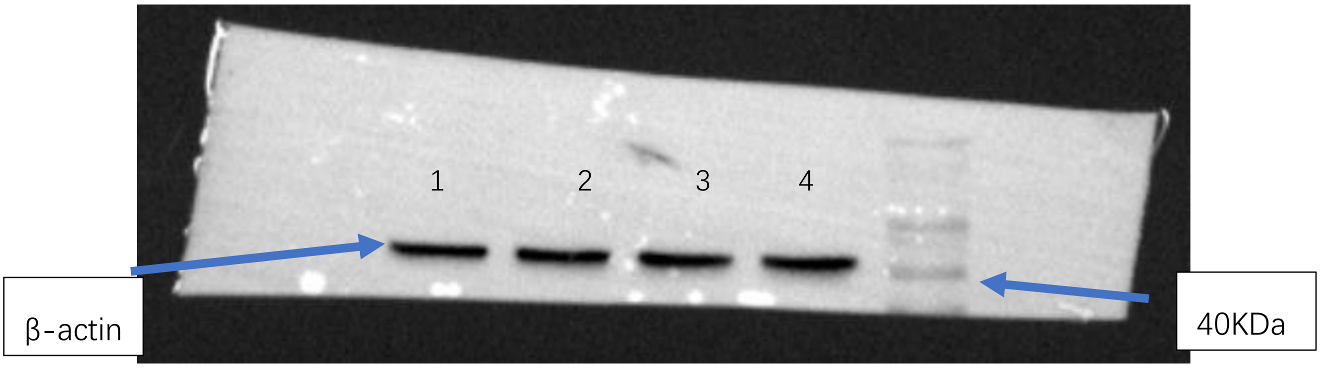

(β-actin KDa)

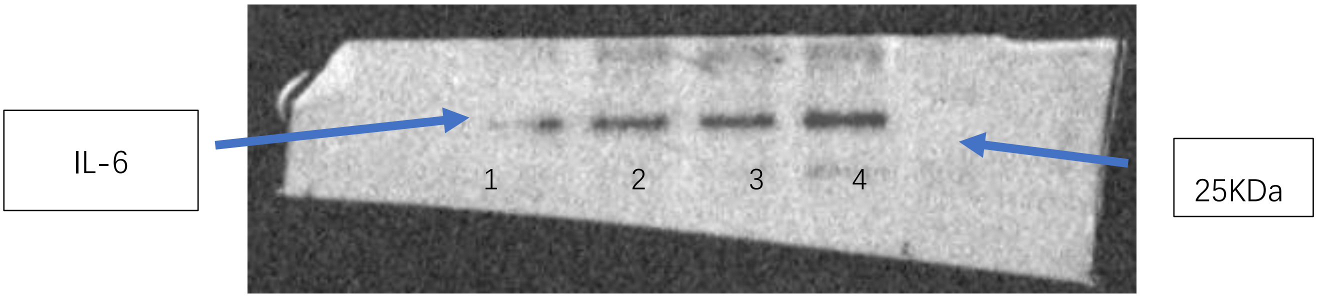

(IL-6 KDa)

IL-6

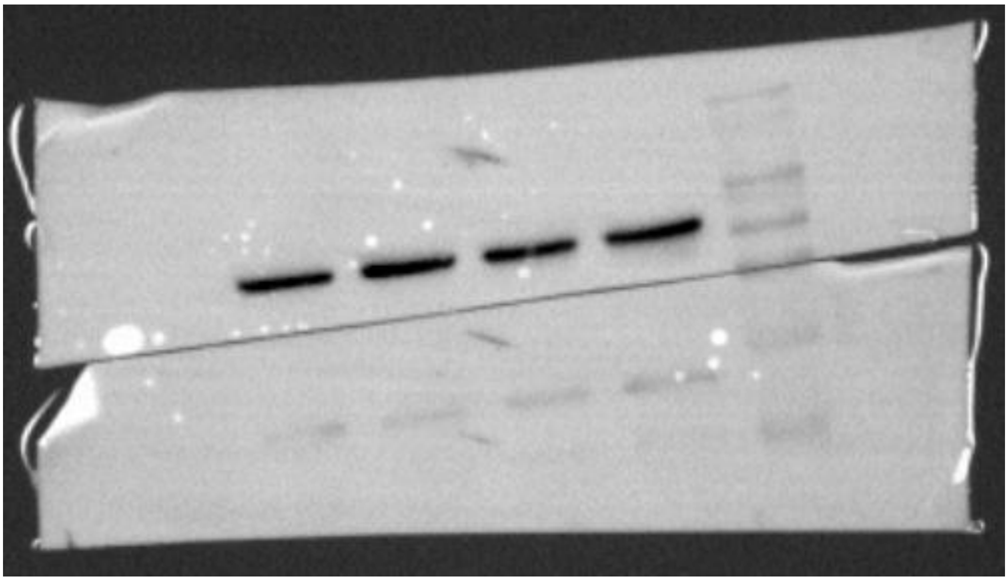

(Merged)

Group4.

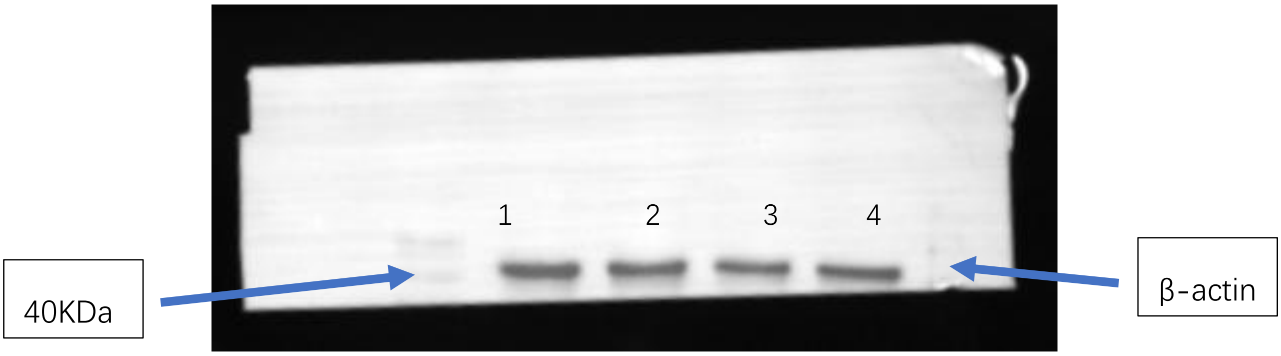

(β-actin KDa)

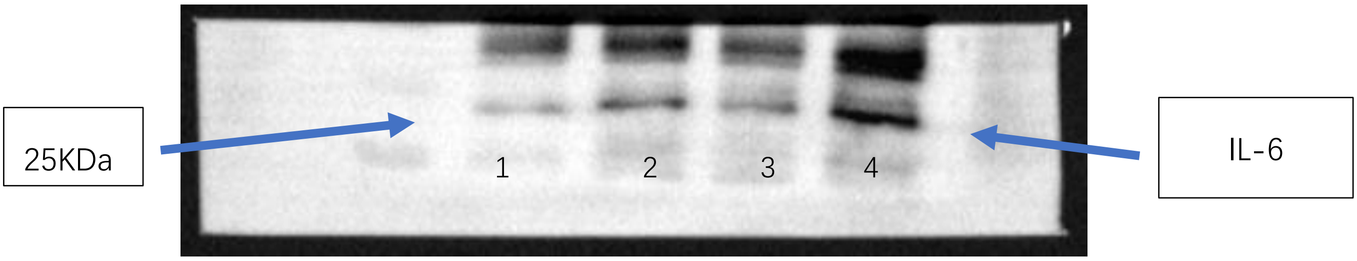

(IL-6 KDa)

IL-6

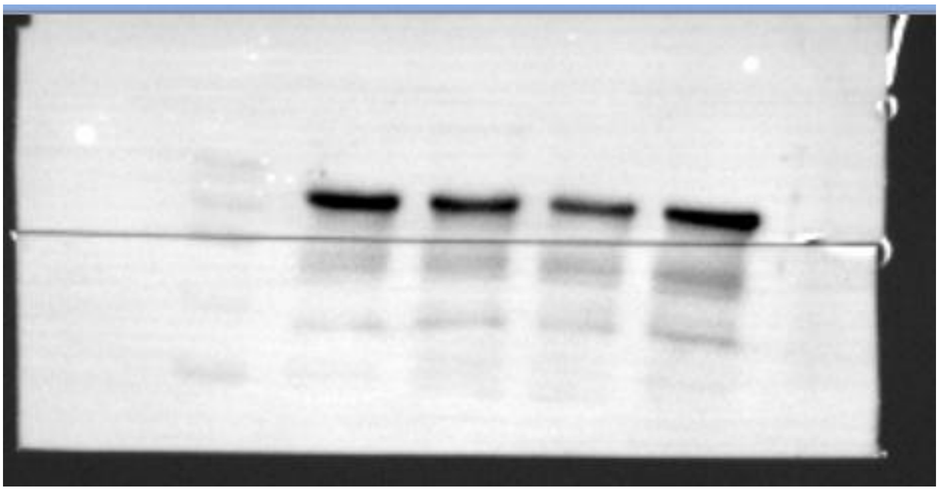

(Merged)

Group 5.

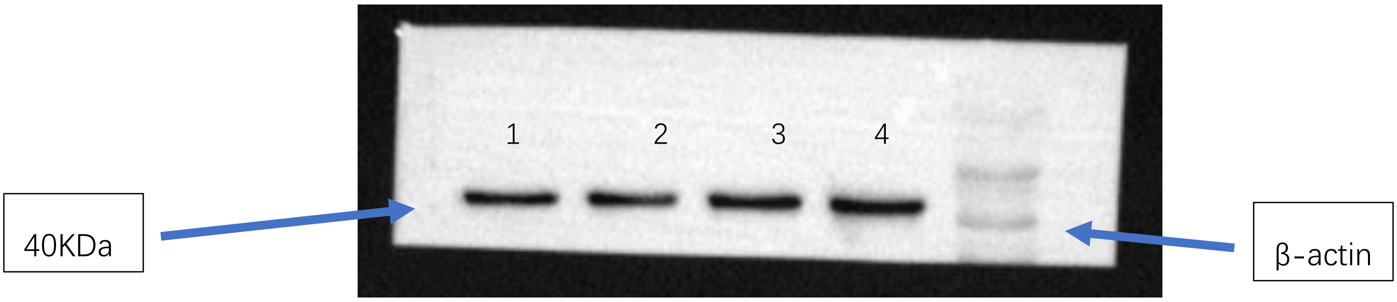

(β-actin KDa)

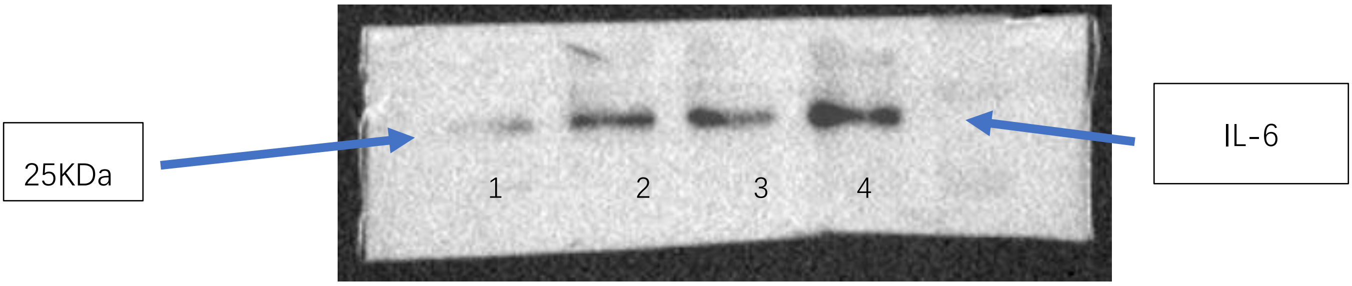

(IL-6 KDa)

IL-6

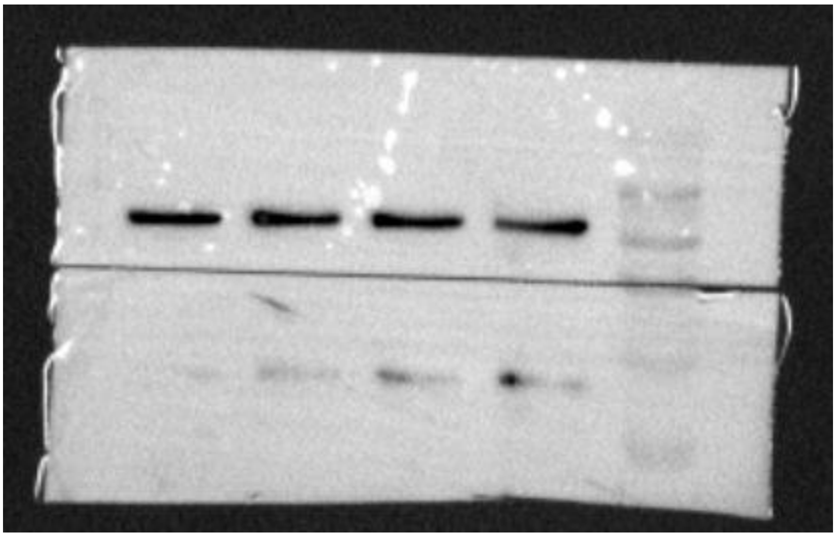

（Merged）

Western bloting- IL-17A

1： Sham

2： SCI+Veh

3： SCI+AAV-NC

4： SCI+AAV-Tim-3

Group 1.

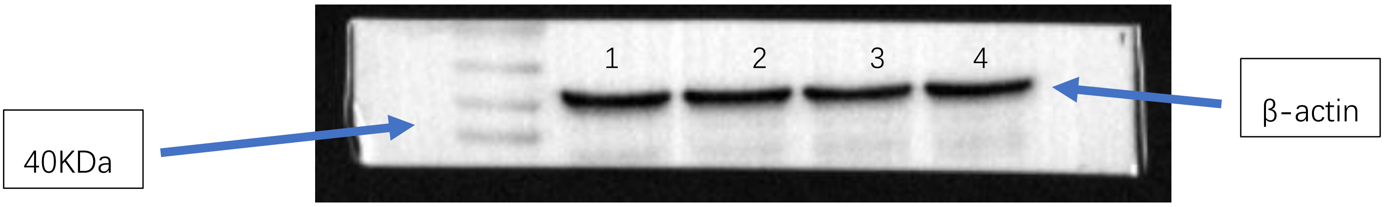

(β-actin KDa)

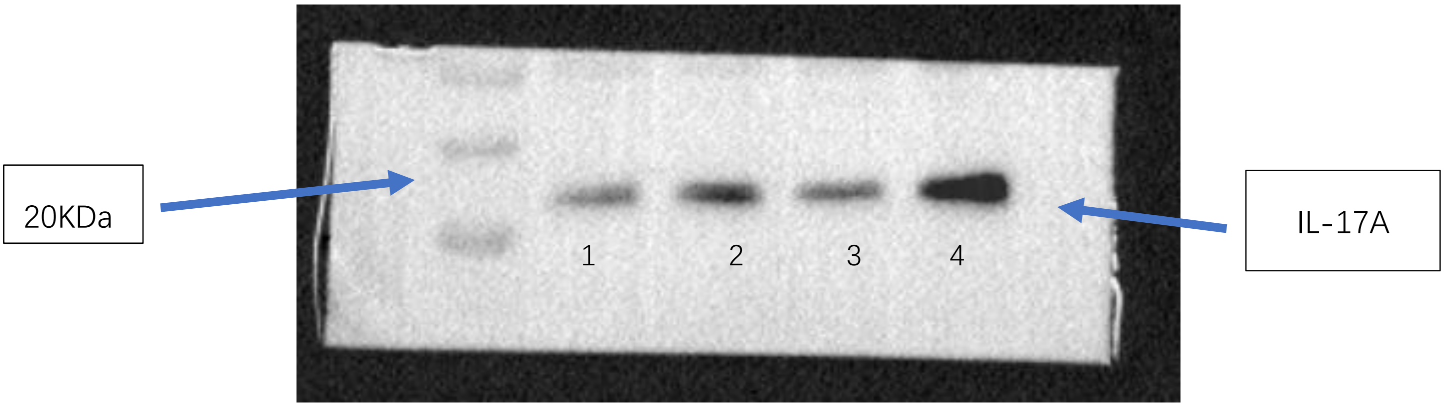

(IL-17A KDa)

IL-17A

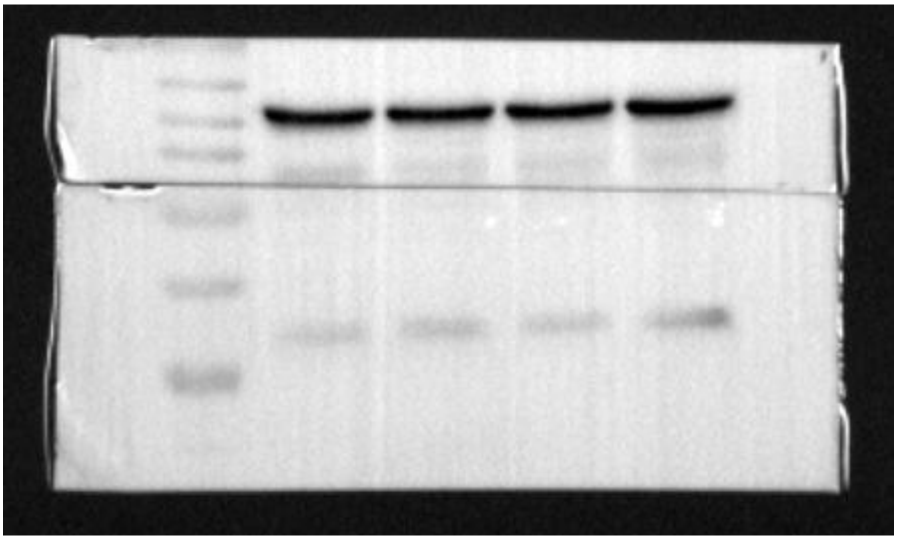

(Merged)

Group 2.

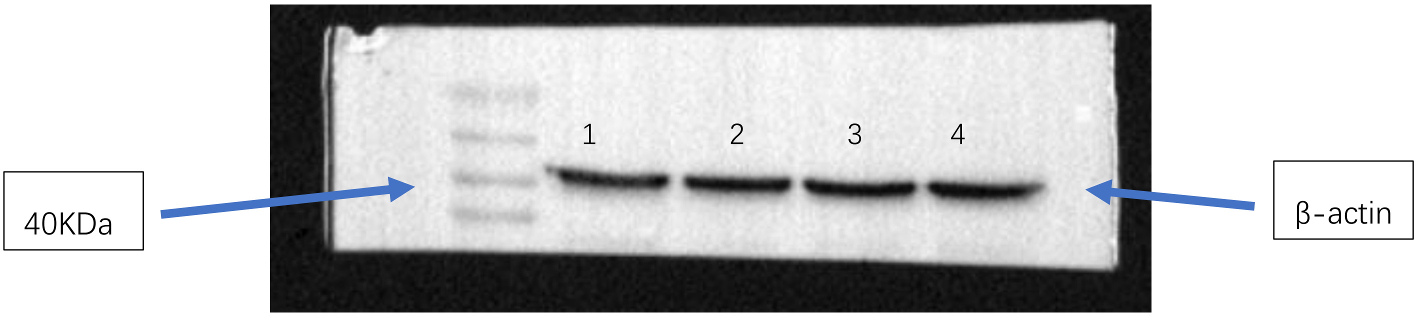

(β-actin KDa) **Figure 6B**

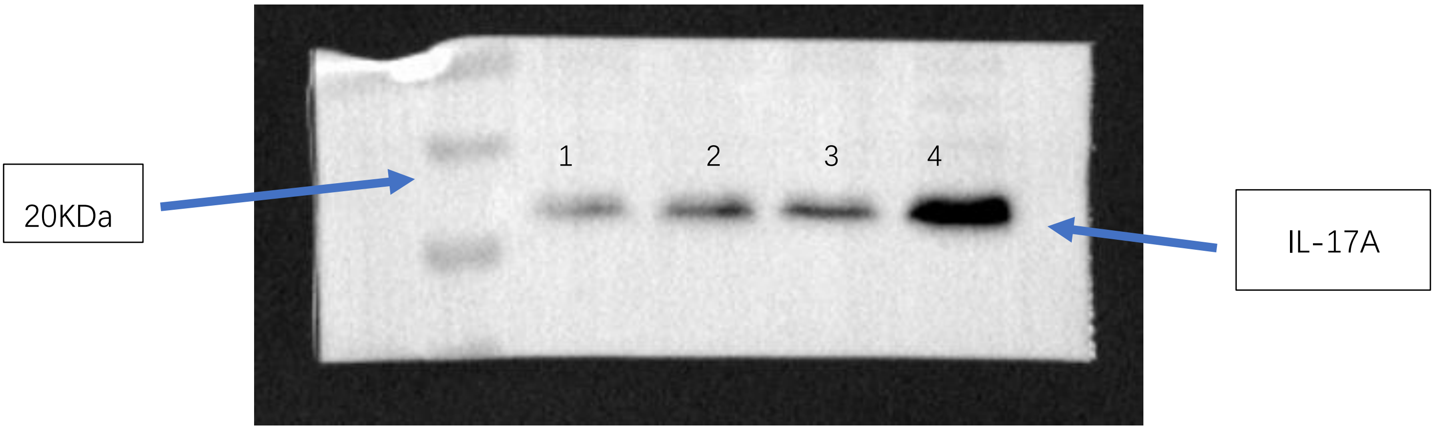

(IL-17A KDa) **Figure 6B**

IL-17A

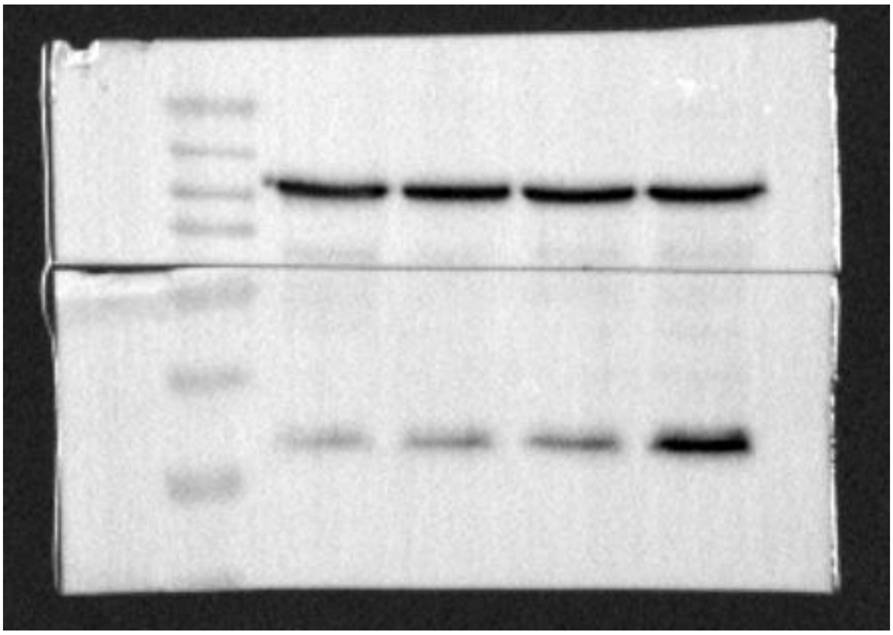

(Merged)

Group 3.

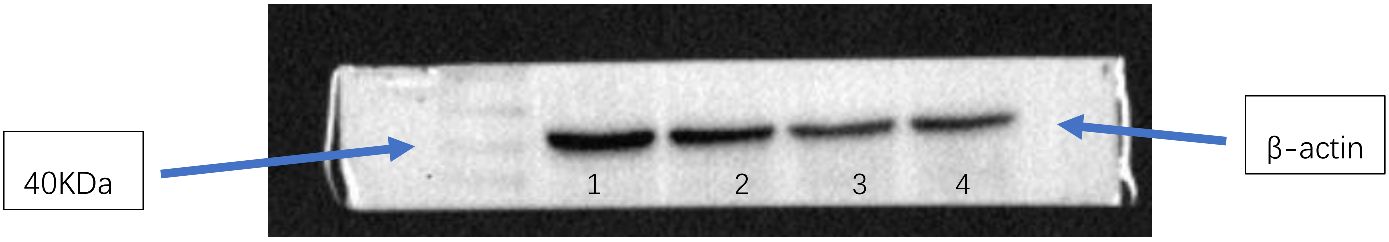

(β-actin KDa)

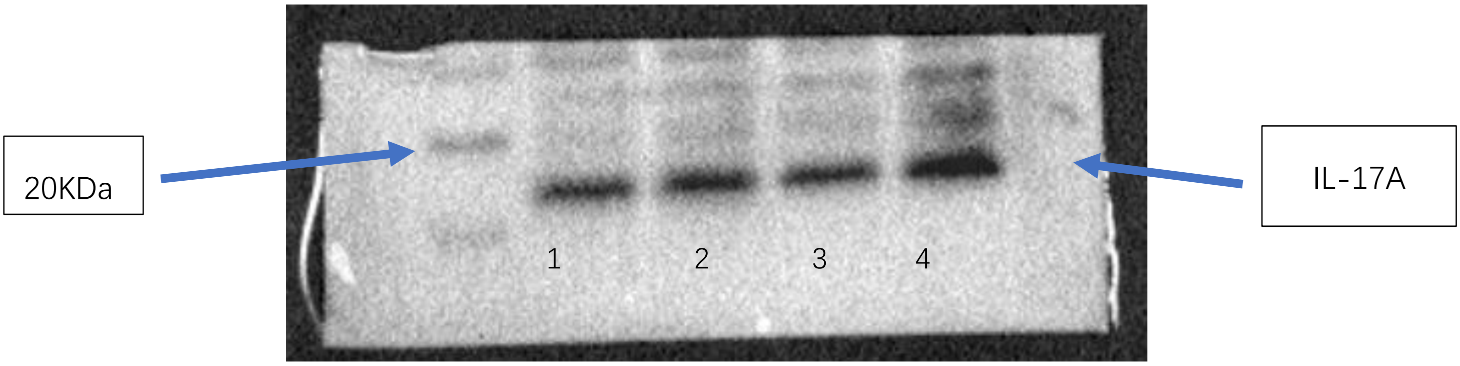

(IL-17A KDa)

IL-17A

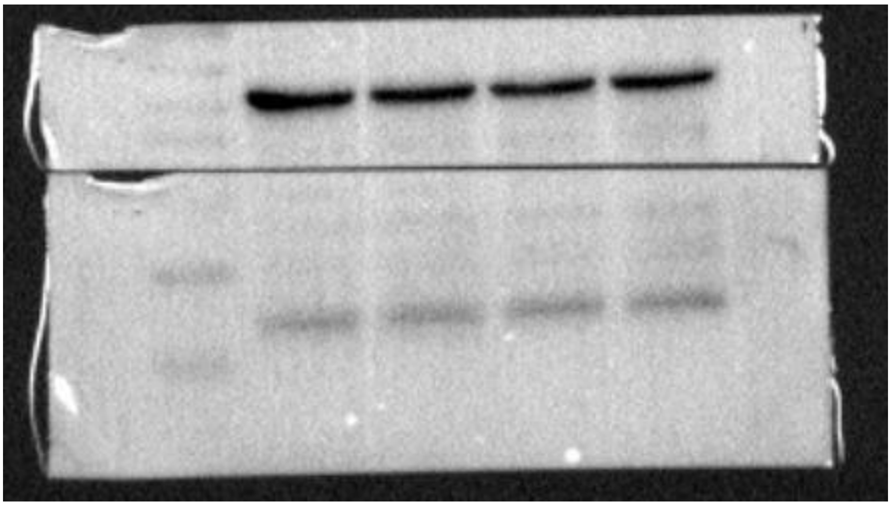

(Merged)

Group4.

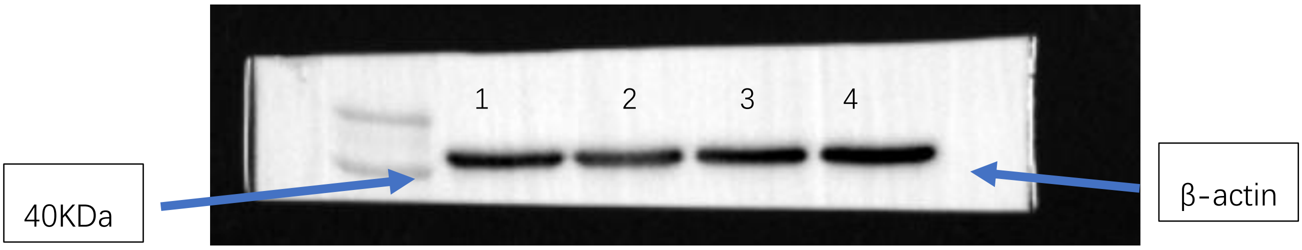

(β-actin KDa)

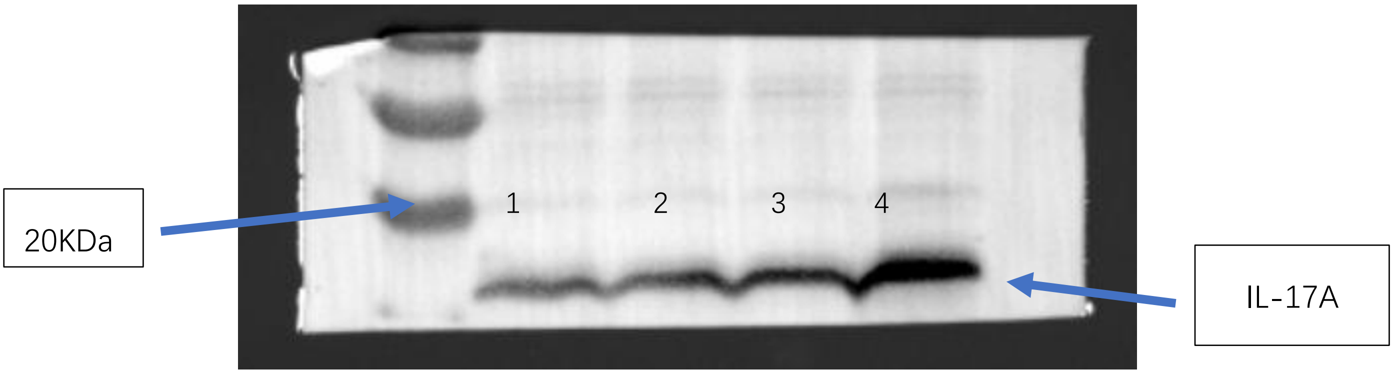

(IL-17A KDa)

IL-17A

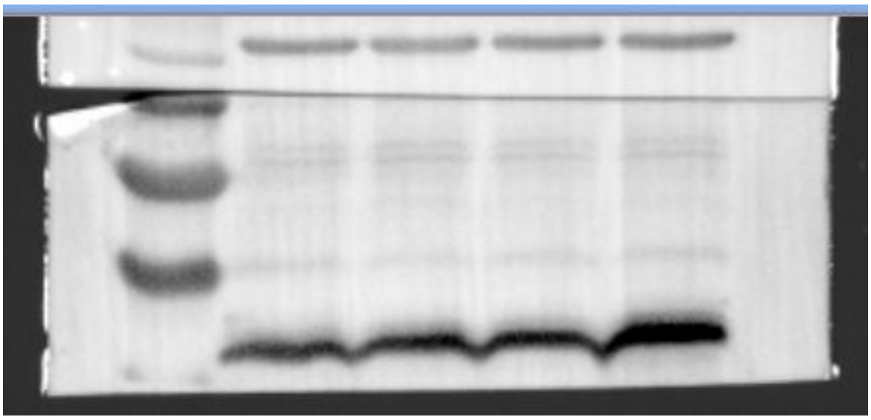

(Merged)

Group 5.

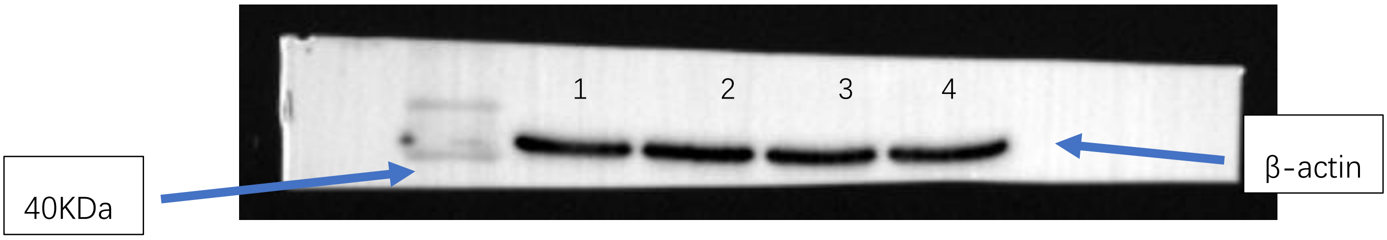

(β-actin KDa)

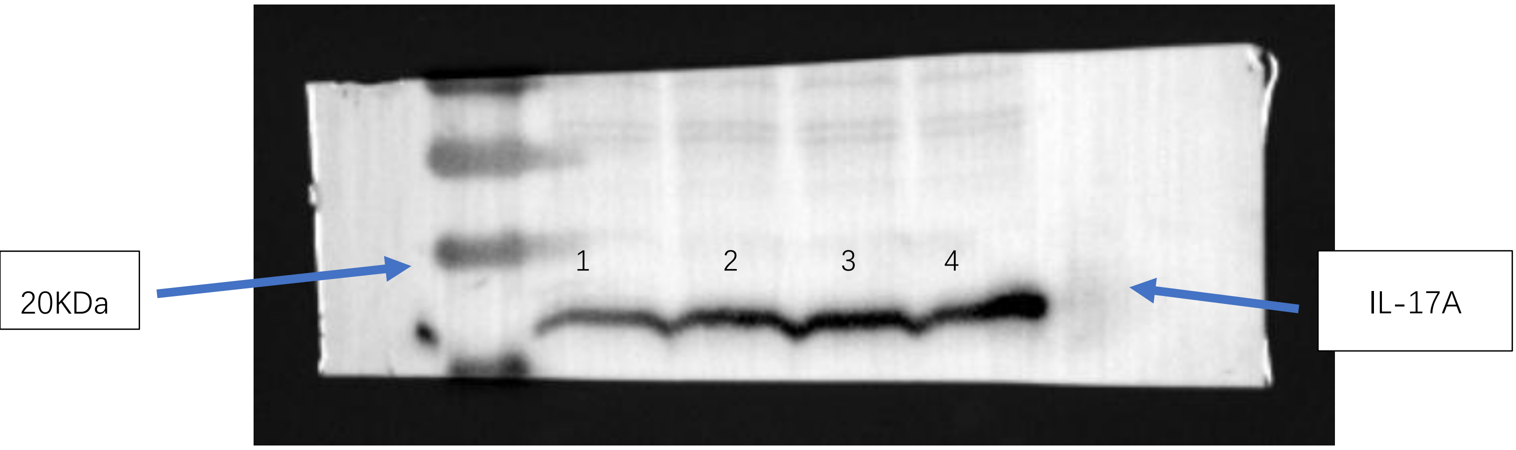

(IL-17A KDa)

IL-17A

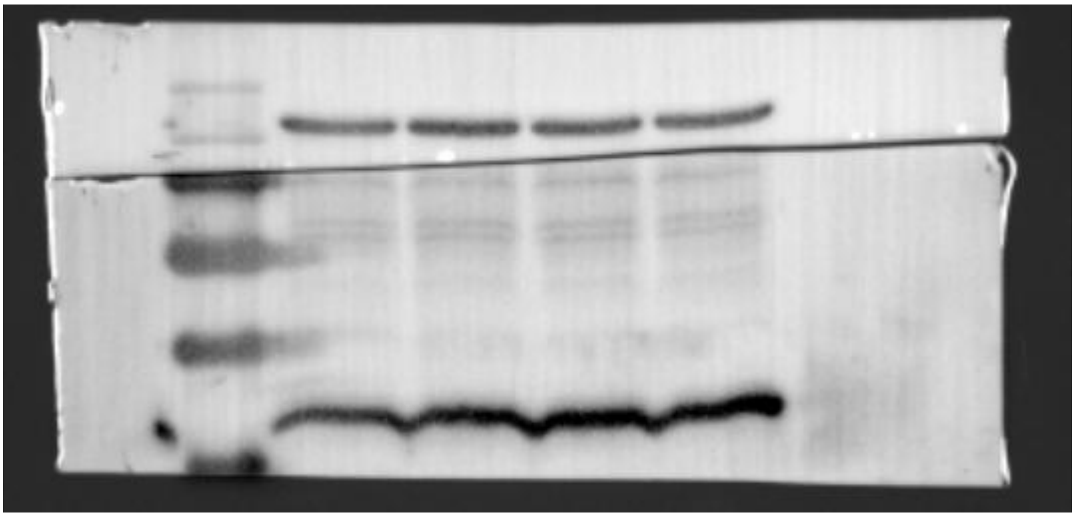

(Merged)

Western bloting-iNOS

1：SCI+Veh

2：SCI+AAV-NC

3：SCI+AAV-Tim-3

Group 1.

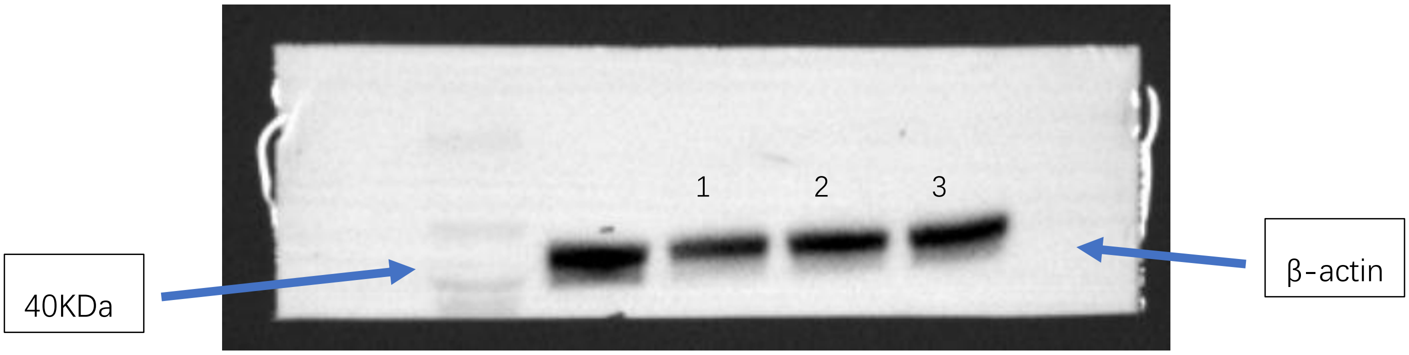

(β-actin KDa)

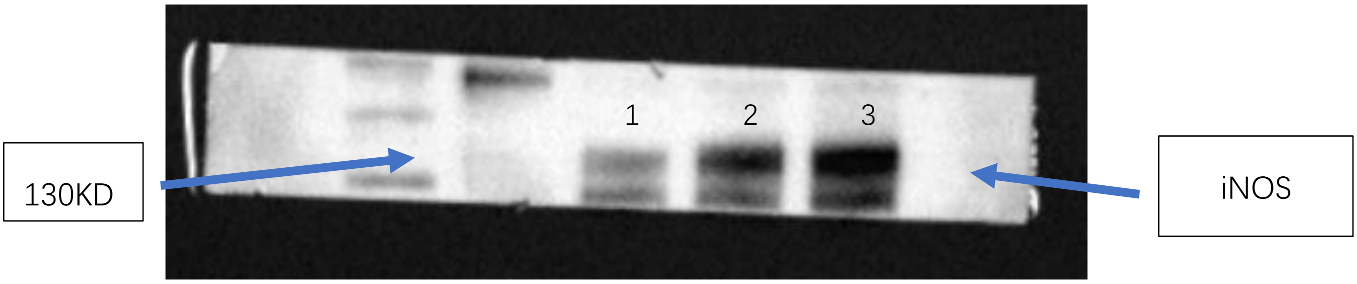

(iNOS KDa)

iNOS

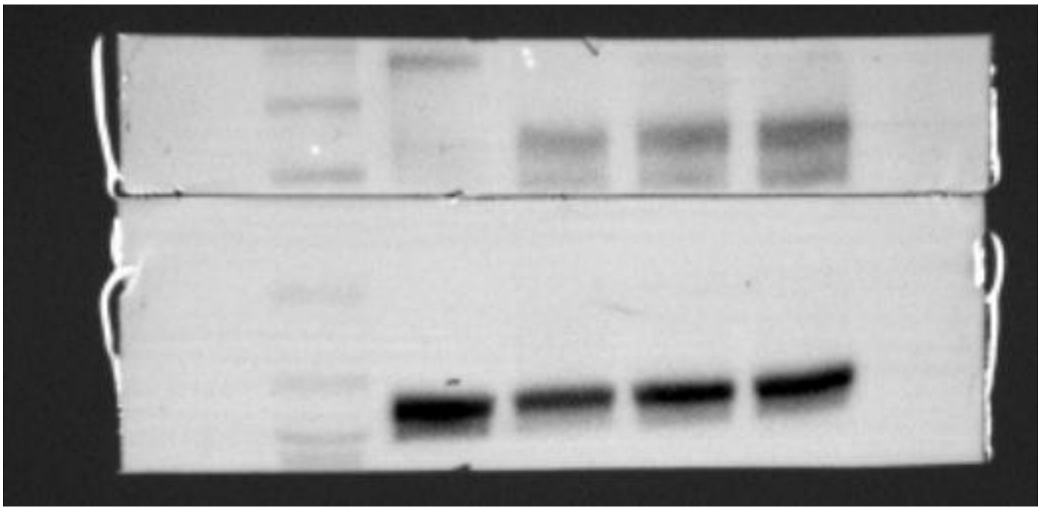

(Merged)

Group 2.

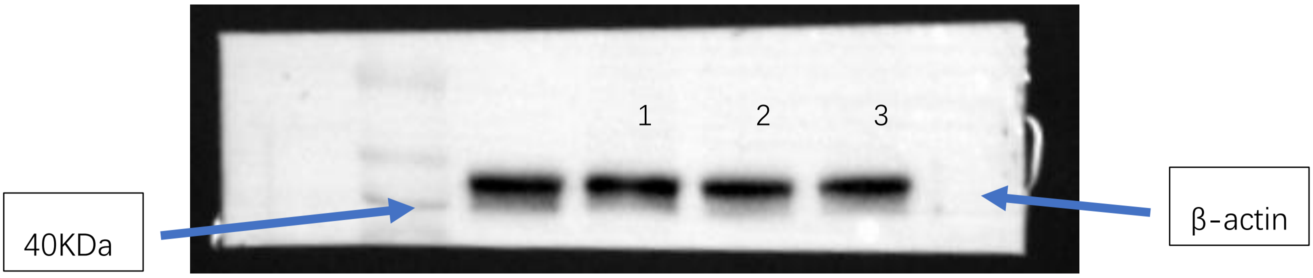

(β-actin KDa) **Figure 7E**

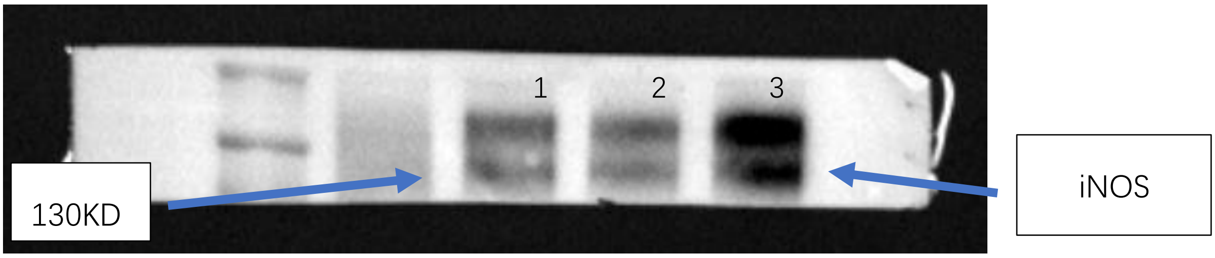

(iNOS KDa) **Figure 7E**

iNOS

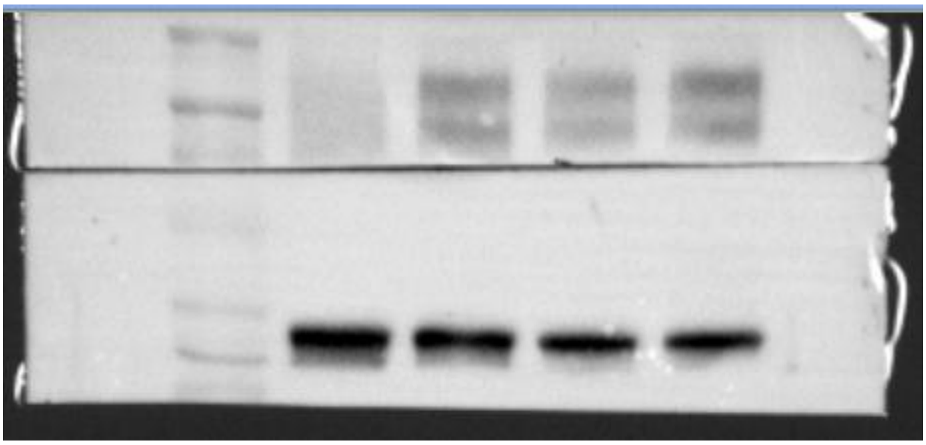

(Merged)

Group 3.

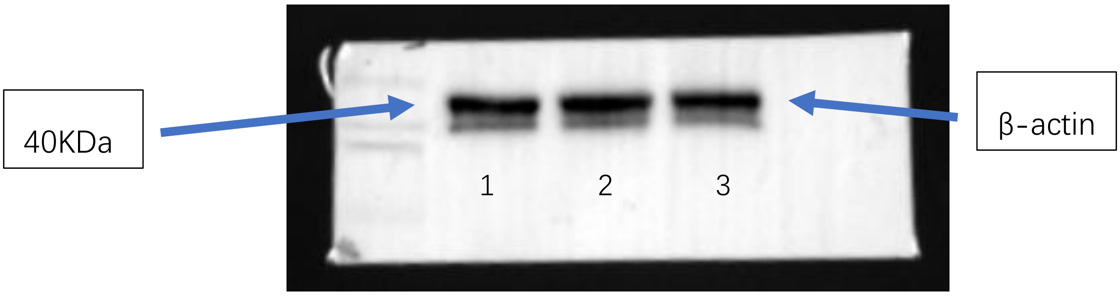

(β-actin KDa)

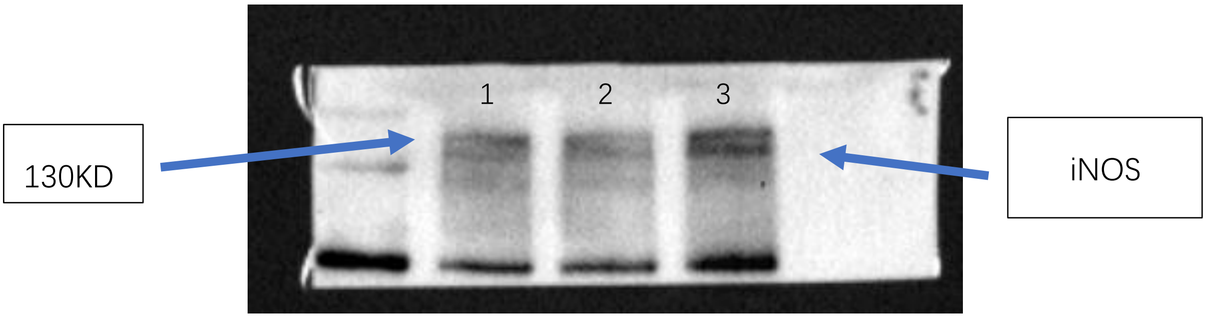

(iNOS KDa)

iNOS

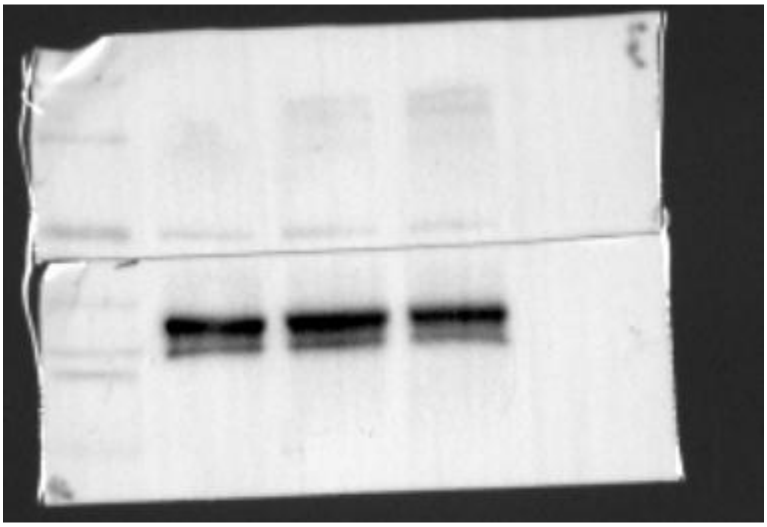

(Merged)

Group4.

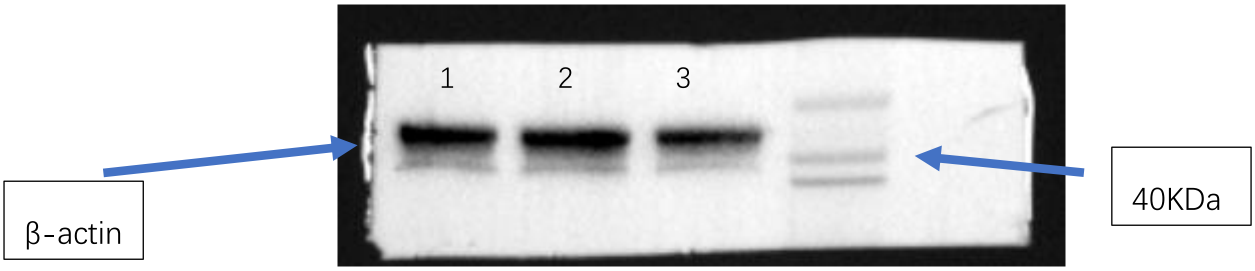

(β-actin KDa)

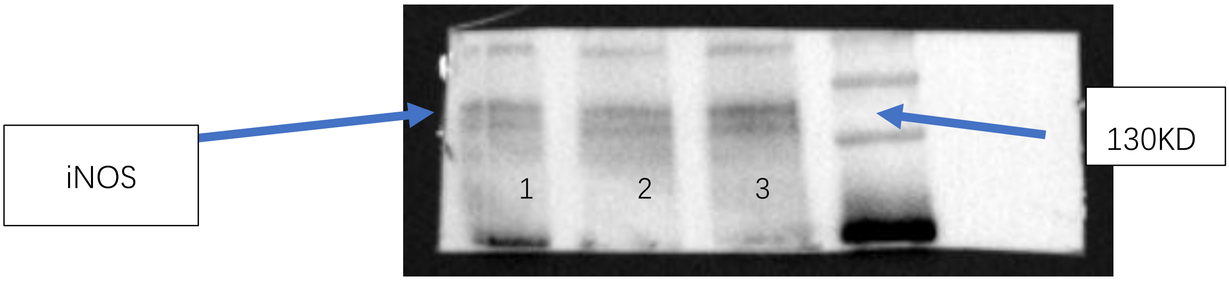

(iNOS KDa)

iNOS

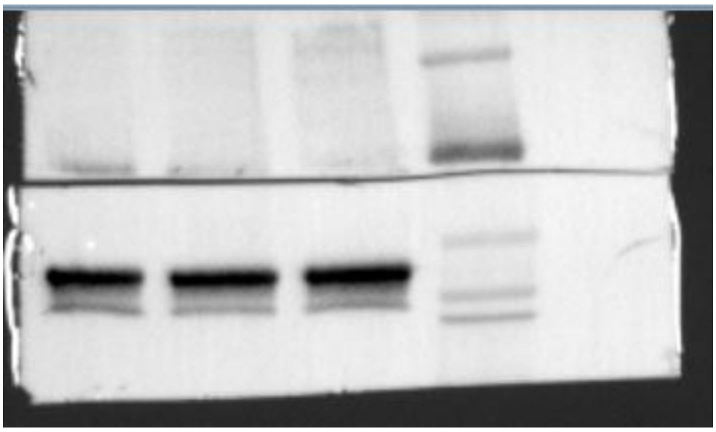

(Merged)

Group 5.

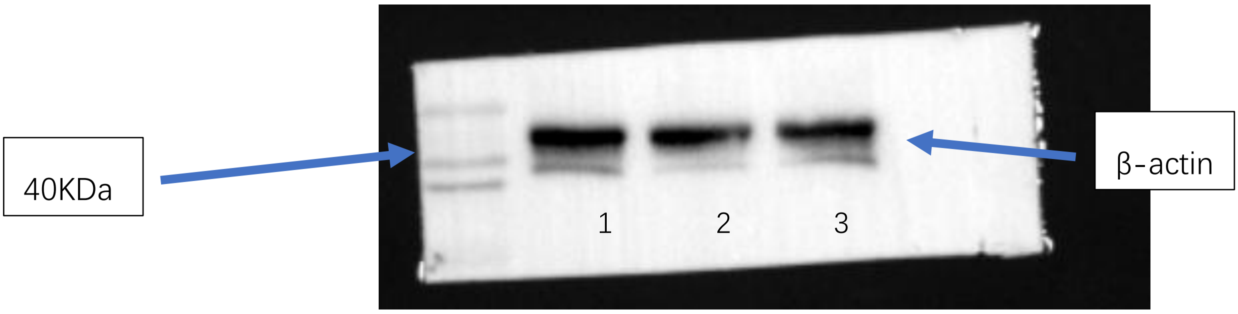

(β-actin KDa)

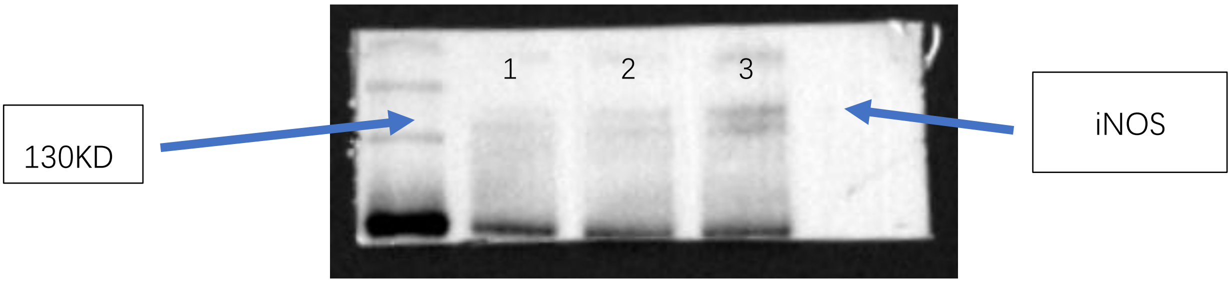

(iNOS KDa)

iNOS

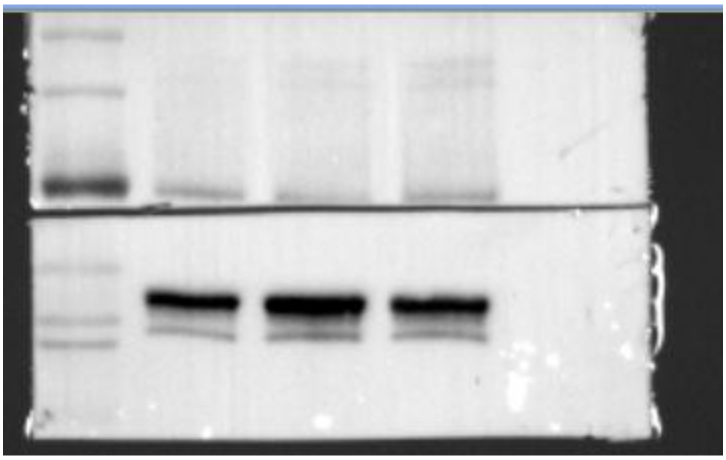

（Merged）

Western bloting-Tim-3

1：Sham

2：1d

3：3d

4：7d

Group 1.

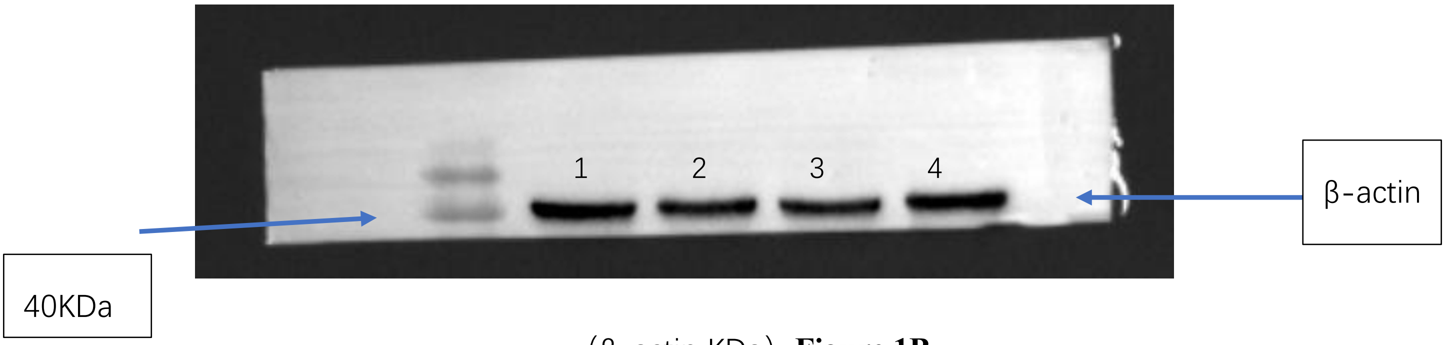

(β-actin KDa) **Figure 1B**

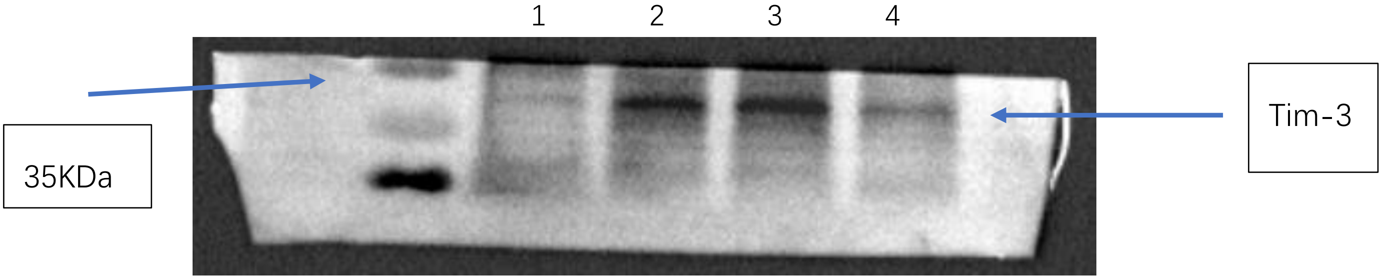

(Tim-3 KDa) **Figure 1B**

Tim-3 Sham、1d、3d、7d

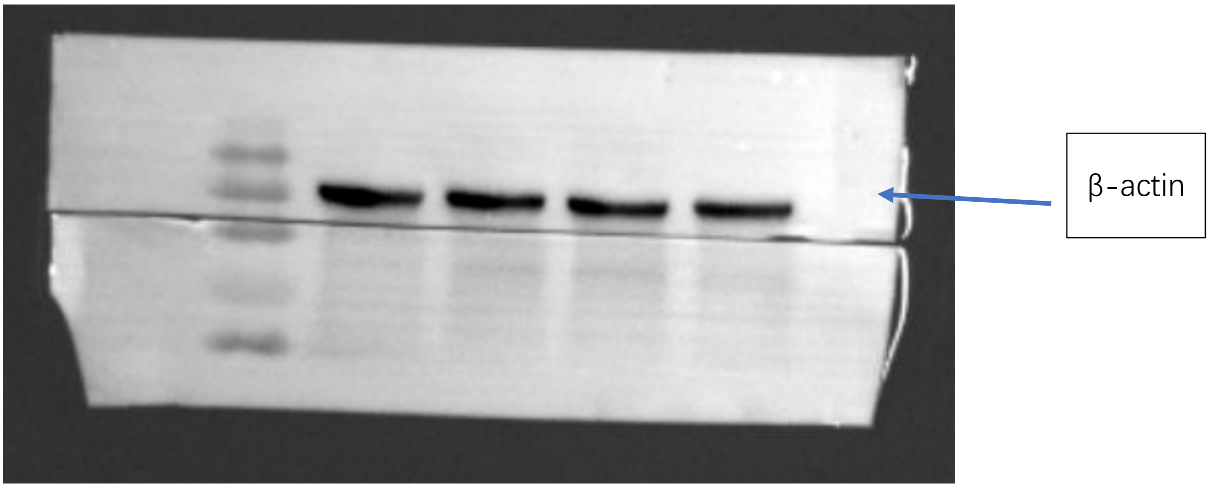

(Merged)

Group 2.

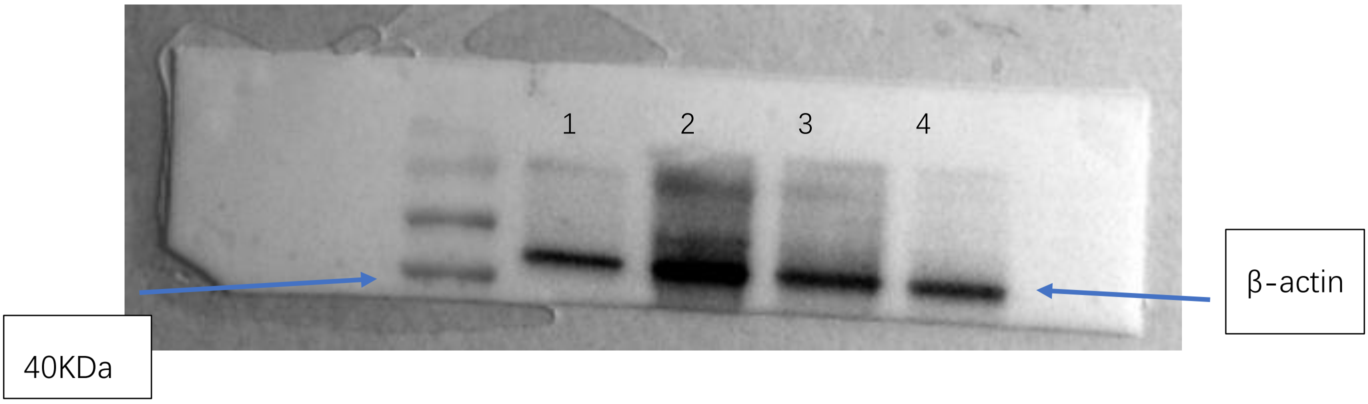

(β-actin KDa)

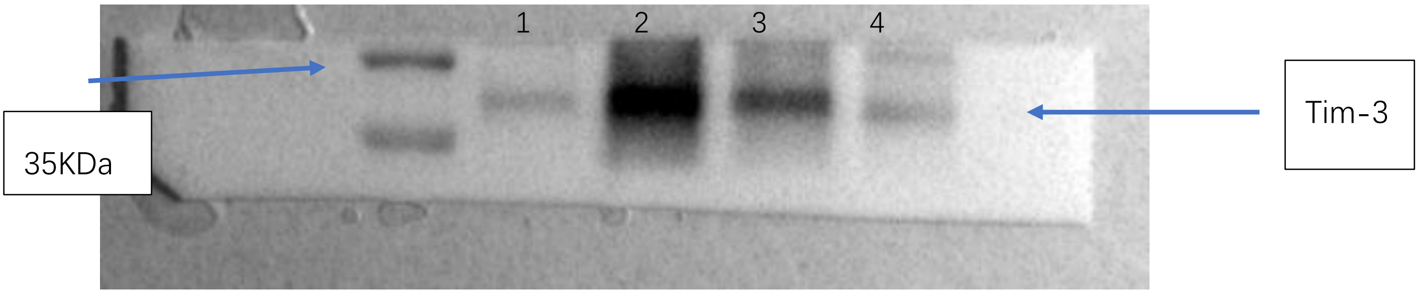

(Tim-3 KDa)

Tim-3 Sham、1d、3d、7d

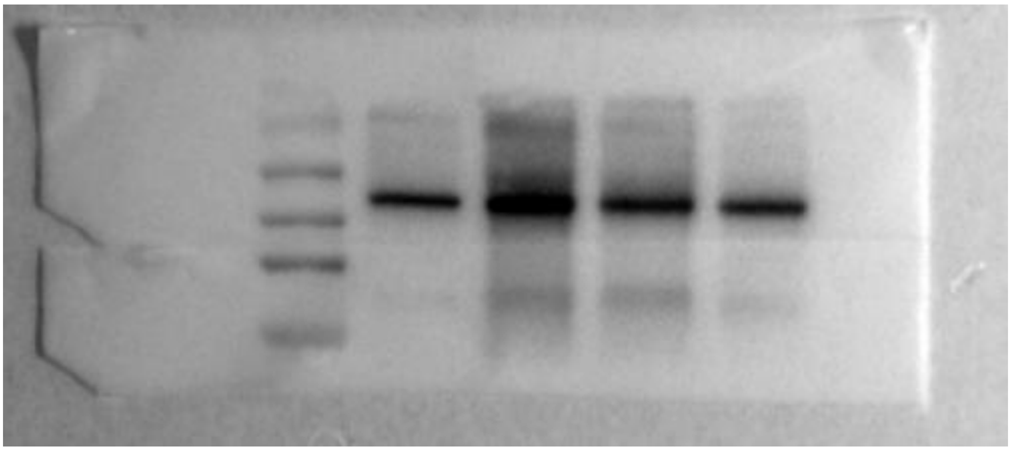

（Merged）

Group 3.

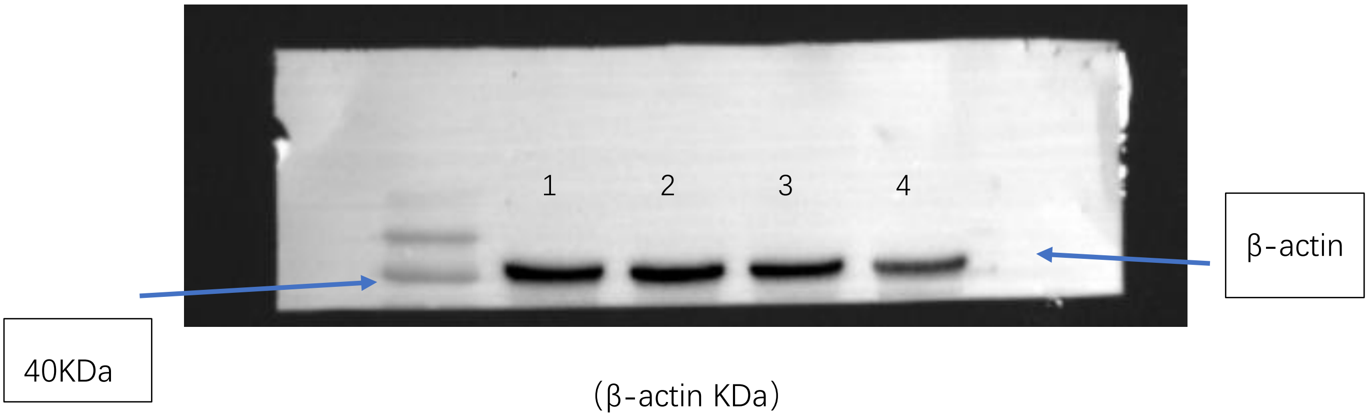

（β-actin KDa）

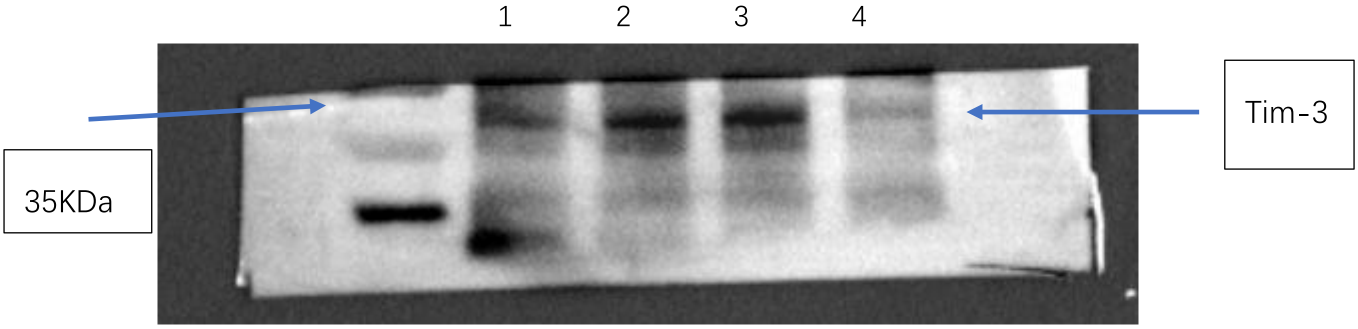

（Tim-3 KDa）

Tim-3 Sham、1d、3d、7d

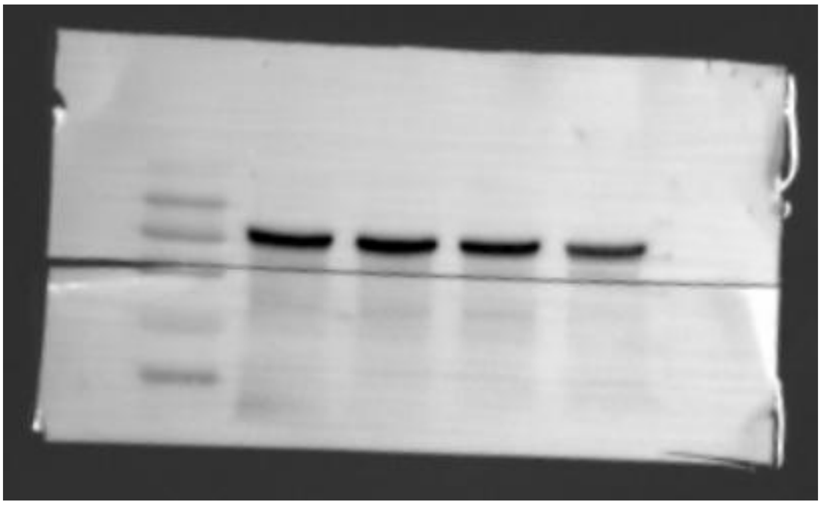

（Merged）

Western bloting-HMGB1

1：Sham

2：SCI+Veh

3：SCI+AAV-Tim-3

4：SCI+AAV-Tim-3+ Oltipraz

Group 1.

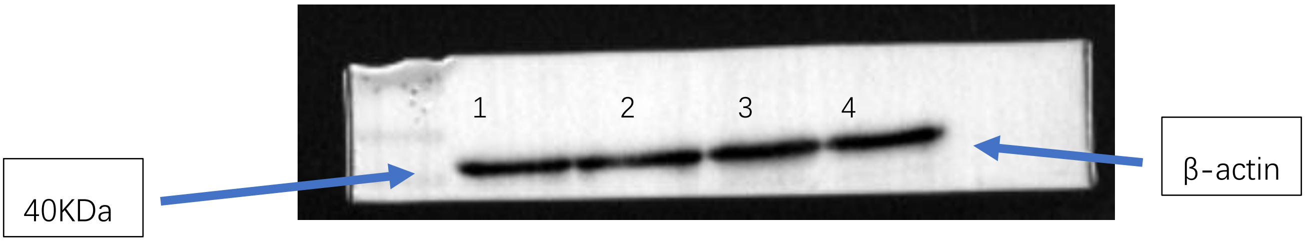

（β-actin KDa）

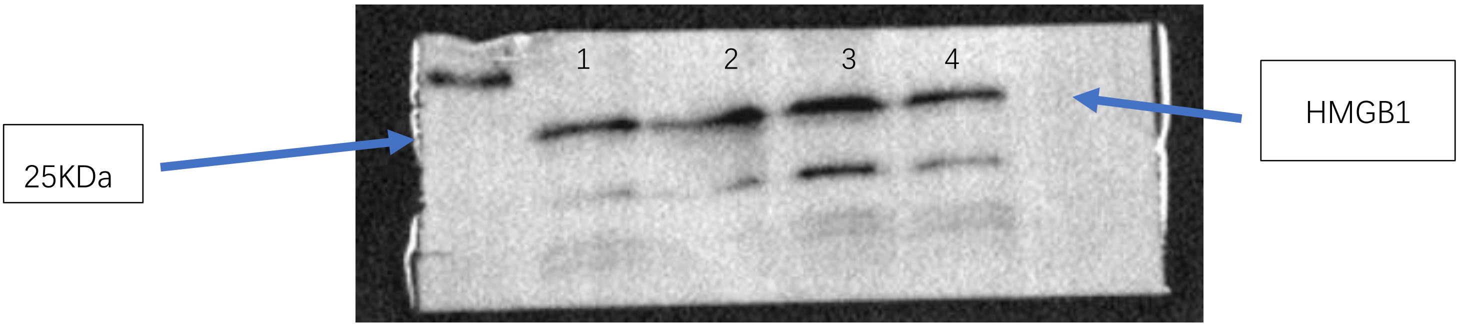

（HMGB1 KDa）

HMGB1 （Oltipraz）

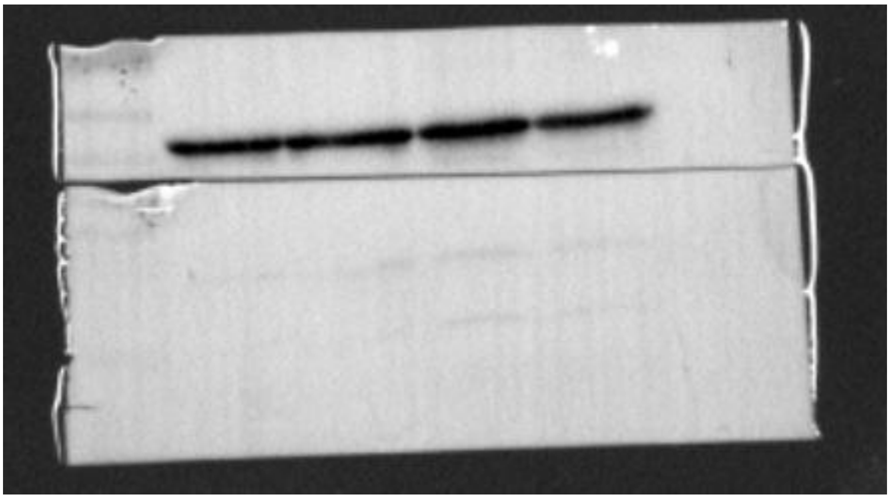

（Merged）

Group 2.

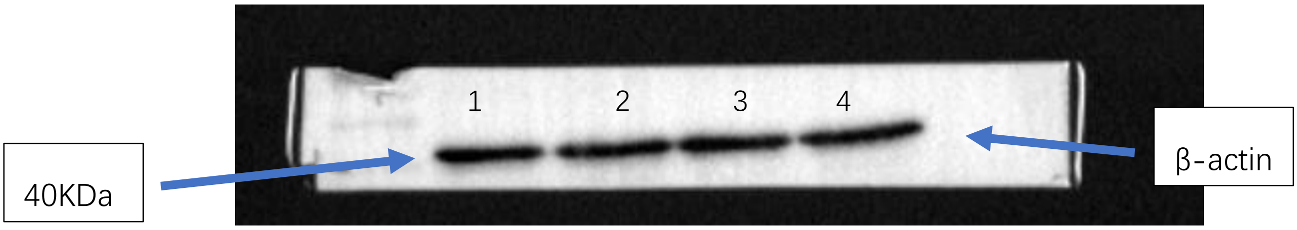

（β-actin KDa）

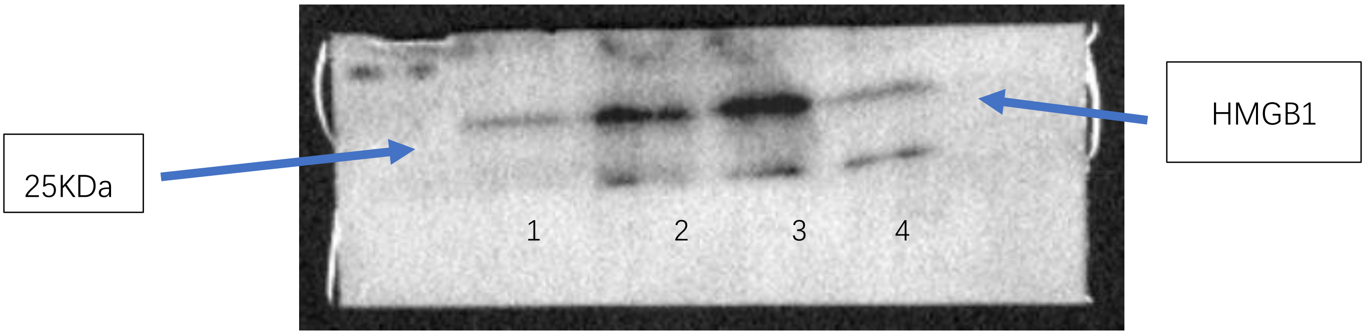

（HMGB1KDa）

HMGB1 （Oltipraz）

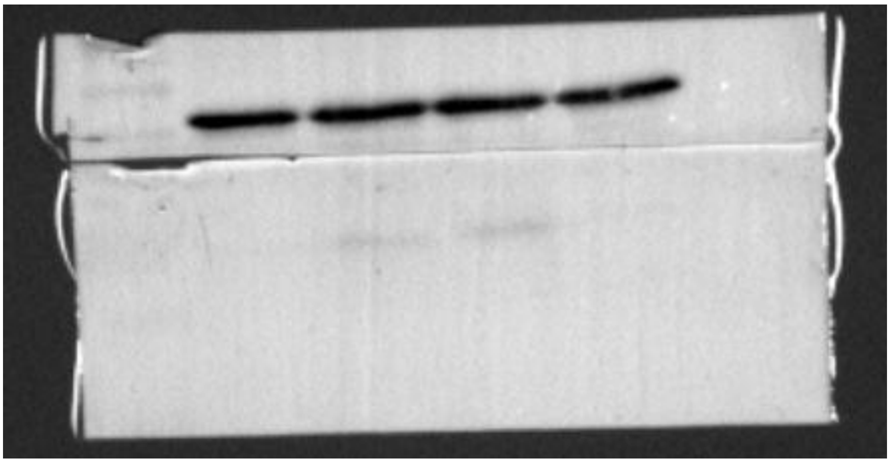

（Merged）

Group 3.

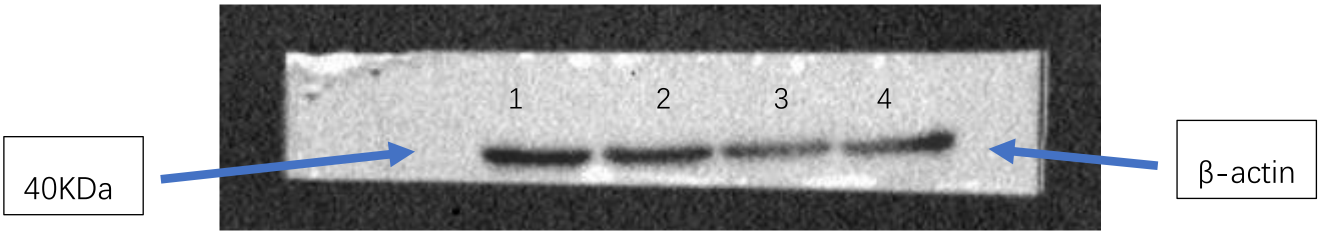

（β-actin KDa） **Figure 8C**

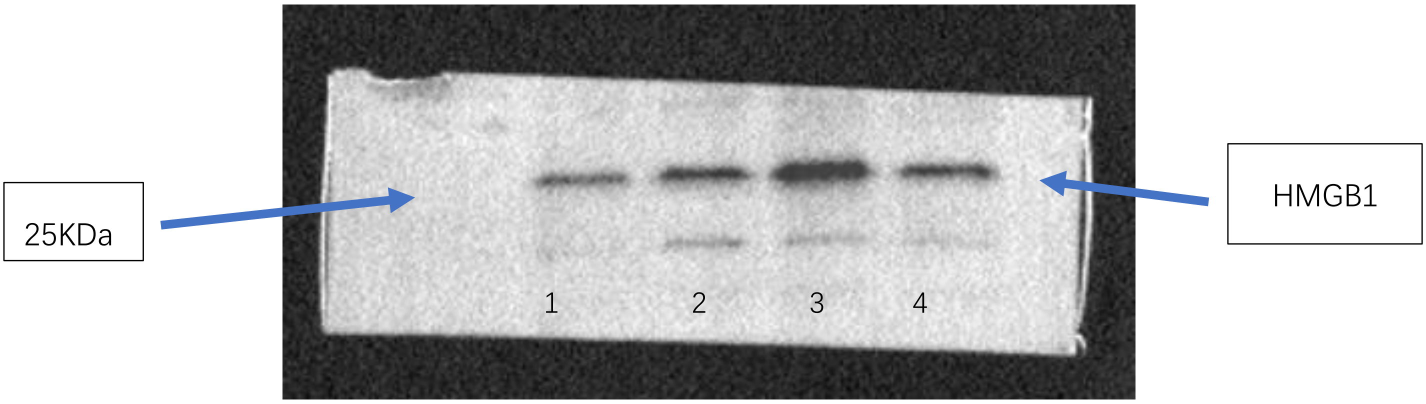

（HMGB1 KDa） **Figure 8C**

HMGB1 （Oltipraz）

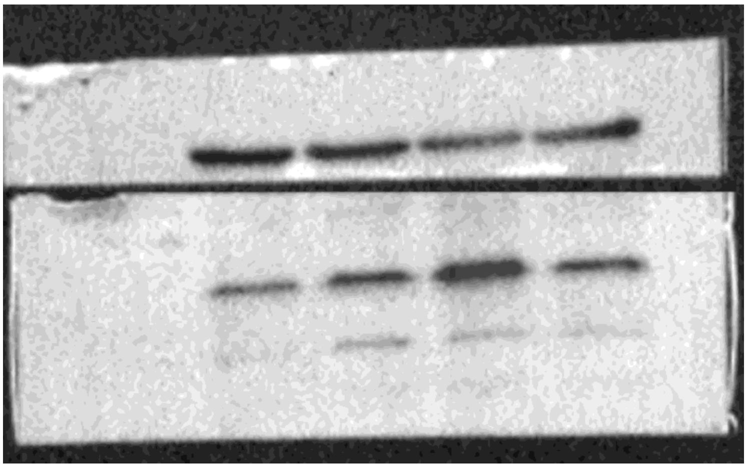

（Merged）

Group4.

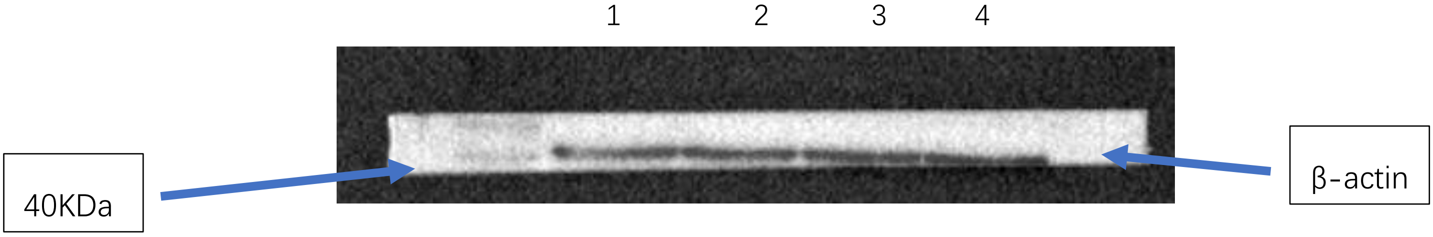

（β-actin KDa）

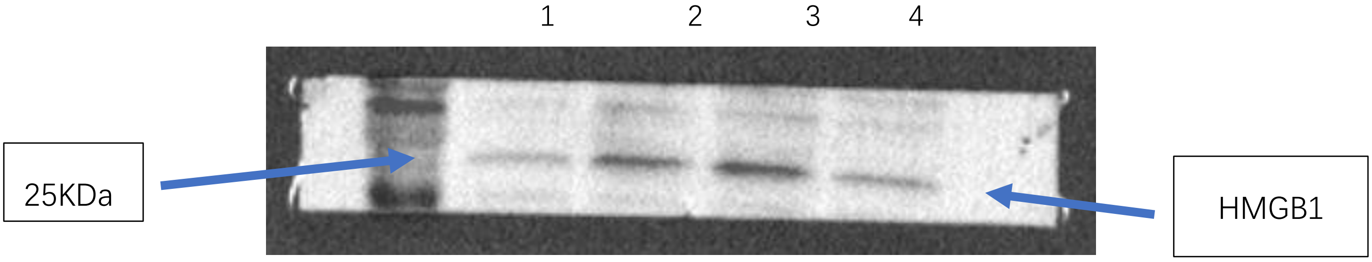

（HMGB1 KDa）

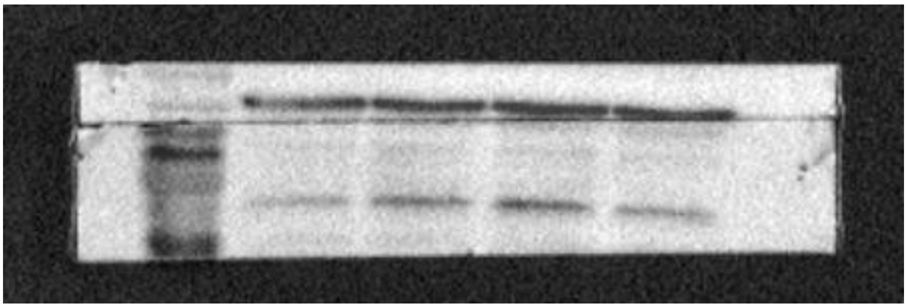

（Merged）

HMGB1 （Oltipraz）

Group 5.

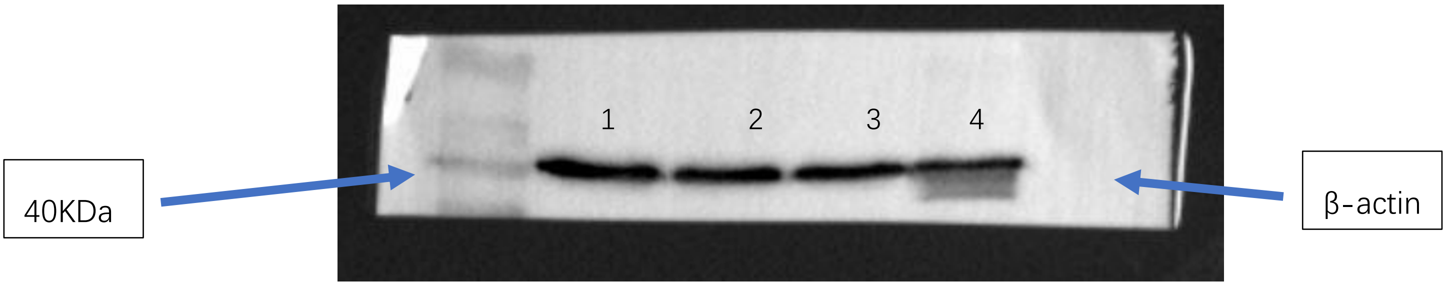

（β-actin KDa）

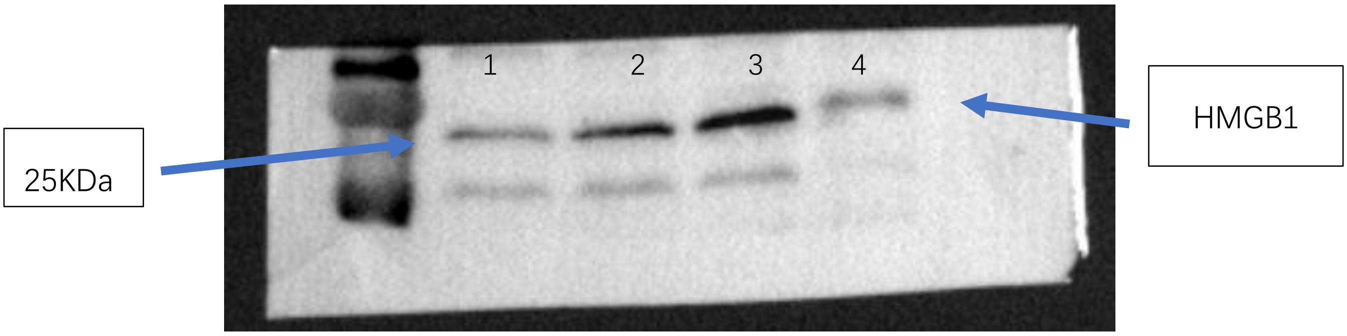

（HMGB1 KDa）

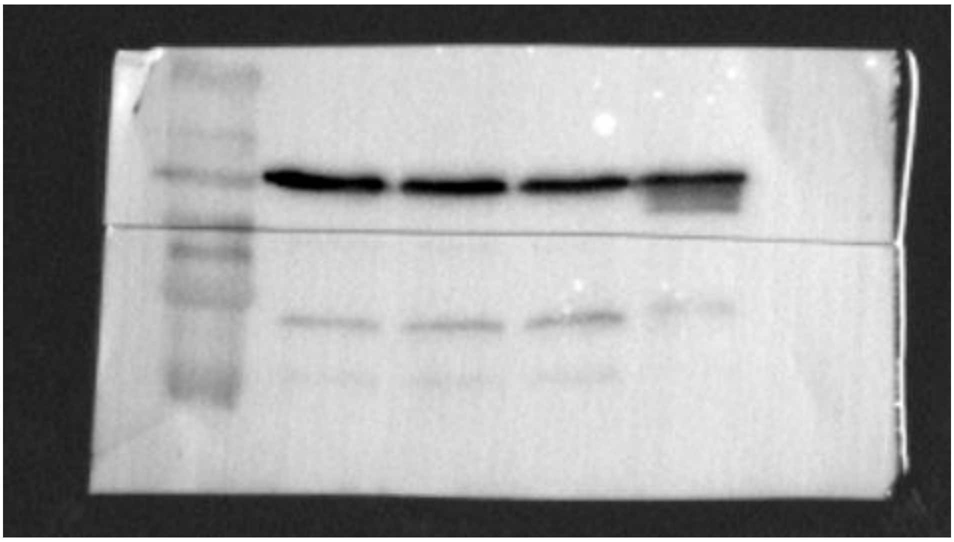

（Merged）

Western bloting- Nrf2

1：Sham

2：SCI+Veh

3：SCI+AAV-Tim-3

4：SCI+AAV-Tim-3+ Oltipraz

Group 1.

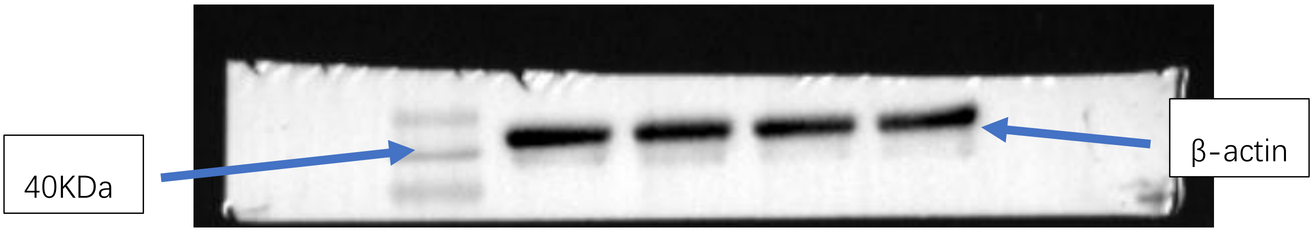

（β-actin KDa） **Figure 8B**

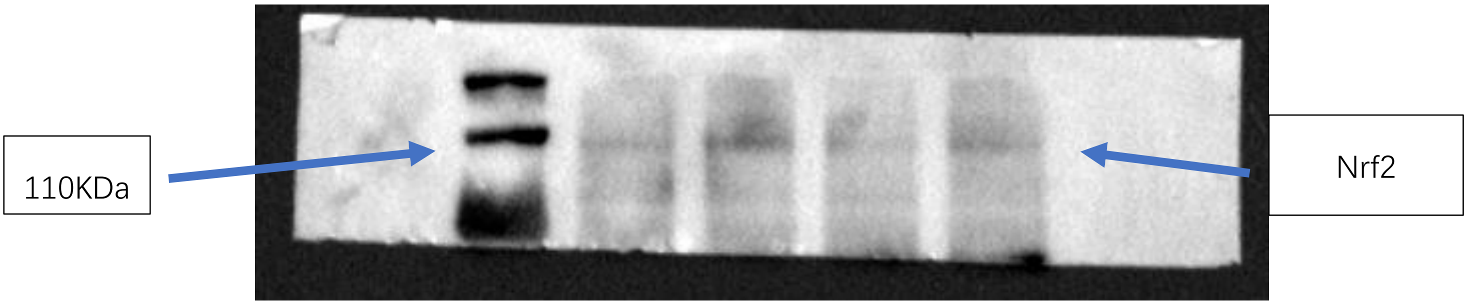

（Nrf2 KDa） **Figure 8B**

Nrf2

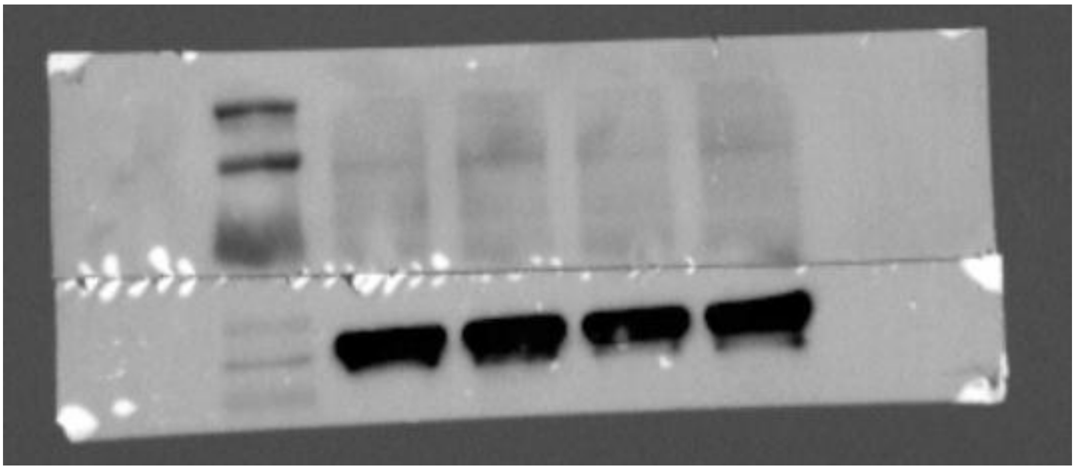

(Merged)

Group 2.

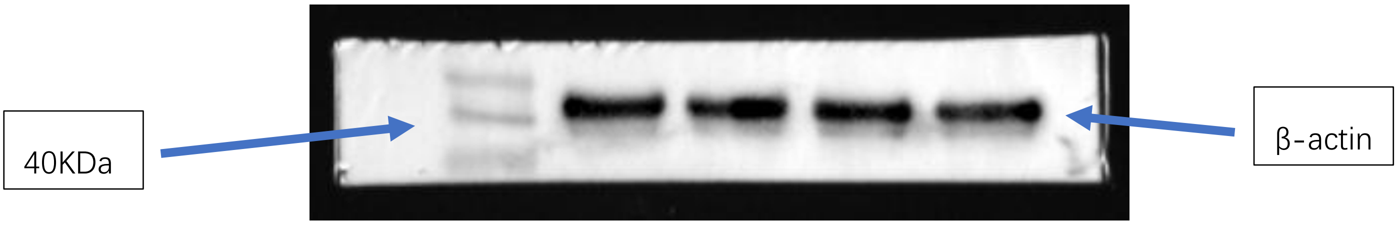

(β-actin KDa)

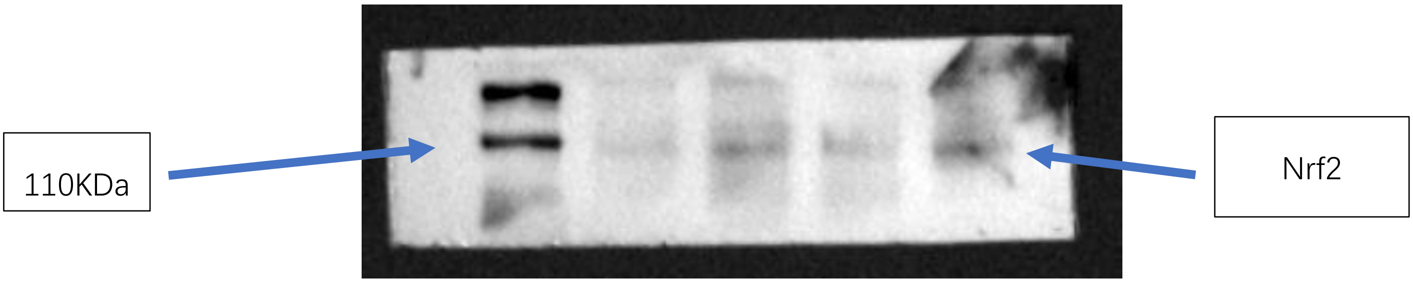

(Nrf2 KDa)

Nrf2

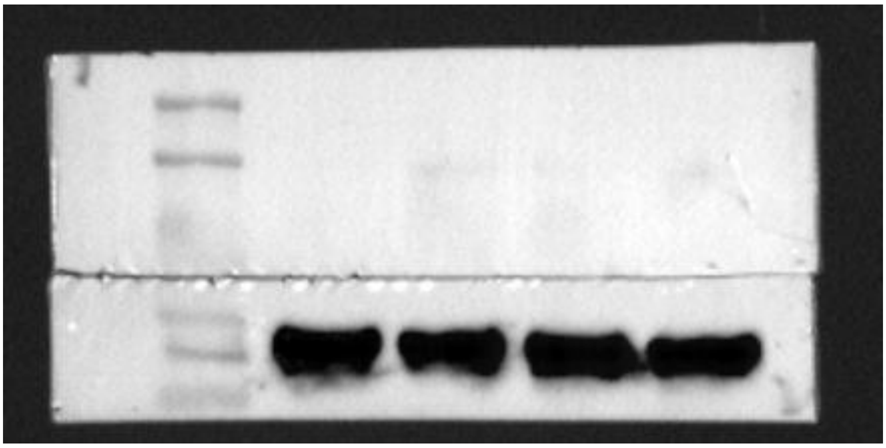

（Merged）

Group 3.

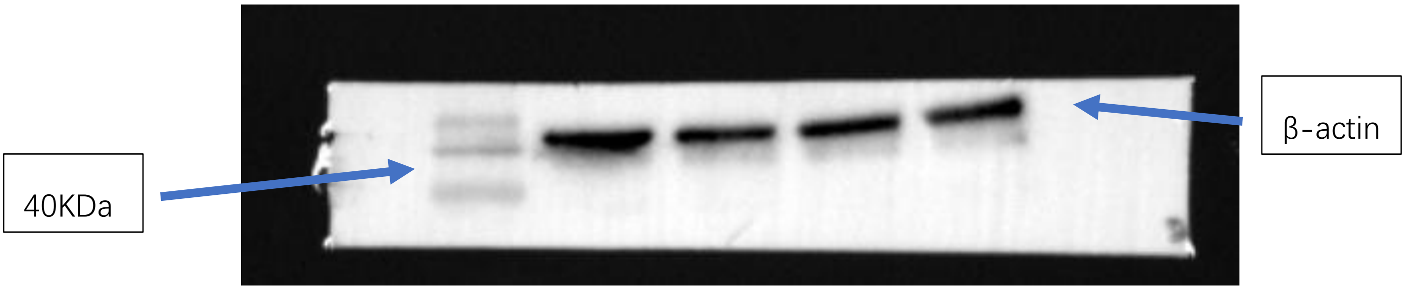

（β-actin KDa）

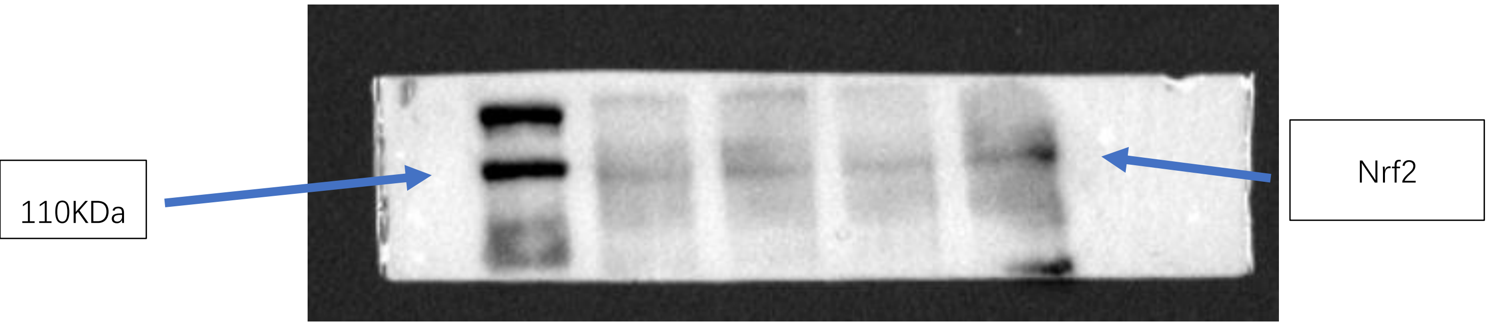

（Nrf2 KDa）

Nrf2

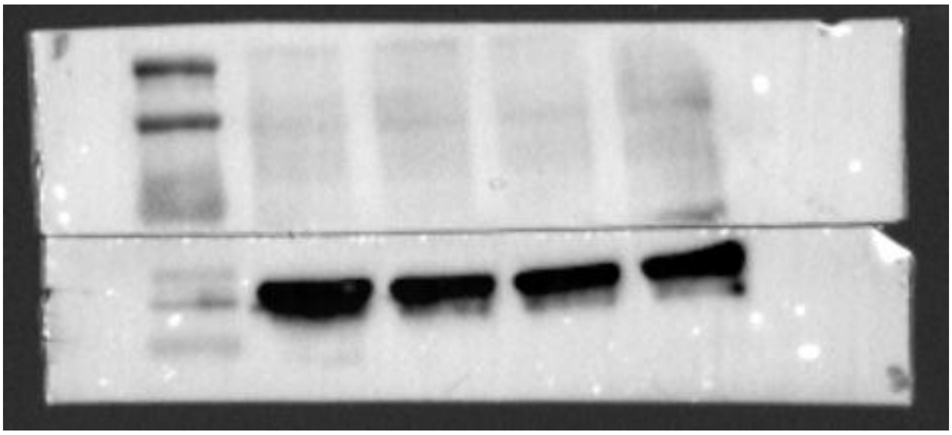

(Merged)

Group4.

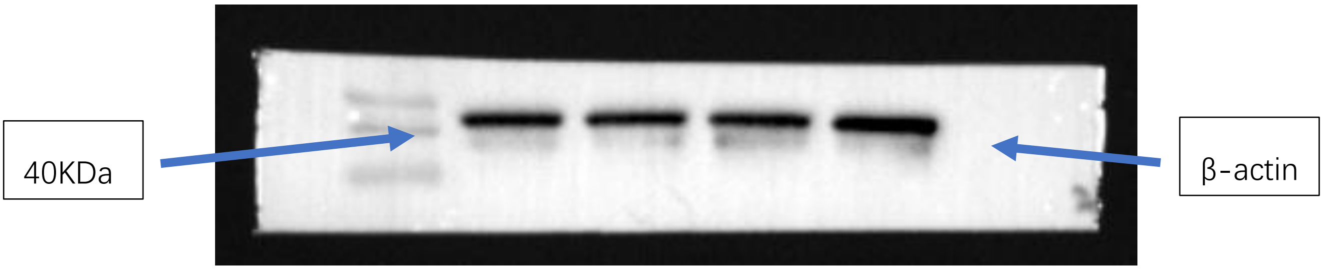

(β-actin KDa)

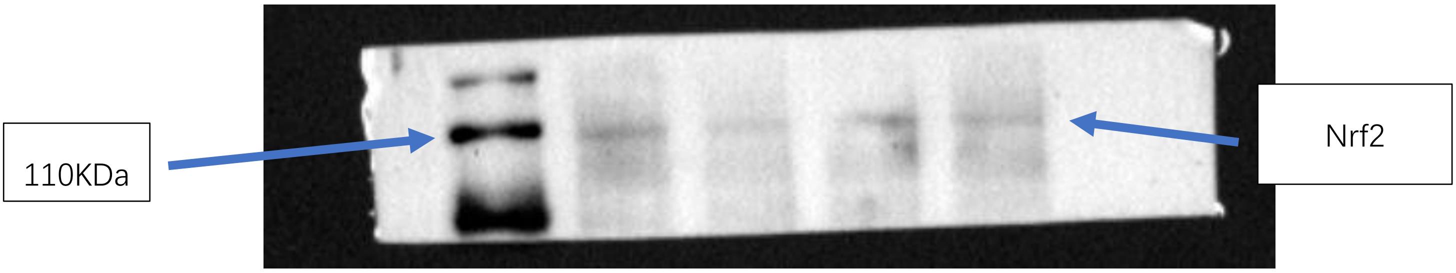

(Nrf2 KDa)

Nrf2

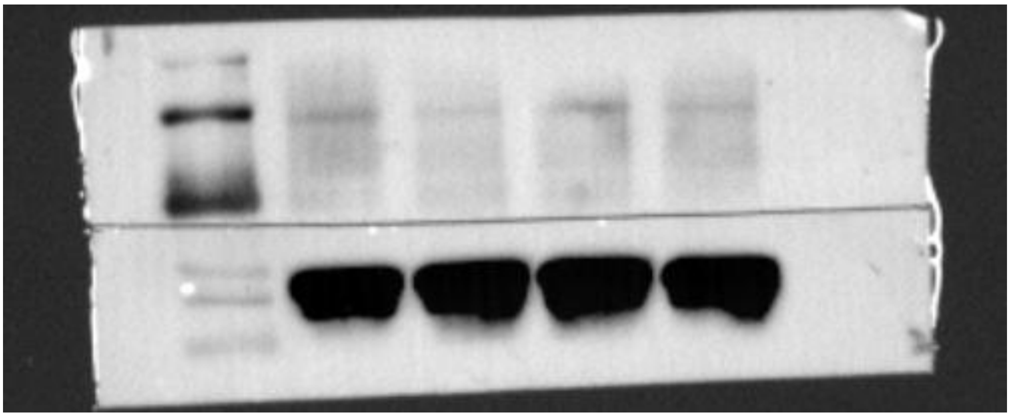

(Merged)

Group 5.

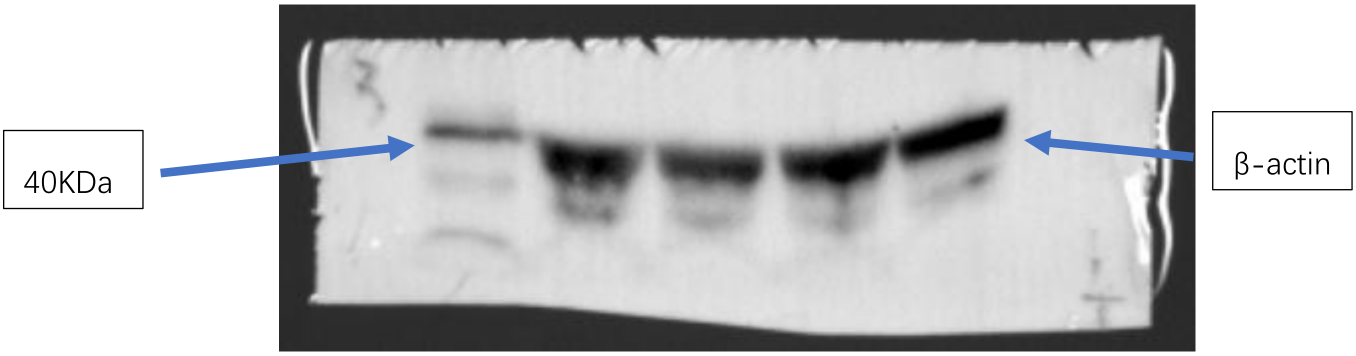

(β-actin KDa)

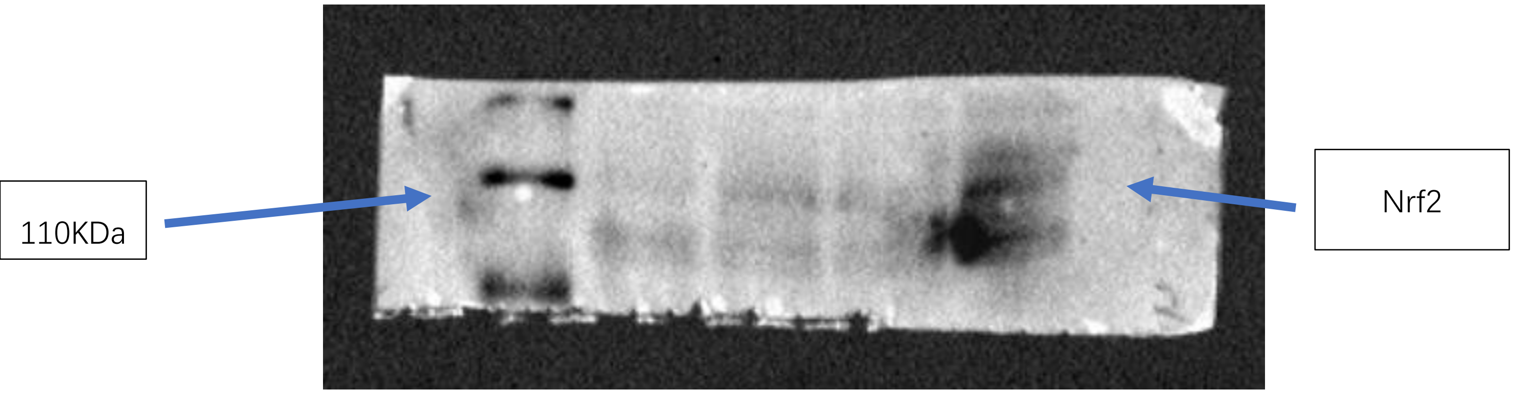

(Nrf2 KDa)

Nrf2

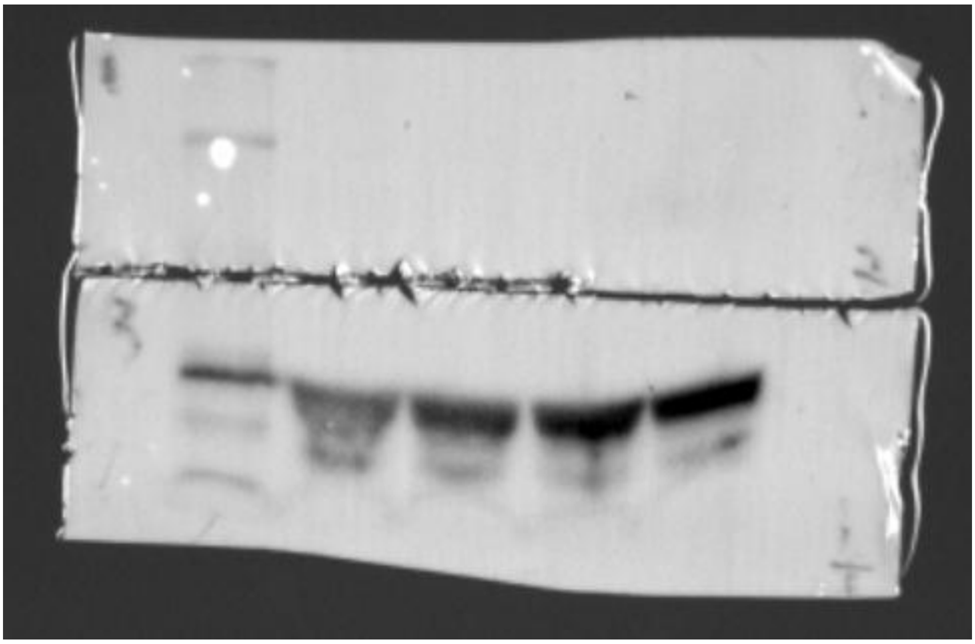

(Merged)

Western bloting-Tim-3

1： Sham

2： SCI+Veh

3： SCI+AAV-NC

4： SCI+AAV-Tim-3

Group 1.

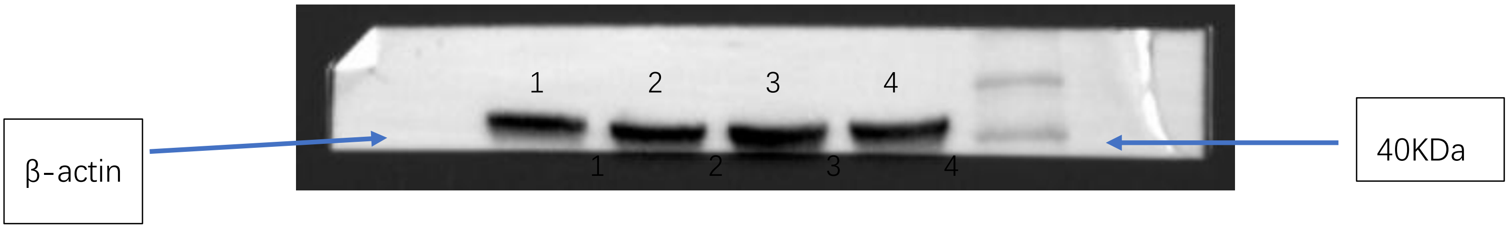

(β-actin KDa) **Figure 2A**

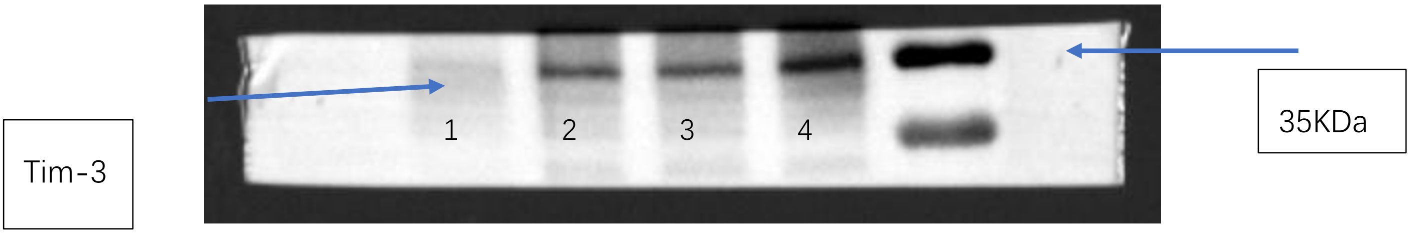

(Tim-3 KDa) **Figure 2A**

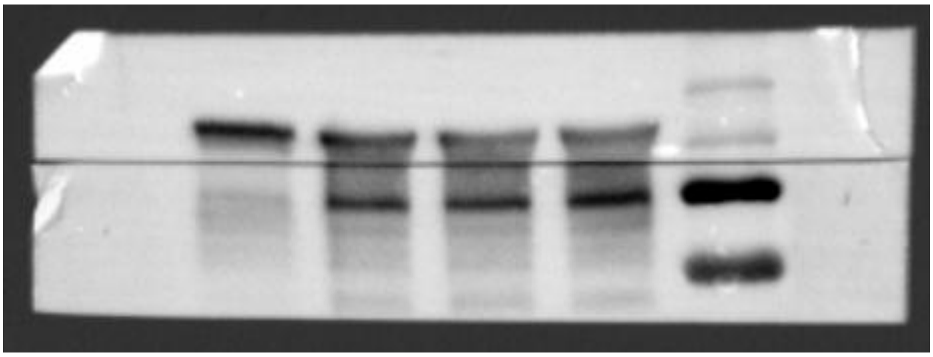

(Merged)

Group 2.

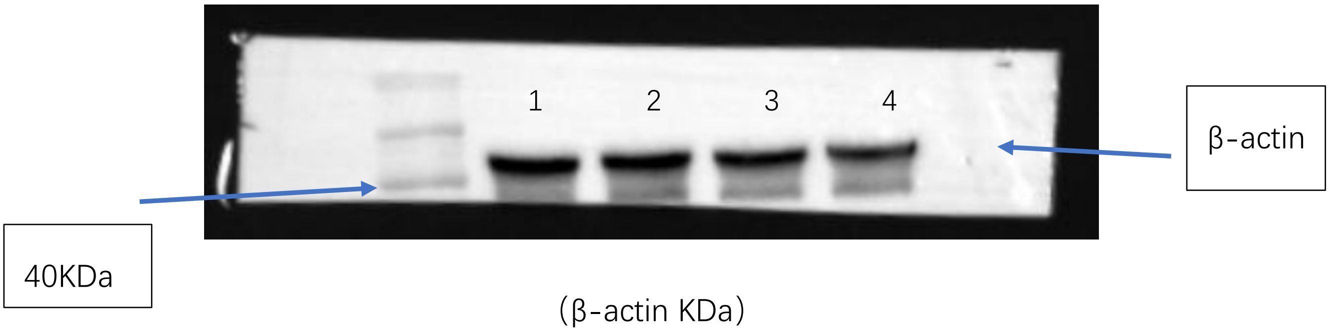

(β-actin KDa)

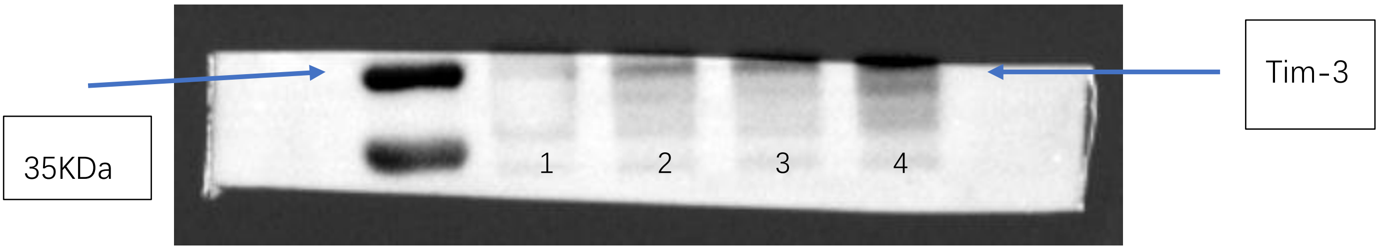

(Tim-3 KDa)

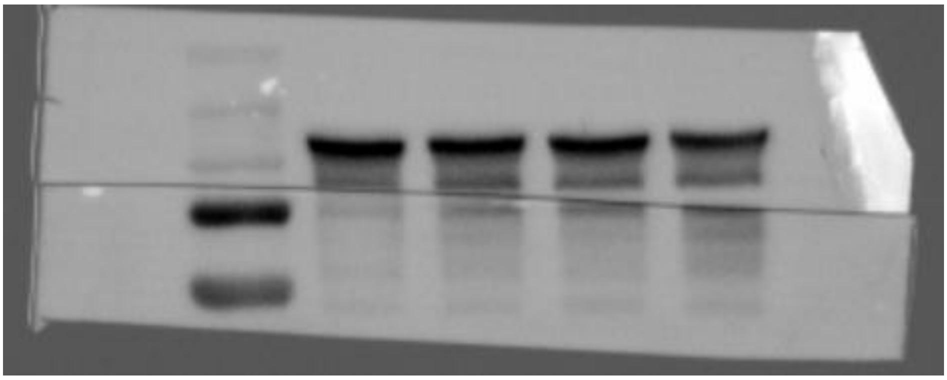

(Merged)

Group 3.

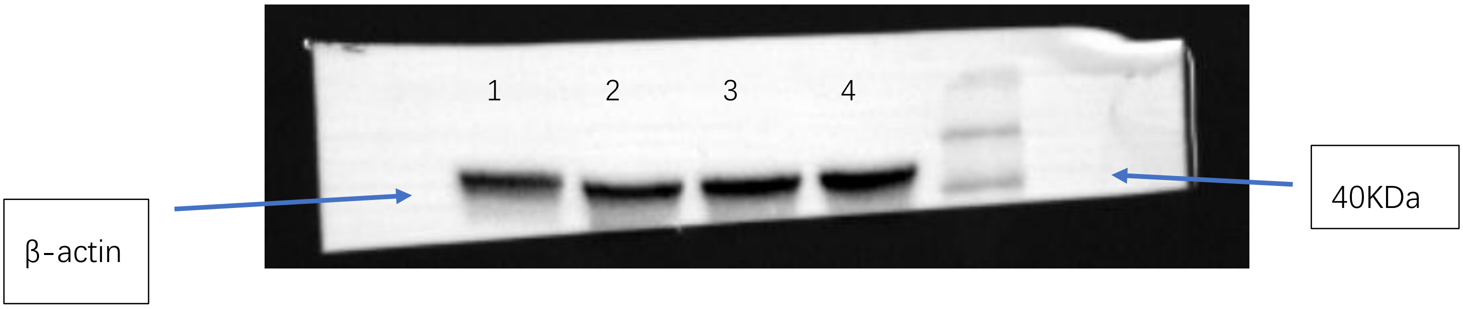

(β-actin KDa)

Tim-3

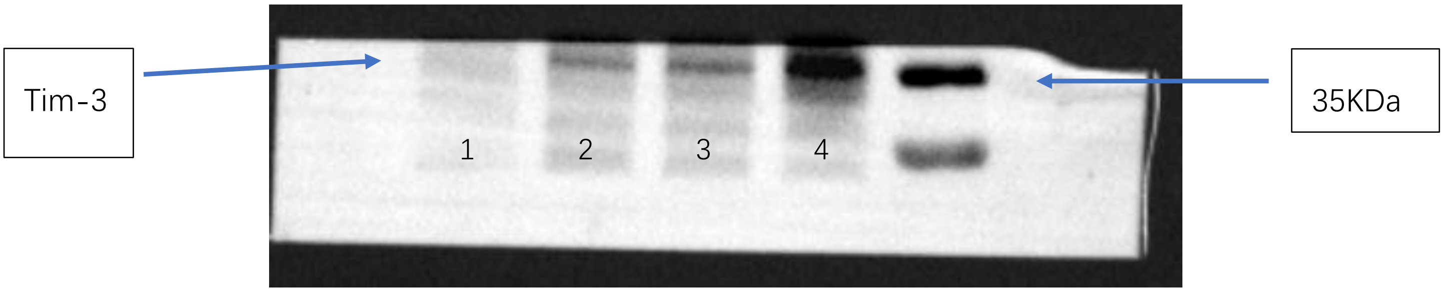

(Tim-3 KDa)

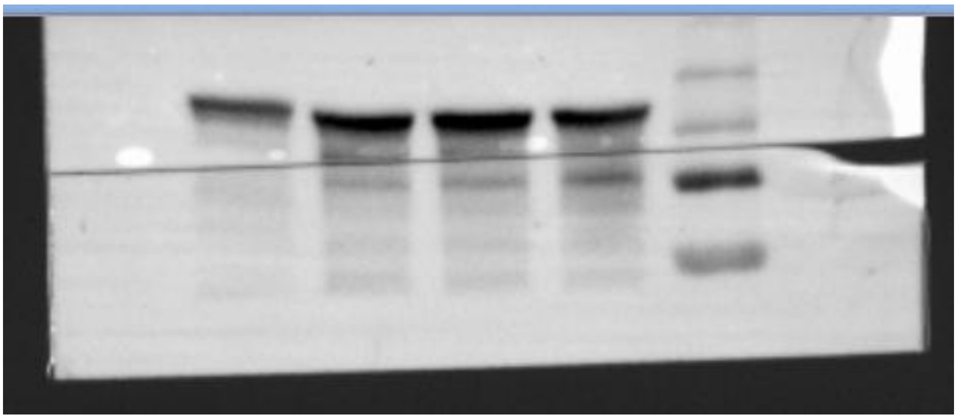

(Merged)

Western bloting-TNF-α

1：Sham

2：SCI+Veh

3：SCI+AAV-NC

4：SCI+AAV-Tim-3

Group 1.

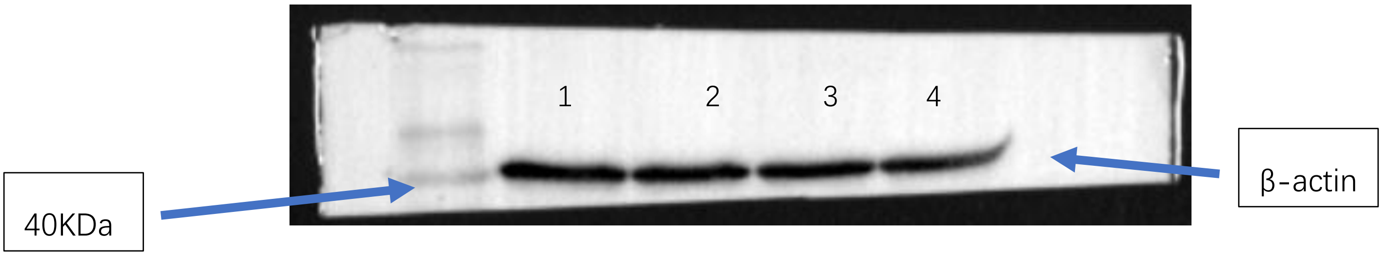

(β-actin KDa)

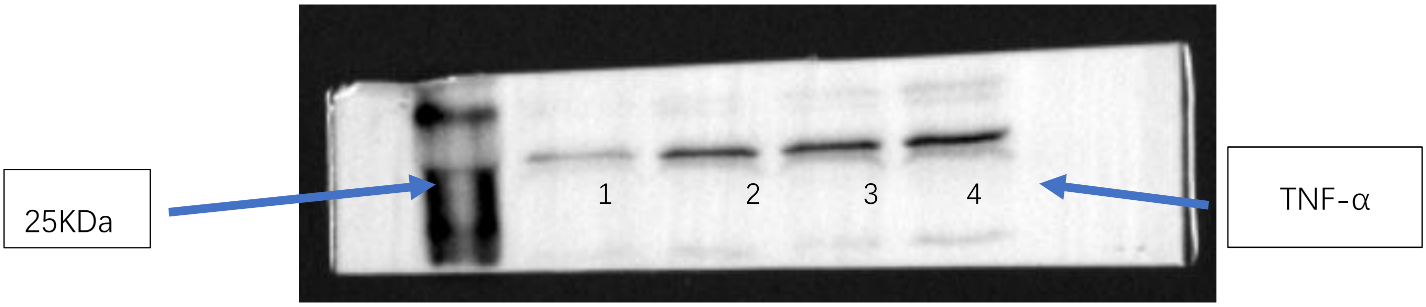

(TNF-α KDa)

TNF-α

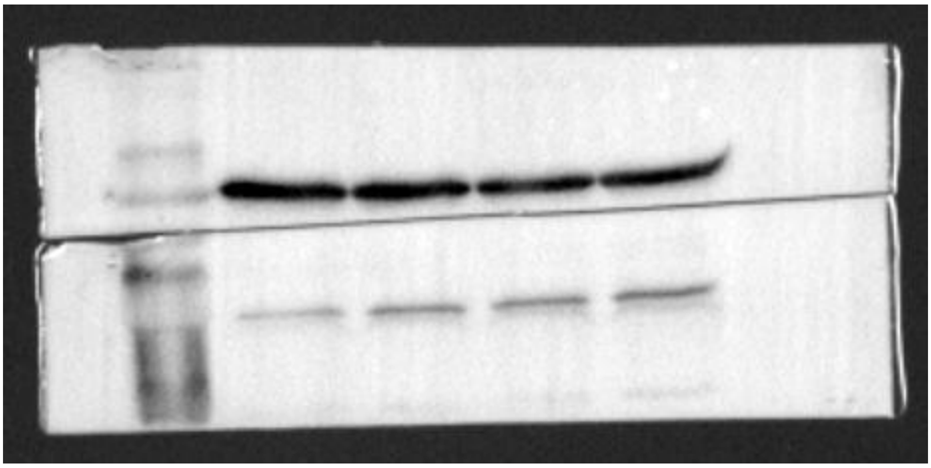

(Merged)

Group 2.

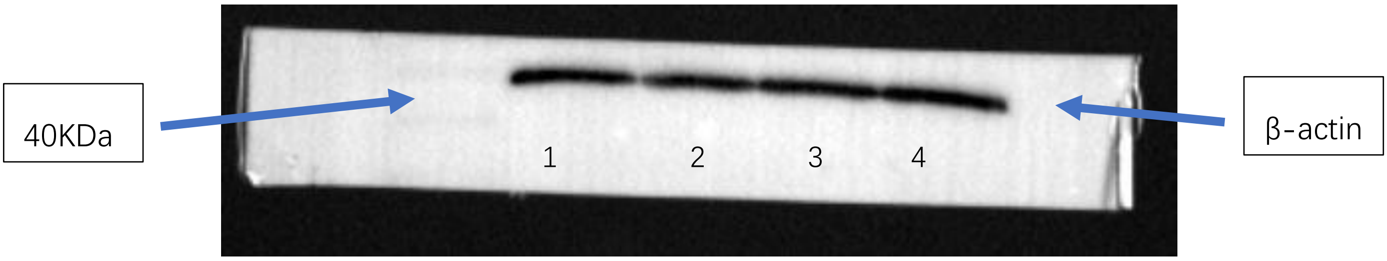

(β-actin KDa) **Figure 6A**

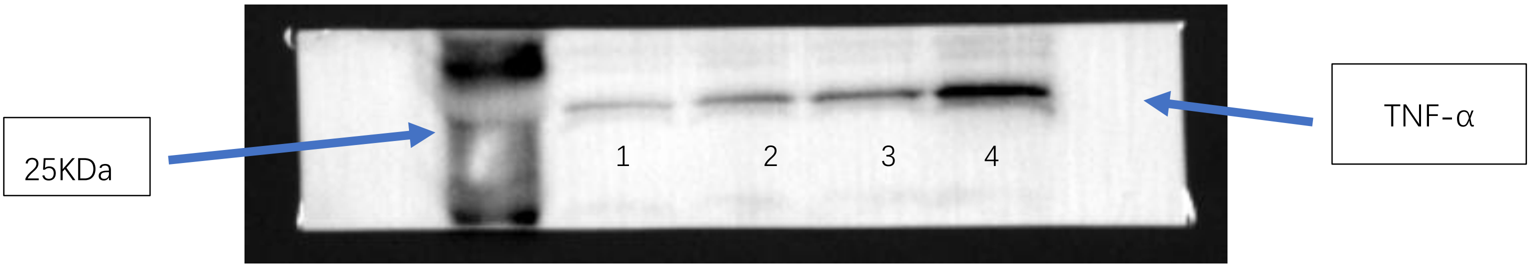

(TNF-α KDa) **Figure 6A**

TNF-α

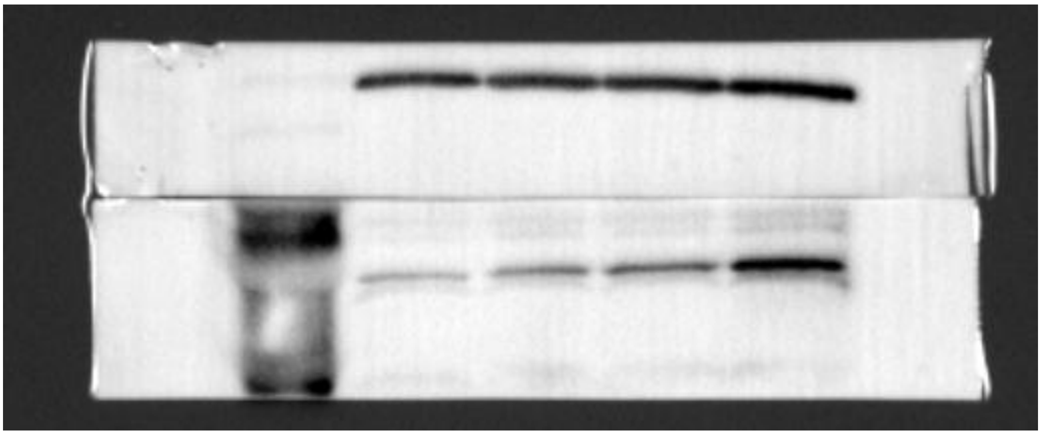

(Merged)

Group 3.

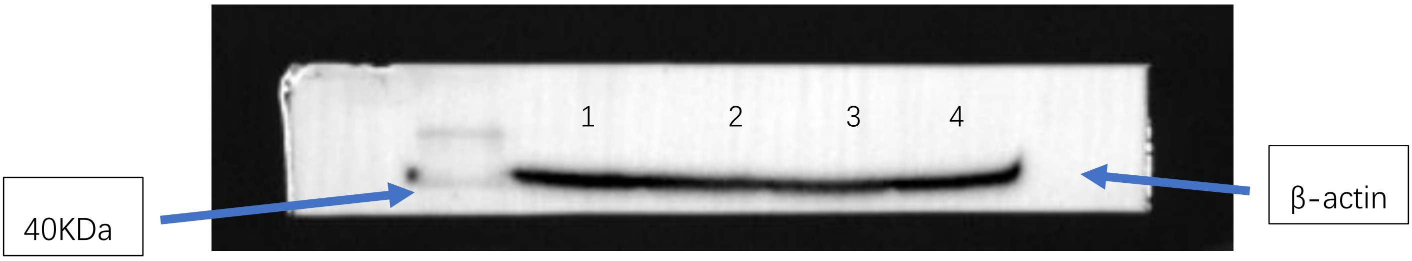

(β-actin KDa)

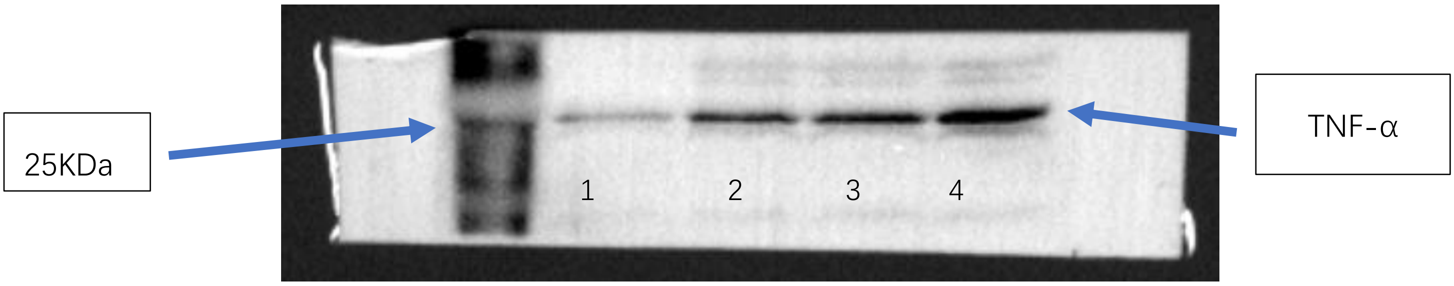

(TNF-α KDa)

TNF-α

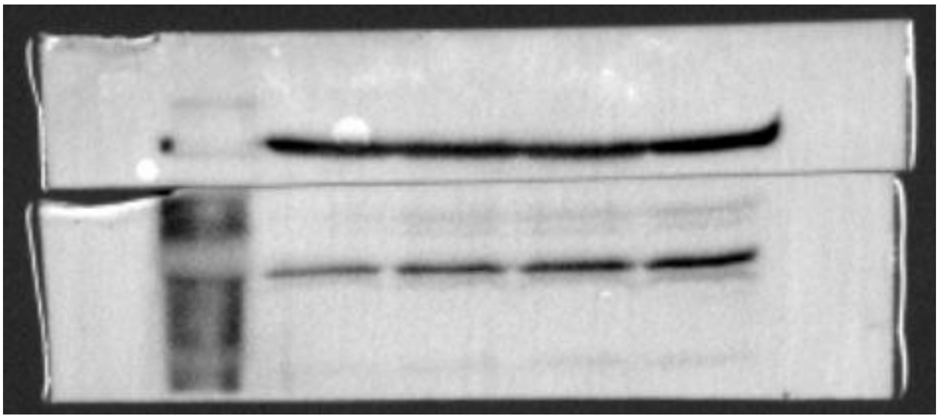

(Merged)

Group 4.

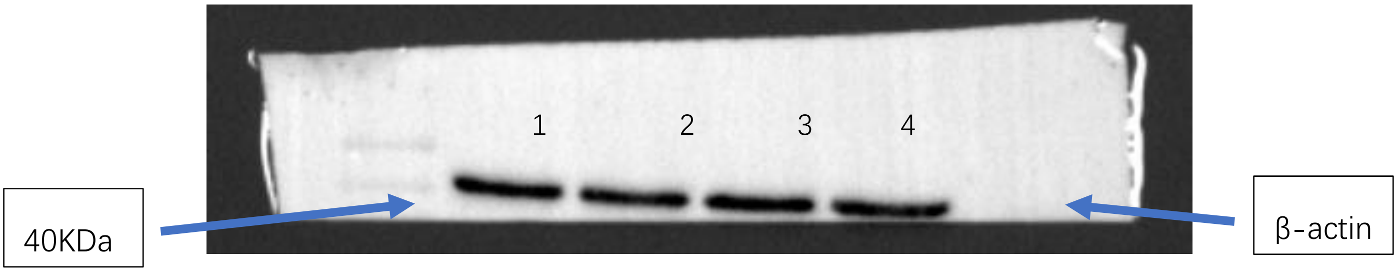

(β-actin KDa)

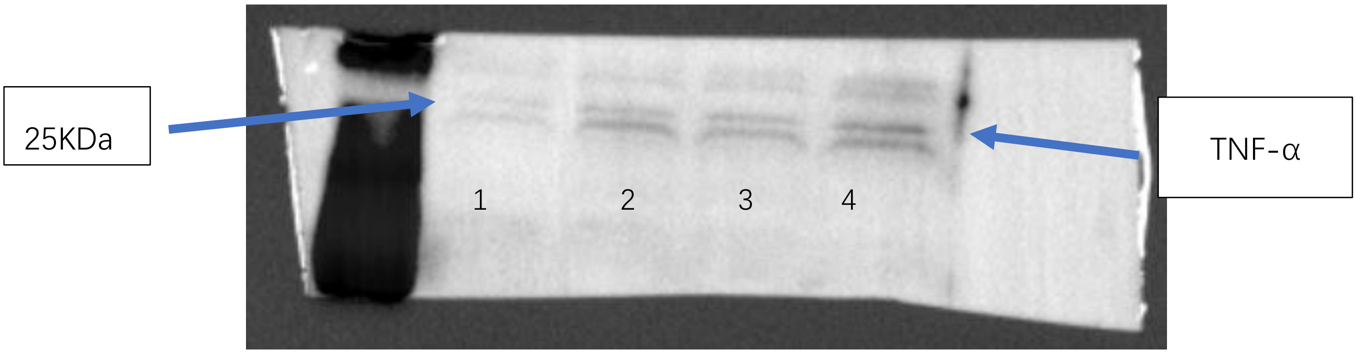

(TNF-α KDa)

TNF-α

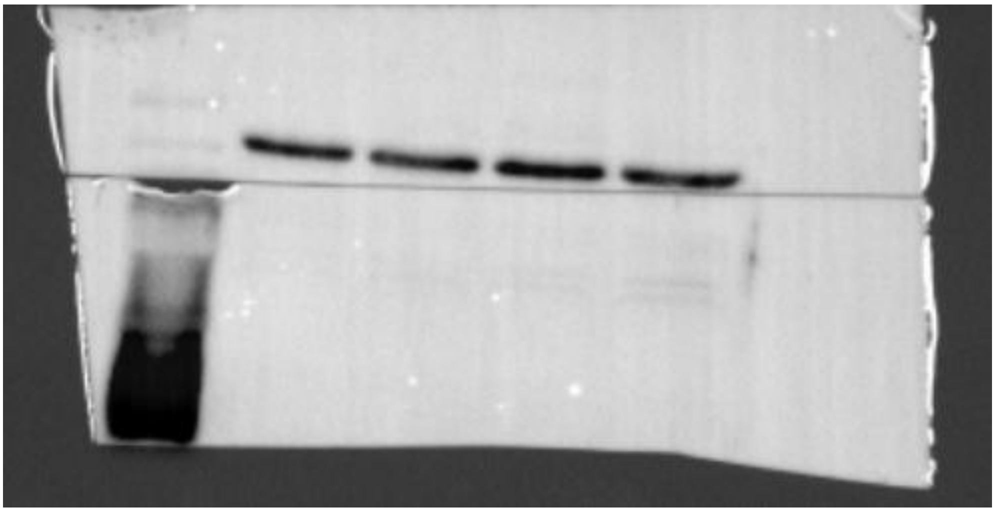

(Merged)

Group 5.

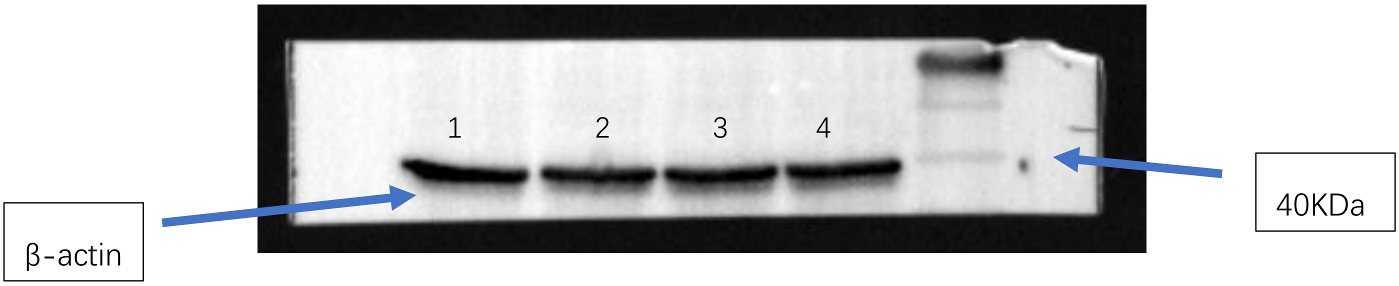

(β-actin KDa)

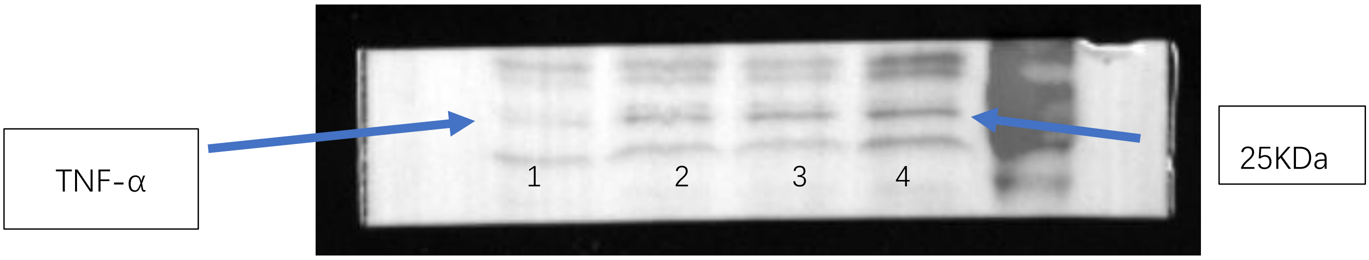

(TNF-α KDa)

TNF-α

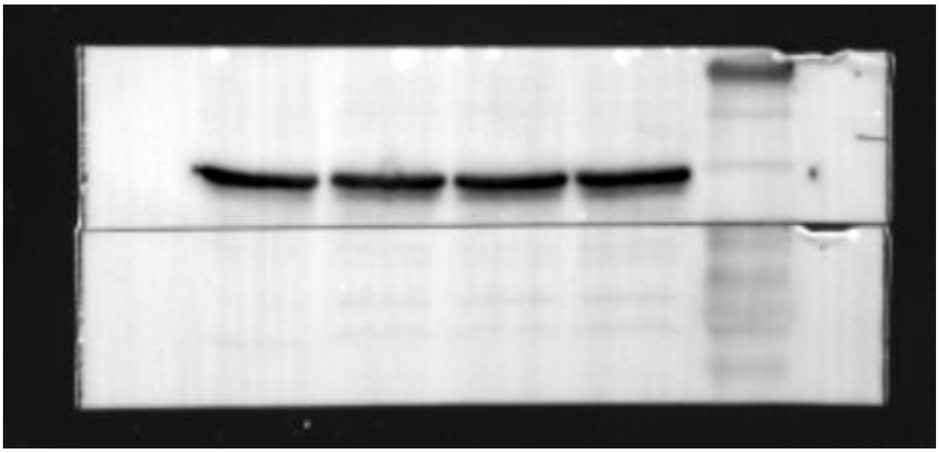

(Merged)
